# Structural and evolutionary divergence of eukaryotic protein kinases in Apicomplexa

**DOI:** 10.1186/1471-2148-11-321

**Published:** 2011-11-02

**Authors:** Eric Talevich, Amar Mirza, Natarajan Kannan

**Affiliations:** 1 Institute of Bioinformatics, University of Georgia, Athens, GA 30602, USA; 2 Department of Biochemistry, University of Georgia, Athens, GA 30602, USA

## Abstract

**Background:**

The Apicomplexa constitute an evolutionarily divergent phylum of protozoan pathogens responsible for widespread parasitic diseases such as malaria and toxoplasmosis. Many cellular functions in these medically important organisms are controlled by protein kinases, which have emerged as promising drug targets for parasitic diseases. However, an incomplete understanding of how apicomplexan kinases structurally and mechanistically differ from their host counterparts has hindered drug development efforts to target parasite kinases.

**Results:**

We used the wealth of sequence data recently made available for 15 apicomplexan species to identify the kinome of each species and quantify the evolutionary constraints imposed on each family of apicomplexan kinases. Our analysis revealed lineage-specific adaptations in selected families, namely cyclin-dependent kinase (CDK), calcium-dependent protein kinase (CDPK) and CLK/LAMMER, which have been identified as important in the pathogenesis of these organisms. Bayesian analysis of selective constraints imposed on these families identified the sequence and structural features that most distinguish apicomplexan protein kinases from their homologs in model organisms and other eukaryotes. In particular, in a subfamily of CDKs orthologous to *Plasmodium falciparum *crk-5, the activation loop contains a novel PTxC motif which is absent from all CDKs outside Apicomplexa. Our analysis also suggests a convergent mode of regulation in a subset of apicomplexan CDPKs and mammalian MAPKs involving a commonly conserved arginine in the *α*C helix. In all recognized apicomplexan CLKs, we find a set of co-conserved residues involved in substrate recognition and docking that are distinct from metazoan CLKs.

**Conclusions:**

We pinpoint key conserved residues that can be predicted to mediate functional differences from eukaryotic homologs in three identified kinase families. We discuss the structural, functional and evolutionary implications of these lineage-specific variations and propose specific hypotheses for experimental investigation. The apicomplexan-specific kinase features reported in this study can be used in the design of selective kinase inhibitors.

## Background

The parasitic protists which comprise the phylum Apicomplexa are responsible for human diseases of global importance, such as malaria (caused by *Plasmodium falciparum *and other members of the *Plasmodium *genus), cryptosporidiosis (*Cryptosporidium *species) and toxoplasmosis (*Toxoplasma gondii*), as well as the agricultural diseases babesiosis (*Babesia bovis *in cattle) and coccidiosis (*Eimeria tenella *in chickens) [1]. In recent years, understanding of the molecular biology and evolution of this phylum has improved dramatically; yet effective treatments for these diseases are still elusive, and there remains an urgent need for deeper research into the basic biology of apicomplexans [2].

Several traits make these pathogens difficult to target therapeutically. As eukaryotes, they share a number of pathways with their mammalian and avian hosts; as intracellular parasites, they have been observed to quickly develop resistance to pharmaceutical treatments [3]. The identification of distinctive protein features which appear conserved across apicomplexan species, but not in their hosts, however, will aid the search for potential new targets for selective inhibition that are more likely to be safe and effective [4]. As protein kinases have been successfully targeted for inhibition in cancer, this diverse protein superfamily warrants consideration as a target for parasitic diseases as well [2,5].

Recent whole-genome sequencing efforts have targeted a number of apicomplexan species [6-17]. Several analyses of protein kinases in these organisms, in particular, have pointed out key signaling pathways [18-20], instances of expansion and loss of kinase gene families [21,22], and emergence of novel protein kinase families [21,23,24], thus providing important insights into biological functions. These comparative studies have furthermore proposed hypotheses which have subsequently been validated by functional and structural studies [19,20,25,26].

The eukaryotic protein kinase (ePK) superfamily is classified into several major groups, corresponding to broad functional categories with distinguishing sequence and structural features [27,28]. The presence of specific ePK groups and families in a genome is a key indicator of biological functions critical for an organism; likewise, missing groups or families indicate functions less critical for an organism's survival and reproduction. These proteins, and the fundamental cell processes in which they participate, are well characterized in humans and several model organisms [28].

Previous efforts to perform detailed comparative analysis of apicomplexan kinases have largely focused on the kinomes of individual species within the genera *Plasmodium*, *Toxoplasma *and *Cryptosporidium *[10,11,20,29-32]. Thus, there is no global overview of the sequence and structural features that distinguish apicomplexan kinases collectively from their metazoan counterparts.

Sequence data from 15 apicomplexan species and several crystallographic structures of a variety of apicomplexan protein kinases are now available. We can use these data to perform a systematic comparison of protein kinases in apicomplexans and model eukaryotes to identify broadly conserved orthologous groups and distinctive residue-level differences.

In this study we use a bioinformatics approach to comprehensively analyze genomic and structural data sets. We perform an exhaustive comparison of apicomplexan kinomes, providing broad coverage of the phylum. We also perform a quantitative, residue-level analysis of the differences between kinases within the Apicomplexa and those in model eukaryotes, in particular humans. We use a Bayesian method [33] to rigorously quantify sequence differences between homologous protein kinases in apicomplexans and other eukaryotes, and reveal contrastingly conserved features that were not apparent previously. Where possible, we then place these sequence features in structural context to postulate specific hypotheses for experimental testing.

Our specific findings include: (i) a detailed accounting of the lineages in which the apicomplexan-specific kinase families FIKK and ROPK appear; (ii) a unique apicomplexan-specific subfamily of cyclin-dependent kinases (CDK), orthologous to *P. falciparum *crk-5, and the motifs that distinguish it; (iii) a hypothesized mechanism of activation by phosphorylation, resembling that of MAP kinases, in a chromalveolate-specific subfamily of calcium-dependent protein kinases (CDPK); and (iv) a description of the adaptation of the substrate-recognition and docking sites in the CLK kinase family in a clade including apicomplexans and other chromalveolates, revealed by the co-evolution of a small set of key residues.

## Results and Discussion

We identified and classified the eukaryotic protein kinases in a total of 17 genomes from 15 species, as well as the solved apicomplexan ePK structures in the Protein Data Bank [34]. We used our classification to broadly describe the conserved ePK families in the Apicomplexa and then performed a residue-level analysis of the lineage-specific differences within several conserved families: CDK, CDPK and CLK. We place our findings in the context of the known evolutionary history of apicomplexans and their relatives.

### Kinome classification and composition: Variations within the Apicomplexa

Recent published evolutionary relationships of eukaryotes provide the basis for our genomic comparison [35]. In this study we have chosen model organisms representing major evolutionary splits — the emergence of Chromalveolata (a proposed super-kingdom of plastid-containing eukaryotes [36]), Alveolata (the kingdom comprising ciliates, dinoflagellates and apicomplexans [37]), and Apicomplexa — to illuminate the origin and divergence of the major ePK groups. For genomic comparison we use the parasitic dinoflagellate *Perkinsus marinus *as an outgroup to the Apicomplexa, the photosynthetic diatom *Thalassiosira pseudonana *as an outgroup to the Alveolata, and the yeast *Saccharomyces cerevisiae *as an outgroup to the Chromalveolata. Detailed kinase annotations are given for each genome in Additional File 1.

#### Apicomplexan kinome sizes are comparable to those of other unicellular protists

The number of ePKs identified in each of the surveyed apicomplexan genomes varies, with the coccidians (*Toxoplasma gondii*, *Neospora caninum *and *Eimeria tenella*) containing more ePKs than the haemosporidians (*Plasmodium *spp.), and the piroplasms (*Babesia bovis *and *Theileria *spp.) containing fewer (Table 1). *Cryptosporidium *spp., the most basal group of apicomplexans considered here, contain a similar number of ePKs to *Plasmodium *spp.

**Table 1:** Kinome sizes

Species	ePKs	Genes	Ratio
*Plasmodium berghei*	69	4904	1.41%
*Plasmodium chabaudi*	70	5131	1.36%
*Plasmodium yoelii*	62	5878	1.05%
*Plasmodium knowlesi*	65	5197	1.25%
*Plasmodium vivax*	65	5435	1.20%
*Plasmodium falciparum*	93	5491	1.69%
*Theileria annulata*	42	3793	1.11%
*Theileria parva*	43	4035	1.07%
*Babesia bovis*	43	3671	1.17%
*Toxoplasma gondii *GT1	137	8102	1.69%
*Toxoplasma gondii *ME49	146	7993	1.83%
*Toxoplasma gondii *VEG	133	7846	1.70%
*Neospora caninum*	141	7082	1.99%
*Eimeria tenella*	90	8786	1.02%
*Cryptosporidium hominis*	65	3886	1.67%
*Cryptosporidium parvum*	75	3805	1.97%
*Cryptosporidium muris*	77	3934	1.96%
*Perkinsus marinus*	251	23654	1.06%
*Thalassiosira pseudonana*	140	11673	1.20%
*Saccharomyces cerevisiae*	116	5797	2.02%

Total proteome and protein kinome sizes in each genome. Columns indicate species name, the number of ePKs found using our method, the number of protein-coding genes in each genome, and the calculated proportion of ePKs in each genome for comparison. Atypical protein kinases are excluded from all ePK counts.

Taken as a percentage of total genome size, the proportions of kinases in apicomplexans are generally either comparable to the 2% observed in yeast and humans [28], as seen in the coccidians and *Cryptosporidium*, or reduced, as in the piroplasms and *Plasmodium *(Table 1). (Note that the quality of genome assemblies and gene model annotations varies, and these differences can affect the number of genes and kinases identified in each genome; the low kinase-to-gene ratios given for *P. yoelii *and *E. tenella *should therefore be interpreted with caution.) There is no evidence of the striking overall expansion of kinases seen in free-living ciliates such as *Paramecium tetraulia *(ePKs 6.6% of the genome [38]), which form a sister clade to Apicomplexa within the kingdom Alveolata. Rather, the number of kinases appears to scale with the total number of protein-coding genes in each genome, with small deviations.

Except for the coccidians and *P. falciparum *(which each contain dramatic expansions of novel kinase families, discussed below), the absolute number of kinases in each apicomplexan genome is markedly reduced relative to free-living eukaryotes (Table 1). The piroplasm kinome sizes, for instance, are less than twice the minimal kinome of 29 ePKs exhibited by another obligate intracellular parasite, *Encephalitozoon cuniculi *[39]. The pattern of genome compaction, occasionally offset by lineage-specific expansions of specific gene families, has been noted as a common mode of genomic evolution in unicellular pathogens [40] and apicomplexans specifically [41,42]. Evidently, the ePKs have evolved according to some of the same adaptive strategies as the overall genomes of these parasites.

### Survey of ePK major groups

**Figure 1:**
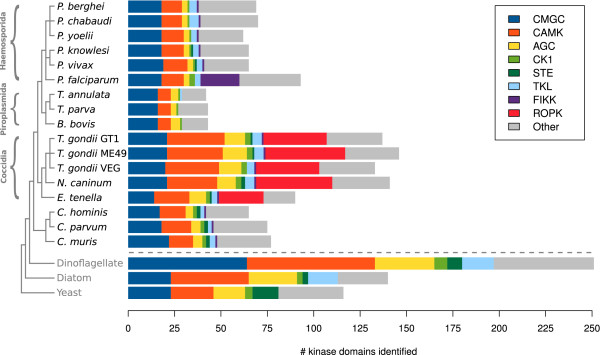
**Kinome group composition**. Composition of protein kinase major groups and selected apicomplexan-specific families (FIKK and ROPK) in each of the surveyed genomes. The schematic species tree along the left edge is constructed from published sources [36,92-94], and includes three outgroup kinomes for comparison: the dinoflagellate *Perkinsus marinus*, the diatom *Thalassiosira pseudonana*, and the yeast *Saccharomyces cerevisiae*. In the stacked bar chart associated with each genome, block width indicates number of genes found belonging to each major group of eukaryotic protein kinases; total bar width indicates total kinome size.

We classified the kinases in each of the surveyed apicomplexans and model organisms according to a hierachical scheme based on seven major ePK groups, enabling a direct comparison of the group composition between kinomes (Figure 1). The CMGC and CAMK groups are especially well conserved across eukaryotes, indicating that the cell functions performed by these proteins are fundamental and essential for eukaryotic life. The casein kinase 1 group (CK1) is conserved in at least one copy among all eukaryotes as well. The tyrosine kinase (TK) and receptor guanylate cyclase (RGC) groups are entirely missing from the Apicomplexa, which has previously been noted [24,43], as well as the three outgroup genomes. There is an apparent reduction, relative to the outgroup *P. marinus *and *T. pseudonana*, of the cyclic-nucleotide-and calcium/phospholipid-dependent kinases (AGC group) in most of the Apicomplexa (Figure 1). The coccidians have between 9 and 13 members of the AGC group, while other apicomplexans have 3 to 5 AGC kinases; PKA is the only AGC family that is found in every genome (Additional File 1). The additional AGC members in coccidians appear as 1-3 copies of several known families, suggesting that AGCs were mostly lost in the other lineages and conserved or slightly amplified in coccidians, rather than a significant expansion in coccidians relative to the common ancestor. An even more dramatic loss of kinase families along all lineages is apparent in the STE group, which we discuss below. The tyrosine-kinase-like group (TKL) shows greater variation, appearing in some abundance in coccidians and *Plasmodium *spp. but absent from piroplasms, except for a single instance in *T. annulata *(Figure 1). The "Other" group designation collects all the ePK families that share the ePK fold and sub-domain architecture (unlike atypical protein kinases), but do not fall cleanly into any of the recognized major ePK groups found in the human kinome [28]. Many apicomplexan kinases fall in the Other group (Figure 1), reflecting their deep evolutionary divergence from humans, the reference genome for the commonly accepted kinase classification scheme [28]. Atypical protein kinases, such as the ABC and RIO families, were excluded from this analysis.

#### Conservation of cell-cycle-associated kinases (CMGC) in chromalveolates

The CMGC group is named after four protein kinase families it contains: cyclin-dependent kinase (CDK), mitogen-activated protein kinase (MAPK), glycogen synthase kinase (GSK), and cdc-like kinase (CLK, also called LAMMER) [28]. These kinases are involved in various aspects of cell cycle control, and are highly conserved throughout Eukaryota. Though apicomplexans, as obligate parasites, are able to depend on their host for survival, these signaling mechanisms for various aspects of cell cycle control are retained. Their life cycles are generally complex, often involving both a primary and a secondary host, encysted phases, and sudden trigger of reproduction and proliferation in response to some chronological or external stimulus [44]. This seems to suggest elaborate signaling and regulatory mechanisms, and points toward specialization of CMGC kinases in the Apicomplexa [21].

The most abundant family within the CMGC group is CDK; it is found in 3-6 copies in each apicomplexan genome, and 7-11 copies in the outgroup genomes (Additional File 2). The CDC2 subfamily of CDK is found in at least one copy in every genome, while some species contain single instances of additional CDK families. There are also 1-4 CDKs in each genome which could not be classified into known subfamilies, leaving open the possibility of lineage-specific adaptations in these unclassified copies. GSK occurs in 1-3 copies in each apicomplexan genome, and 1-5 in the outgroup genomes (Additional File 2), reflecting an essential and conserved role in cellular function. Likewise, MAPK and casein kinase II*α *(CK2) are present in a small number of copies in each of the apicomplexan and other eukaryotic genomes surveyed. The MAPK subfamily ERK7 is found in a single copy in every apicomplexan genome, while ERK1 is missing from *Plasmodium *spp. and the piroplasms. The RCK family, comprising the MAK and MOK subfamilies, is present in the three outgroup species but missing from the Apicomplexa.

The CLK and SRPK families, and some subfamilies of DYRK, are involved in phosphorylation of splicing factors such as SR proteins [45,46]. We found 2-4 DYRKs in each apicomplexan genome (Additional File 2). The most conserved subfamily of these, PRP4, was found in 1 copy in each genome except *E. tenella*. A plant-specific subfamily of DYRK, called DYRKP, was found only in coccidians and the outgroups *P. marinus *and *T. pseudonana*. We found 1 copy of CLK in every surveyed genome, and SRPK in 1 copy in all except *P. marinus*, which has 3 copies.

The close relationship between CLK, SRPK and DYRK can confound homology-based classification attempts. However, the families can be distinguished by the presence of family-specific inserts [47] and by the replacement of the arginine in the kinase-conserved catalytic "HRD" motif with threonine ("HTD") in CLK and SRPK, and cysteine ("HCD") or alanine ("HAD") in various DYRK subfamilies [48]. The first comprehensive study of an apicomplexan kinome [21] identified 4 putative CLKs in *P. falciparum*, assigning the names PfCLK-1 through PfCLK-4. Our classification confirmed PfCLK-1 [EupathDB:PF14_0431] as a CLK (discussed in detail below). PfCLK-4 [EupathDB:PFC0105w] has recently been characterized as an SRPK [49]. We assigned PfCLK-3 [EupathDB:PF11_0156] to the PRP4 subfamily of DYRK, supported by the presence of the "HAD" motif in the catalytic loop and homology with putative PRP4 kinases in each of the other *Plasmodium *species. Our classifier placed PfCLK-2 [EupathDB:PF14_0408] in the CMGC group but did not find support for a more specific family. The portion of the sequence in kinase subdomain X, which is broadly conserved as the "EHLAMMERILG" in CLKs [50], is "RFIYSIVSYIG" in PfCLK-2 — there is no sequence identity except for the C-terminal glycine. PfCLK-2 has the catalytic loop motif "HCD", characteristic of most DYRK subfamilies. The protein sequence also contains long inserts in the catalytic domain in the same locations as those of SRPK. A recent study of PfCLK-1 and PfCLK-2 [51] confirmed SR protein phosphorylation activity and found that PfCLK-1 is localized primarily to the nucleus of the cell, like most CLKs, but PfCLK-2 is found in both the nucleus and the cytoplasm, as has been observed in SRPKs in other eukaryotes [52]. We suggest that this protein is unique, with characteristics of both the SRPK and DYRK families, and that the regulatory functions suggested by typical CLK family members do not fully describe the roles of PfCLK-2 in the cell. The corresponding ortholog group in OrthoMCL-DB [53] [OrthoMCL:OG5_165485] is specific to the *Plasmodium *genus, further evidence that PfCLK-2 and its orthologs are paralagous to apicomplexan CLKs and have diverged significantly.

#### Distribution of calcium signaling kinases (CAMK) in Eukaryota

Calcium signaling plays an important role in eukaryotic cell biology. Calcium ions serve as important second messengers in signaling pathways, regulated by the calcium- and calmodulin-dependent kinase (CAMK) group [29]. In apicomplexans, calcium signaling regulates motility and other processes associated with host invasion [31].

There are multiple conserved CAMK members in each surveyed genome, though we observed more variation in gene family sizes here than in the CMGC group. We found 19-31 putative CAMK genes in each coccidian genome, 13-16 in *Cryptosporidium *spp., 11-13 in *Plasmodium *spp. and 7 in each piroplasm (Figure 1). The closely related dinoflagellate *P. marinus *has 69 putative CAMK genes, and the more distantly related diatom *T. pseudonana *has 42. This points to a slight overall reduction of CAMK and CAMK-like protein kinases in coccidians, and more dramatic reductions in the other apicomplexan lineages, relative to the dinoflagellate and diatom (Figure 1). This follows with the overall conservation or reduction of total kinome sizes in each of the genomes (Table 1).

The calcium-dependent protein kinase (CDPK) family within CAMK is of particular interest, as its role in parasite invasion has been investigated recently by several teams [19,54,55]. Like plants and some other protists, apicomplexan genomes contain multiple members of the CDPK family [31]. We found 6 CDPKs in *P. falciparum*, 5 in each of the other *Plasmodium *species, 4-5 in the piroplasms, 11-14 in the coccidians and 7-9 in *Cryptosporidium *spp. In *T. gondii *and *N. caninum *there were also 7-10 members of the CAMK group that could not be classified into a known family. The greater number of CDPK copies and unclassified CAMKs in coccidians accounts for most of the apparent expansion of the CAMK group in that lineage relative to other apicomplexans.

#### Loss and divergence of STE kinase families in apicomplexan lineages

The STE group includes a variety of kinases which participate in MAPK signaling cascades upstream from the MAPK protein [28]. The key families in the group are STE20 (MAP4K), STE11(MAPKKK/MAP3K) and STE7 (MAPKK/MEK), which form a phosphoryl signaling cascade terminating with the phosphorylation of a MAPK on its activation loop at a conserved TxY motif [56]. This MAPK cascade is highly conserved in most eukaryotes, so it is surprising that the STE group has been largely lost from the Apicomplexa, as has been noted previously [21,57].

According to our analysis, the STE group is entirely missing from the piroplasms, while in the *Plasmodium *genus only *P. knowlesi *and *P. vivax *each retain a single STE gene which could not be further classified into a known STE family (Figure 1; Additional File 1). There were also unclassified STEs in *T. gondii *strains GT1 and ME49, *E. tenella *and *Cryptosporidium *spp. (Additional File 1). We did not find any STEs in *T. gondii *strain VEG.

The STE11 family was not found in any of the surveyed apicomplexan genomes. One STE20, showing closest resemblence to the FRAY subfamily (homologs of human OSR1), was found in *N. caninum*; the other apicomplexans had none. STE7 instances appear in *N. caninum*, *C. hominis *and *C. parvum*. For comparison, *Perkinsus marinus *contains 1 instance of STE11 and two instances of STE20, in the MST and PAKA subfamilies (homologs of human MST2 and PAK2, respectively) (Additional File 1). The ciliate *Tetrahymena thermophila *has multiple representives of STE11, STE20, STE7, and other STE families [58].

Features of the two MAPKs of *P. falciparum *illustrate how apicomplexans can compensate for the lack of a complete MAP signaling cascade. Pfmap-1 [EupathDB:PF14_0294] was identified as a member of the ERK7 family of MAPK [21], and retains the conserved TxY activation loop motif of most MAPKs. Pfmap-2 [EupathDB:PF11_0147], however, could not be assigned to a known MAPK subfamily in earlier analyses [21,57] or in ours. In Pfmap-2, the activation loop motif TxY is replaced by TSH [59], and we also note a long insert of about 26 amino acids in the activation loop N-terminal to the TSH motif. Orthologs of Pfmap-2 identified in OrthoMCL-DB [OrthoMCL:OG5_138034] appear in each of the apicomplexan genomes surveyed here, and also retain the long insert in the activation loop and a TSH or TGH motif in place of TxY. Pfmap-2 has been shown to be phosphorylated and activated by the kinase Pfnek-1 [EupathDB:PFL1370w] [60], which is not a member of the STE kinase group but in this case appears to be nonetheless serving as a MAP kinase kinase. As with Pfmap-2, orthologs of Pfnek-1 appear in each of the surveyed apicomplexans [OrthoMCL:OG5_129446]. The conservation patterns of these kinases suggest that the observations made of *P. falciparum*'s unique MAPK signaling mechanisms can be applied usefully to other apicomplexans.

#### FIKK, an apicomplexan-specific protein kinase family

FIKK is a divergent protein kinase family initially identified in *P. falciparum*, named for a conserved four-residue motif in the kinase subdomain II [21]. Previous studies have found 21 copies in *P. falciparum *and 6 in *P. reichenowi*, but single instances in other *Plasmodium *genomes, indicating rapid expansion along one branch within the genus [23]. In *P. falciparum*, FIKK proteins are generally exported to the host cell and often localized to the host cell membrane [61]. Recent work has found that some *P. falciparum *FIKKs are targeted to the Maurer's clefts, which are formed from or in connection with the parasitophorous vacuole membrane (PVM) as a transport mechanism and eventually reach the host cell surface [62]. A variety of functional domains have also been discovered in the N-terminal tail of the FIKK kinase domain, suggesting that the kinase domain and export signal allow trafficking of parasite proteins or other molecules to the host cell membrane [23].

In addition to the 21 recognized FIKKs in *P. falciparum *[21,23], we found a single copy of FIKK in every one of the surveyed apicompexan genomes except *Theileria *spp. and *Babesia bovis *(Figure 1). No homologs were found outside the Apicomplexa. The apparent absence of FIKK from the three piroplasm genomes is particularly intriguing. To rule out the possibility that this absence is simply the result of the FIKK gene model having not been included in the available proteomic sequences, we performed an additional search on the full set of translated ORFs from the genomic DNA sequence sets for these three species; again, no FIKK genes were found. The parsimonious conclusion is that the gene was lost along the piroplasmid evolutionary branch. This loss suggests there may be some difference in the physiology of piroplasmids that eliminates the need for the FIKK protein in those species.

We note with some interest that, in the process of entering a host cell, apicomplexans generally envelop themselves in a parasitophorous vacuoule constructed from the host cell membrane. (This is true of all of the species surveyed here.) Unlike *Plasmodium *spp. and most other apicomplexans, however, *Babesia *and *Theileria *species escape from their parasitophorous vacuole shortly after entering the host erythrocyte [11,63]. Thereafter, the piroplasm interacts directly with the host cell cytoplasm, rather than through the membrane of a vacuole, potentially simplifying the signaling machinery needed by the parasite. Piroplasms are also nonmotile and show other reduced functions compared to other apicomplexans [1]. However, more study of the role of FIKKs and the interaction between the PVM and host cell in apicomplexan species outside *Plasmodium *is needed in order to refine this hypothesis.

#### ROPK family is specific to the coccidians

The rhoptries are a collection of vesicular organelles within the apical complex, a distinguishing feature of the Apicomplexa. They appear in all of the apicomplexans surveyed here [1]. During the invasion process, a number of proteins contained in the rhoptries are secreted through the apical complex into the parasitophorous vacuole, and in some cases the host cell cytosol [64]. The rhoptry kinase family (ROPK) comprises the protein kinases targeted to the rhoptry. ROPKs play a major role in the infection mechanism of *T. gondii *[65]; they have been characterized in *T. gondii *and to a lesser extent in *N. caninum *[24]. The sequences of ROPKs are divergent from other ePKs, but most can still be recognized by generic protein kinase search profiles [24]. Most rhoptry kinases appear to be catalytically inactive, lacking at least one residue of the catalytic "KDD" triad (the lysine and asparates normally conserved in ePK subdomains II, VI and VII [27]), but kinase activity has been demonstrated in ROP16 and ROP18 [24,66]. Recent structural studies of ROP2 and ROP8 revealed a unique modification of the N-lobe of the kinase domain, in particular, and suggested important functional roles for these proteins, despite the absence of catalytic activity in these ROPKs [67].

We found the ROPK family only in the coccidian clade (Figure 1). Proteins associated with the rhoptries in other lineages appear to be unrelated to coccidian ROPKs or any other ePK families.

Our analysis included three strains of *T. gondii*, corresponding to the three classes of virulence: GT1 (Type I, high virulence), ME49 (Type II, intermediate virulence), and VEG (Type III, non-virulent) [68]. The most dramatic difference in kinase counts between the three strains of *T. gondii *appears in the ROPK family (Figure 1). We identified 40 ROPKs in *T. gondii *strain ME49, but 29 in GT1 and VEG (Additional File 1). A simple clustering of the sequences (data not shown) did not reveal a clear separation of ME49 ROPK genes that would indicate an expansion in ME49, so the discrepency may instead be due to losses in the other two strains, or simply differences in the quality of genome assembly and annotation.

### Sequence and structural features contributing to functional divergence

Our approach revealed several novel and distinct subfamilies within recognized ePK families. Within each family, we then performed a phylogenetic analysis of the protein sequences of kinase domains from apicomplexans and several diverse model organisms to identify putative ortholog groups that include several apicomplexan species, but no metazoan species. (See Methods.)

Statistical analysis of the sequences using the CHAIN program revealed distinctive sequence and structural features which distinguish apicomplexan kinases from their homologs in other eukaryotes. Specifically, we used each identified apicomplexan-specific ortholog set as a query against a larger "main" set of sequences representing the corresponding kinase family (CDK, CDPK and CLK) taken from diverse eukaryotic species. CHAIN uses a Bayesian MCMC procedure to concurrently (a) partition the "main" set into a "foreground" of sequences that share distinct residue motifs found in the query, a "background" set of sequences that do not share those motifs, and an "intermediate" set that shares only some of the motifs; and (b) identify the alignment columns defining the motifs that distinguish the foreground and background sets [33]. We then used PyMOL [69] and a set of custom scripts leveraging Biopython [70] to map the most significant residue patterns onto aligned protein structures for comparative structural analysis.

Here we describe three proposed instances of lineage-specific divergence of apicomplexan kinases, within the CMGC and CAMK major groups, with an analysis of the sequence motifs and evolutionary histories that define them. Where crystallographic structures have previously been solved, we map sequence motifs onto the 3D structures to gain insight into possible regulatory mechanisms.

### Orthologs of Pfcrk-5 form a novel subfamily of cyclin-dependent kinases

While each apicomplexan kinome contains multiple genes belonging to the cyclin-dependent kinase (CDK) family, we find a novel CDK subfamily which appears in a single copy in 14 of the 17 apicomplexan genomes surveyed, absent only from *Cryptosporidium *spp., and is not found outside Apicomplexa. This subfamily comprises the orthologs of *P. falciparum *Pfcrk-5 [EupathDB:PFF0750w]. This ortholog group is equivalent to a group in OrthoMCL-DB [OrthoMCL:OG5_150603], but with the addition of an ortholog we identified in *Theileria parva *[Genbank:TP04_0791]. A multiple sequence alignment of the subfamily kinase domains, including accession numbers, is given in Additional File 3.

**Figure 2:**
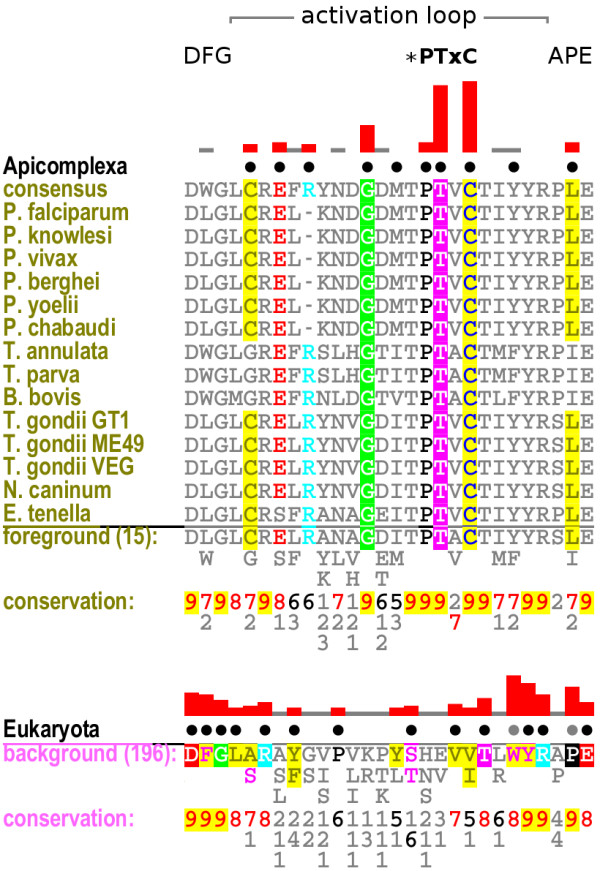
**CHAIN alignment of the CDK subfamily activation loop**. CHAIN alignment of the activation loop in the Pfcrk-5-like CDK subfamily ("Foreground") compared to the corresponding region in a large set of diverse eukaryotic CDK sequences ("Background"). The kinase-conserved DFG and APE motifs bordering the activation loop are indicated at the top, along with the subfamily-conserved PTxC motif. An asterisk indicates the position of the threonine observed to be phosphorylated in other CDKs, conserved in both the foreground and background. The histogram above each sequence set represents the differential levels of conservation between the two sets at each position, using logarithmic scaling. Dots above each alignment column indicate the contrasting conservation pattern determined by CHAIN. Note that the Apicomplexa (foreground, top) and Eukaryota (background, bottom) sets have different conservation patterns. In the sequence alignment itself, columns of the conserved pattern are colored according to the consensus residue type. The consensus residue types are listed below the alignment. Weighted residue frequencies are shown in the following rows, in units of integer tenths (e.g. "9" indicates conservation of 90-100%). The number of sequences in each set are shown in parentheses. A complete CHAIN alignment of these sequences is provided in Additional File 4.

The subfamily is distinguished by a unique PTxC motif in the activation loop (Pfcrk-5 positions 255-258), which is strikingly conserved relative to other CDK members in diverse eukaryotes, and absent from diverse eukaryotic homologs, as determined by CHAIN analysis (Figure 2; Additional File 4). In eukaryotic homologs, the residues at the location of the PTxC motif are most often histidine, glutamate and valine. The threonine in position 254 is also found as either threonine (usually) or serine (more rarely) in homologs; this site is equivalent to T160 in human Cdk2, where phosphorylation of this residue dramatically increases CDK catalytic activity, apparently stabilizing the substrate-binding site by forming a network of hydrogen bonds with surrounding side chains [71].

While T254 is conserved in most CDKs across Eukaryota, the apicomplexan-conserved residues P255, T256 and C258 are strikingly different from those in CDKs of other eukaryotes (Figure 2). In particular, T256 in this subfamily appears most often as a glutamate in other CDKs, including the closest-matching known CDK subfamily, CDC2, though it is not strongly conserved overall in eukaryotic CDKs. Given the similarity in chemical properties between glutamate and phosphothreonine, it is tempting to speculate that T256 is a phosphorylation site in this subset of apicomplexan CDKs. An alternative hypothesis is that the residues in the PTxC motif may provide contact points for the substrate, as has been observed for the equivalent residues in the human homolog Cdk2 [72]. Human Cdk2 belongs to the CDK subfamily CDK2, not CDC2, but contains the motif HEVV in place of Pfcrk-5's PTVC, as most CDC2s do. In a solved structure of human Cdk2 [PDB:1QMZ], the residue V164, equivalent to C258 in Pfcrk-5, is located spatially between the bound substrate and the APE motif. It is possible that C258 in Pfcrk-5 and its orthologs packs hydrophobically against the equivalent region in this subfamily. This could also explain the co-conserved change of the APE motif to PLE (Figure 2). However, the absence of a solved 3D structure for any member of this subfamily prevents further analysis of the functional role of these residues. Although four structures of apicomplexan CDKs have been published [PDB:1V0O, PDB:1V0B, PDB:1OB3, PDB:2QKR], none of them correspond to genes from the Pfcrk-5 subfamily.

To assess whether the members of this putative subfamily should instead be assigned to the known CDK subfamily CDC2, we used CHAIN again to compare this subfamily to sequences representing the CDC2 subfamily. The same distinguishing pattern of PTxC in the activation loop appears in this comparison as well (Additional File 5). In *P. falciparum*, the CDKs Pfcrk-1-4 have all previously been annotated as "cdc2-related" kinases, and have been characterized in previous studies [73,74]. The canonical CDC2 in *P. falciparum*, as identified by our analysis, is protein kinase 5 [EupathDB:MAL13P1.279], which has the more typical "HEVV" motif in place of Pfcrk-5's "PTVC". Thus, the genes in this apicomplexan-specific subfamily appear to be paralogous to the known CDC2 subfamily, and may therefore have unique functional roles.

**Figure 3:**
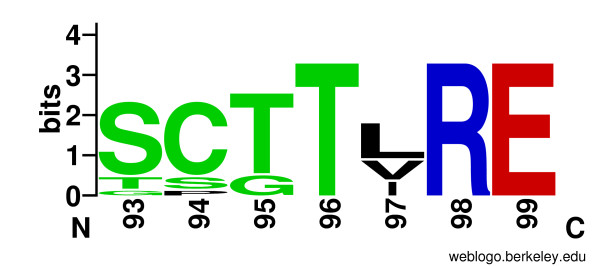
**Logo of the CDK subfamily cyclin-binding motif**. Logo of the aligned activation loop sequences in members of the Pfcrk-5-like CDK subfamily, generated by WebLogo [95]. Letter height represents information content; large letters indicated residues conserved within the subfamily.

Distinct subfamilies of CDK are sometimes named after the conserved residue sequence in the cyclin-binding helix in the N-lobe of the kinase domain, known as the PSTAIRE helix in CDKs or more generally as the *α*C helix in protein kinases [71,73]. In the proposed alveolate-specific subfamily the consensus sequence of the *α*C motif is SCTTLRE, at Pfcrk-5 sequence positions 93-99 (Figure 3). It is not yet known whether Pfcrk-5 is dependent on cyclin binding for activity, like PfPK5, Pfmrk and Pfcrk-3, or independent, like PfPK6 [73,74]. None of these residues appear in the CHAIN pattern, however, indicating that the individual residues at these positions may occur in some non-apicomplexan CDKs as well, and that this motif did not necessarily co-evolve with the activation loop motif that characterizes this apicomplexan-specific subfamily.

We also identify 5 large inserts in the kinase domain which are conserved to varying degrees across all 14 apicomplexan species, but not found in any other known subfamily of CDK. These inserts occur between subdomains I and II, III and IV, IV and V (in the coccidians), VII and VIII (after the conserved PLE, corresponding to APE in most ePKs, and extending over 100 amino acids in *Plasmodium *spp.), and X and XI (an extension of the CMGC insert, normally involved in substrate binding [48]). The inserts appear to be hydrophilic, and are generally conserved at the sequence level within each genus, but less clearly between different genera, indicating rapid evolution relative to the structurally conserved portions of the kinase domain.

### Features of a chromalveolate-specific CDPK subfamily point to a MAPK-like mode of regulation

The CDPK family is characterized in green plants, and instances of it are also recognized in some protists (specifically, chromalveolates), but there are none in metazoans [29,75] — this observation by itself encourages study of the CDPK family as a parasite-specific therapeutic target in human diseases. Each apicomplexan genome contains multiple CDPKs; we find and discuss a novel subfamily of these here. The subfamily is found in all of the surveyed apicomplexans as well as the dinoflagellate *Perkinsus marinus*, the ciliates *Tetrahymena thermophila *and *Paramecium tetraulia*, and the diatoms *Thalassiosira pseudonana *and *Phaeodactylum tricornutum*, indicating that the subfamily is shared by a clade within the Chromalveolata. It includes the *P. falciparum *protein PfCDPK5, which has been shown to play a key regulatory role during the parasite's blood stage [76]. The subfamily does not correspond cleanly to OrthoMCL-DB groups, but contains some members of the main CDPK group [OrthoMCL:OG5_126600] as well as some small lineage-specific groups (e.g. [OrthoMCL:OG5_170347]). Additional File 6 contains a multiple sequence alignment of the kinase domains of all 76 identified subfamily members.

CHAIN analysis highlighted several key residues that distinguish this subfamily from the larger set of chromalveolate CDPKs (Additional File 7), of which two are most striking: an arginine in the *α*C helix, and a threonine or serine in the activation loop. The conservation of these two residues within the subfamily, but not in the broader CDPK family, suggests they have evolved under a shared functional constraint. Notably, the structure of a member of the subfamily in *C. parvum*, CpCDPK2 [EupathDB:cgd7_1840], has been solved in complex with an inhibitor [PDB:3F3Z] and in *apo *form [PDB:2QG5] [32]. The distinguishing residues numbered according to the crystal structures of CpCDPK2 are R69 and T184. Guided by CHAIN analysis, we compared these structures with that of another *C. parvum *CDPK outside the subfamily, CpCDPK1 [EupathDB:cgd3_920, PDB:3DFA], to understand the sequence and structural basis for possible *C. parvum *CDPK functional divergence.

**Figure 4:**
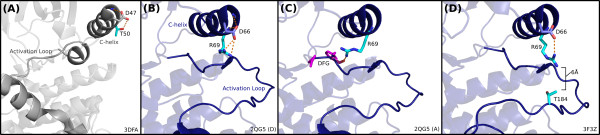
**CDPK subfamily roles of *α*C helix arginine and activation-loop threonine**. Structures of several different CDPKs in *C. parvum*, demonstrating several proposed interactions for the *α*C helix arginine distinctive of an alveolate-specific CDPK subfamily. **(A) **A member of the background set of CDPKs [PDB:3DFA] has a threonine (T50), shown in cyan, in position to form a hydrogen bond with an aspartate (D47), gray, which caps the *α*C helix. This threonine corresponds to the subfamily-conserved arginine; however, the threonine here is not conserved in the background set of CDPKs. **(B) **In a structure of a member of the CDPK subfamily [PDB:2QG5], the subfamily-conserved arginine (R69, cyan) appears similarly positioned to interact with the aspartate (D66, blue) at the end of the *α*C helix, potentially stabilizing the cap. **(C) **Chain A of the same structure shows the distinctive arginine oriented inward, capable of hydrogen-bonding with the kinase-conserved DFG motif (side chains colored magenta). **(D) **In another structure of the same CDPK-subfamily protein [PDB:2QG5], the arginine is positioned toward a subfamily-conserved threonine in the activation loop (T184), shown in cyan. The distance between the R69 and T184 side chains is 6Å, which could accomodate a phosphate group attached to the threonine and a hydrogen bond between the phosphothreonine and the arginine.

We analyzed the structural interactions associated with R69 and T184 in the two available crystal structures of CpCDPK2 [PDB:2QG5, PDB:3F3Z] (Figure 4). In one of the CpCDPK2 structures [PDB:2QG5], R69 adopts two distinct conformations (Figure 4B-D). In chain A, R69 is positioned to form a hydrogen bond to the backbone of a residue (D66) at the *α*C helix N-terminus, while in chain D, R69 appears to form a 3.1Å hydrogen bond to the backbone of the DFG motif glycine, located at the N-terminus of the activation segment. In chain B, R69 is oriented outward, in a solvent-exposed position. (While the CpCDPK2 structure is presented as three chains, the biological unit has not been described.) B-factors and the different orientations of this residue in each chain indicate that the R69 side chain is flexible in this structure.

In the other CpCDPK2 structure [PDB:3F3Z], R69 is oriented toward the side-chain of T184, separated by a distance of 6.0Å. Previous reports show that threonine autophosphorylation in the activation loop is prevalent in apicomplexan CDPKs [30,54]. We therefore hypothesize that this threonine (T184^2*QG*5,3*F*3*Z*^) could also serve as a phosphorylation site in the alveolate-specific CDPK subfamily.

#### Shared features of MAP kinases suggest a common regulatory mechanism

To obtain additional insights into the role of R69 and T184 in CpCDPK2 functions, we identified and analyzed crystal structures of kinases that contain both an *α*C arginine and an activation-loop threonine at positions equivalent to CpCDPK2 R69 and T184, respectively. To allow for the flexibility and variable length of the activation loop, we also examined positions adjacent to T184. This revealed a large number of MAPK structures, including human and mouse p38, where a *α*C-helix arginine (R67) and activation-loop threonine (T180) appear to perform roles analogous to those proposed for R69 and T184 in CpCDPK2. In a crystal structure of p38*α *[PDB:3NNX], R67 (equivalent to R69 in CpCDPK2) hydrogen bonds with the glycine backbone of the DFG motif at a distance of 2.8Å, in a manner analogous to CpCDPK2. Another structure of p38*α *complexed with a different inhibitor [PDB:3NNV] shows a similar interaction occurring at 3.2Å. In a structure of mouse p38*α *[PDB:3PY3], phosphorylated on both a threonine (T180) and a tyrosine (T182) in the activation loop, the *α*C arginine (R67) coordinates with the phospho-threonine (Additional File 8). Thus the conserved arginine functions as a switch: upon phosphorylation, the activation-loop phospho-threonine interacts with the *α*C arginine, promoting inter-domain closure and stabilizing the *α*C helix in an active conformation [77]. An equivalent mechanism has been described for p38*γ *[PDB:1CM8] as well [78].

The phosphorylated threonine in p38 corresponds to the TxY motif which is conserved across MAPKs [56], including JNK and ERK1. A sequence alignment of CpCDPK2 and PfCDPK5 along with human p38, JNK1 and ERK1 (Additional File 9) shows that the CDPK subfamily-conserved threonine is centered on the MAPK TxY motif. Another threonine, located 4 residues C-terminal to this site, is broadly conserved in both MAPK and CDPK.

We draw parallels between the observed conformations of CpCDPK2 and p38. An analogous role for R69 and T184 in CpCDPK2 would suggest a regulatory mechanism wherein phosphorylation of T184 leads to kinase activation by repositioning R69 from a DFG-stabilizing or solvent-exposed orientation toward the activation loop, consequently moving the regulatory *α*C helix in an active conformation.

In a paralogous *C. parvum *CDPK that does not belong to the CpCDPK2 subfamily, CDPK1 [PDB:3DFA, EupathDB:cgd3_920], the *α*C arginine is replaced by T50, and the activation loop threonine by D165 (Figure 4A). Rather, the interactions described here are distinctive of the alveolate-specific subfamily of CDPKs including CpCDPK2. The minor expansion of the CDPK family in chromalveolates has created an evolutionary opportunity for certain copies of CDPK genes to subfunctionalize, adapting the additional regulatory role for promoting phosphorylation-dependent inter-domain closure.

### Lineage-specific mechanisms of substrate recognition and binding in CLK

Within the CLK family, we again find a residue pattern that distinguishes chromalveolate CLKs from those in all other eukaryotic lineages. This pattern appears in all apicomplexans surveyed, as well as several dinoflagellates, ciliates, diatoms, and the brown alga *Ectocarpus siliculosus *(Additional File 10). The phyletic distribution of this set of co-conserved motifs points to an origin near the base of Chromalveolata, prior to the emergence of alveolates, and a deep evolutionary divergence between chromalveolates and metazoans.

**Figure 5:**
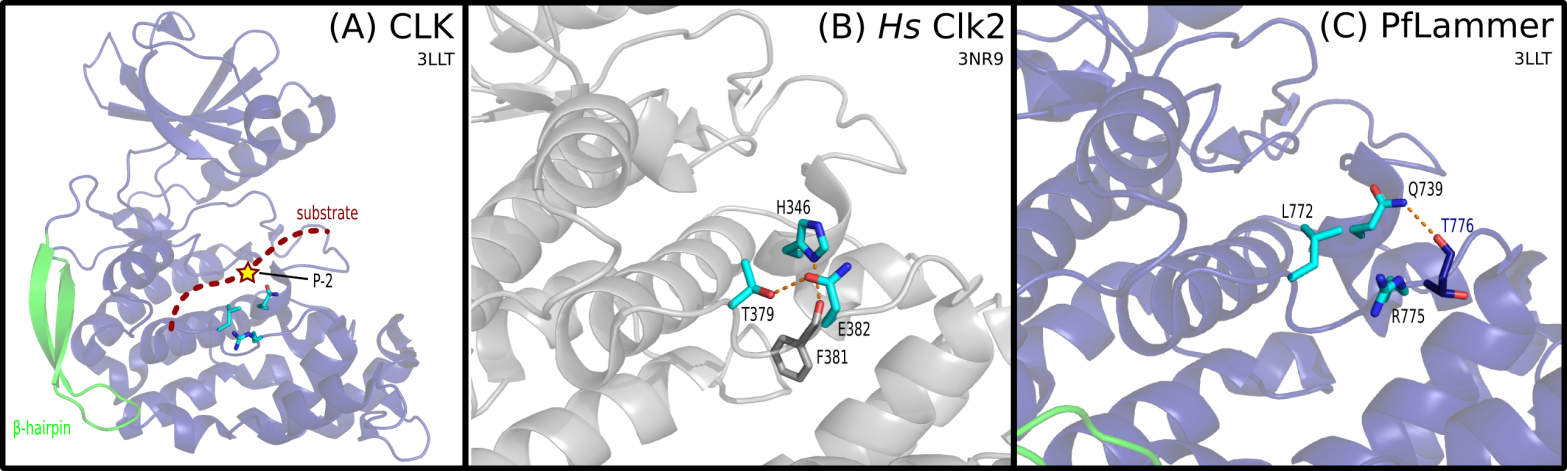
**CLK docking site**. Three contrastingly conserved residues involved in substrate recognition and docking in human Clk2 [PDB:3NR9] and the *P. falciparum *CLK, PfLAMMER [PDB:3LLT]. **(A) **Global view of the docking site, illustrating the position of the substrate RS domain and phosphorylation site. The contrastingly conserved resides are shown in cyan. **(B) **Human Clk2. A trio of constrastingly conserved residues (cyan), along with a nearby phenylalanine (gray), form a network of hydrogen bonds. The conserved histidine (H346) is positioned to interact with the substrate P-2 position. **(C) **In PfLAMMER, the three residues (cyan) are conserved as different types. A glutamine (Q739) replaces the histidine in human Clk2 seen to interact with the substrate P-2 position. The hydrogen bonding network is different: A leucine (L772) replaces the threonine seen in Clk2; an arginine (R775), corresponding to a glutamate in Clk2, is directed away from the other two conserved residues; and the glutamine (Q739) instead forms a hydrogen bond with a nearby threonine.

**Figure 6:**
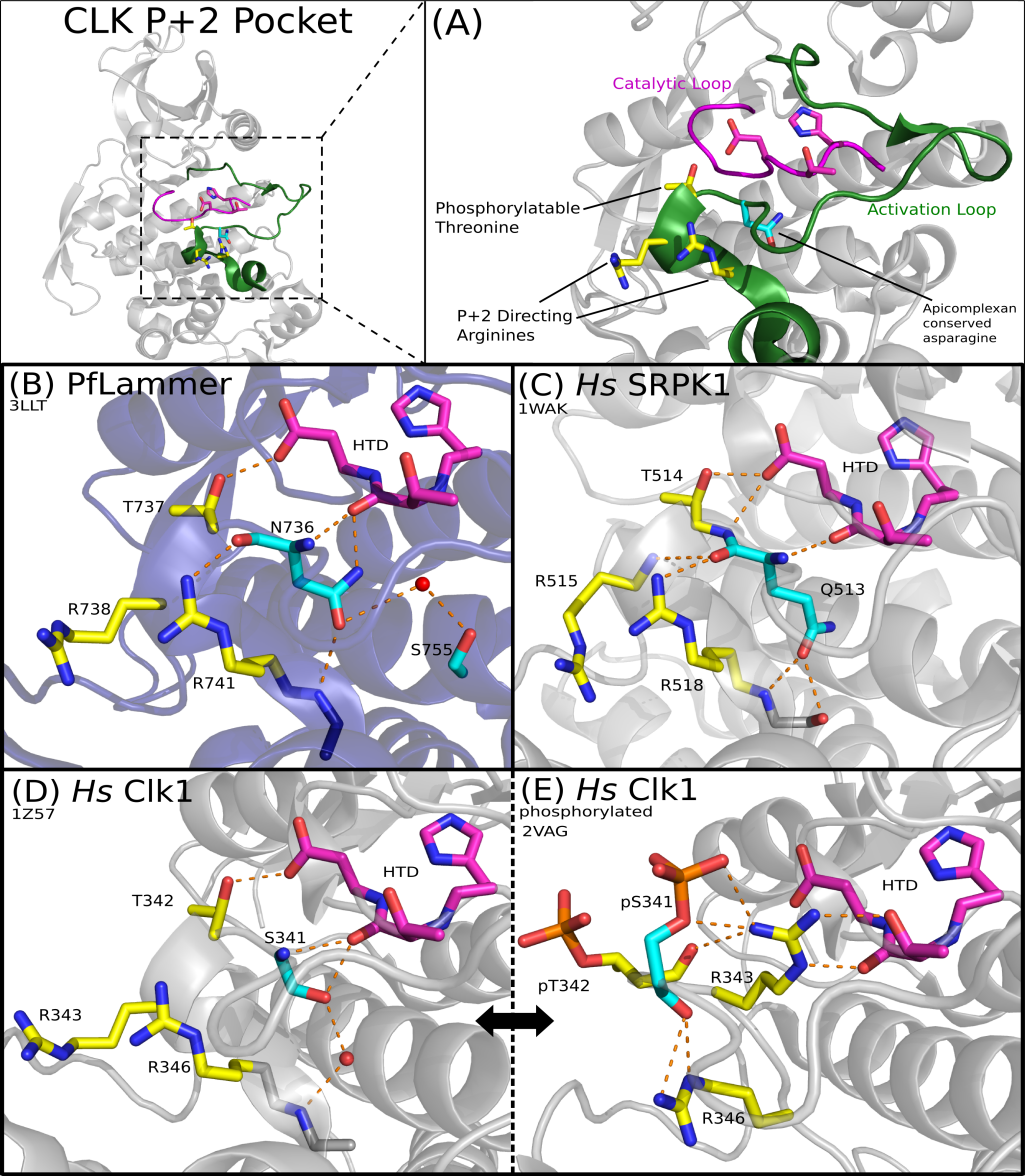
**CLK coordination of substrate-binding and catalytic regions**. Interactions between key residues in the substrate-binding region and the catalytic HTD motif are mediated by conserved residues in the activation loop. **(A) **Structural context of features in PfLAMMER [PDB:3LLT], showing the activation loop in green and the catalytic loop in magenta. Conserved residues are displayed in "sticks" representation. A contrastingly conserved asparagine, distinctive of chromalveolate CLKs, is indicated in cyan, and three other residues conserved throughout the CLK family are shown in yellow. **(B) **In PfLAMMER, the distinctive asparagine (N736) forms hydrogen bonds with the CMGC-conserved arginine (R741), the backbone of the alanine in the APE motif, the backbone of the threonine in the catalytic HTD motif, and, mediated by a water molecule, a subfamily-conserved serine in the *α*F helix. **(C) **In human SRPK1, several of the hydrogen bonds formed by the glutamine Q513 are analogous to those formed by the N736 in apicomplexans. **(D) **and **(E) **Two structures of human Clk1. In the unphosphorylated structure [PDB:1Z57], left, the serine corresponding to PfLAMMER N736 (S341) and the adjacent CLK-conserved threonine (T342) are oriented in an "in" conformation, interacting with the catalytic motif (HTD) but not with the conserved arginines (R343, R346). In the phosphorylated structure [PDB:2VAG], right, the serine (pS341) and threonine (pT342) are flipped to an "out" conformation, breaking the interaction with the catalytic motif. One arginine (R343) moves to occupy the area vacated by the phosphorylated serine S341, while the other (R346) now interacts with the backbone of the phosphorylated serine. Phosphates are shown in orange. Images of PDB structures were rendered using PyMOL [69].

**Figure 7:**
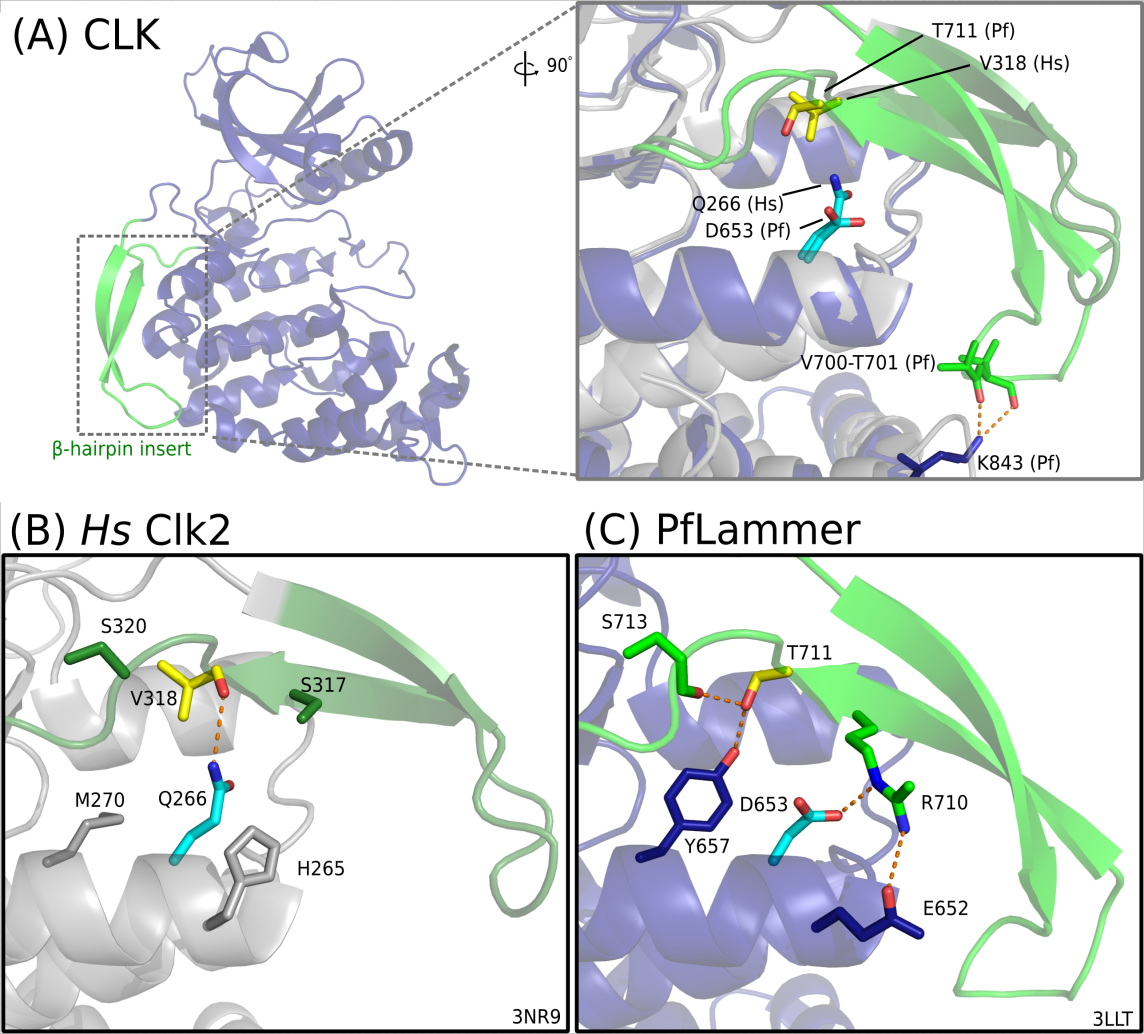
**CLK *β*7-*β*8 hairpin insert and anchoring residues**. Comparison of the residue interactions anchoring the *β*-hairpin insert to the kinase C-lobe in solved structures of PfLAMMER [PDB:3LLT] and human Clk2 [PDB:3NR9]. **(A) **Both structures superimposed, with corresponding key residues shown in "sticks" representation. The contrastingly conserved residue (from CHAIN analysis) is highlighted cyan: D653 in PfLAMMER, Q266 in human Clk2. A residue of interest near the base of the hairpin insert, discussed in the text, is shown in yellow; its type is not strongly conserved within apicomplexan CLKs. Two residues in the loop of the hairpin, colored green, are inserts in PfLAMMER relative to Clk2; they appear anchored to the kinase C-lobe by interactions with a lysine, dark blue. **(B) **Human Clk2, showing side chains near the residues of interest. A hydrogen bond appears between the *α*E-helix glutamine (cyan) and the backbone of a valine (yellow) near the base of the hairpin insert. **(C) **In PfLAMMER, the two residues of interest, D653 (cyan) and T711 (yellow), do not interact directly; each instead forms several novel hydrogen bonds with other nearby residues, shown in green and blue, corresponding to those shown in green and gray in the human Clk2 structure.

These chromalveolate CLKs are distinguished most prominently by residues in the substrate-recognition and docking sites (Additional File 11). Numbered according to the representative *P. falciparum *protein serine/threonine kinase 1 [EupathDB:PF14_0431], also called PfLAMMER [79], the distinguishing residues include Q739, L772 and R775 in the primary docking site, N736 and S755 in the secondary substrate-recognition site, and the acidic residue D653 in the *α*E helix (Figures 5, 6 and 7; discussed below). Taken together, this set of amino acid differences represents a statistically significant partition between chromalveolate and other eukaryotic CLK sequences.

A crystallographic structure of PfLAMMER is available [PDB:3LLT], but has not been previously discussed in detail. We compared this structure to two human CLK homologs, Clk1 [PDB:1Z57, PDB:2VAG] and Clk2 [PDB:3NR9], as well as human SRPK1 [PDB:1WAK], to predict structural and functional roles of the lineage-specific residues. A PyMOL script to visualize the structures of PfLAMMER, Clk2 and Clk3 is provided as Additional File 12.

#### Mechanisms of substrate recognition, binding and processive phosphorylation

The typical substrate of CLK is an SR protein, characterized by an N-terminal RNA-binding domain and an unstructured C-terminal tail of varying length, called the RS domain, which is enriched in arginine and serine, often occurring as "RS" dipeptide repeats [45]. The SR proteins in a cell play multiple roles in spliceosome formation and mRNA splicing activity, including regulation of alternative splicing [80,81]. CLKs are closely related to SRPKs, which also phosphorylate the RS domain of SR proteins. Both kinases are constitutively active, and perform processive phosphorylation on the RS domain of an SR protein substrate, proceeding in the carbonyl-to-amino direction along the substrate peptide [82]. However, differences in substrate binding and the extent of RS domain phosphorylation between SRPK and CLK allow interplay between these proteins to affect the activity and subcellular localization of the SR protein in a complementary fashion [83]. Thus, the complementary regulation of SR proteins by CLK and SRPK has an important functional impact on mRNA splicing in the cell [49].

##### Substrate-recognition site

Three residues responsible for initial recognition of the substrate, Q739, L772 and R775, are contrastingly conserved within the chromalveolate clade (Figure 5). In human Clk2, the equivalent residues H346, T379 and E382 form the substrate-recognition site, with the histidine interacting with the substrate P - 2 residue (P indicates the phosphorylatable residue), preferentially selecting for glutamate [47]. In PfLAMMER the histidine is replaced by a glutamine; the change in chemical properties suggests a different substrate preference for the protein. Additionally, in human Clk2 the three conserved residues form hydrogen bonds with each other and with a nearby F381 (Figure 5B); in PfLAMMER, Q739 only potentially forms a hydrogen bond with nearby residue T776, while L772 appears in place of human T379, losing the bond (Figure 5C). The E382 in Clk2 is replaced in PfLAMMER by R775, which does not form hydrogen bonds with the nearby trio of substrate-recognition residues but is instead oriented outward, free to interact with other atoms, such as the substrate (Figure 5C). The location of the residues L772 and R775 in the loop connecting the *α*F and *α*G helices, in particular, is also significant because the *α*F-*α*G loop is also involved in substrate binding; it is therefore likely that the chromalveolate-specific variations observed in this loop also contribute to a difference in substrate recognition.

##### P+1 binding pocket

As mentioned above, apicomplexan CLKs have conserved lineage-specific residues located at the substrate-binding pocket. One such residue is a the chromalveolate-specific asparagine (N736) in the P+1 pocket. N736 is conserved as a glutamine in SRPKs, as a serine in human Clk1 and Clk2, as a cysteine in GSK, and as a valine in CDK [48]. These variations may contribute to the substrate specificity by subtly altering the geometry of the P+1 pocket. Alternatively, the variation observed at the P+1 pocket may reflect the unique mode of allosteric coupling between the substrate-binding site and active site in CMGC kinases. Notably, both the backbone and side-chain of N736 in PfLAMMER are involved in hydrogen bonding to the backbone of the catalytically important HTD motif (Figure 6B), while in other CMGC kinases, the coupling between the P+1 pocket and catalytic site is largely mediated through backbone hydrogen bonds (Figure 6C,D).

We used the program Coot [84] to examine N736 in the structure of PfLAMMER and found that its backbone conformation lies in a disallowed region of the Ramachandran plot, indicating that torsion-angle strain occurs here. This position has been reported to be in a strained position in SRPK1 and other CMGCs prior to substrate binding; substrate binding relieves this strain, highlighting the importance of this residue in the substrate binding mechanism [48]. It is also significant that in one of the human Clk1 structures [PDB:2VAG] (Figure 6E), S341 (equivalent to N736 in PfLAMMER) and T342 are phosphorylated, which dramatically alters the geometry of the P+1 pocket and inactivates the kinase [85]. This indicates that the P+1 pocket is conformationally malleable and can contribute to the unique modes of allosteric regulation.

##### Proline-directed and processive phosphorylation

The CLK family, and related members of the CMGC group, conserve several distinctive residues in the substrate-binding site that contribute to the substrate specificity of CMGC kinases. One such residue is the distinctive CMGC-arginine [48] (R741 in Figure 6B) located at the C-terminal end of the activation loop. The CMGC-arginine contributes to substrate specificity by creating a favorable hydrophobic environment for a proline at the P+1 position of the substrate. Specifically, the CMGC-arginine caps the backbone carbonyl oxygen of a residue (N736 in PfLAMMER) in the P+1 pocket that typically hydrogen bonds to the backbone amide of a residue at the P+1 position. Because proline lacks a backbone amide, the capping of carbonyl oxygen by the CMGC-arginine allows selective binding of substrates with proline at the P+1 position [72]. The presence of the CMGC-arginine and the hydrogen bonds in the P+1 pocket of PfLAMMER (Figure 6C) suggest that chromalveolate CLKs, like other CMGC kinases [45,86], are likely to be proline-directed.

PfLAMMER also conserves the P - 2 arginine (R738 in Figure 6C), which in human CLKs and SRPKs contributes to the processive phosphorylation of substrates by stabilizing a phosphorylated serine or threonine at the P - 2 position in the substrate [47,48]. This feature suggests that chromalveolate CLKs, like human and plant CLKs and SRPKs, may processively phosphorylate substrates with phosphorylatable serine or threonine at the P - 2 position. Indeed, a search for protein sequences with an RNA-binding domain [Pfam:RRM_1] and "RS" repeat regions identified at least three possible SR proteins in *P. falciparum *[EupathDB:PF10_0217, PFE0865c, PFE0160c], each with orthologs in other apicomplexan species [OrthoMCL:OG5_127971, OG5_128933, OG5_127418].

#### Chromalveolate-specific features in the distal substrate-recognition site

The CLK family, as it appears in all eukaryotes including apicomplexans, has a characteristic *β*-hairpin insert in the C-lobe between the *β*7 and *β*8 strands, which blocks its SR protein substrate from docking in what is a distal substrate-recognition groove in other CMGCs (such as the MAP kinase p38) [47]. Blocking this docking interaction is critical for CLK substrate specificity, the primary means by which CLKs are regulated [47].

CHAIN analysis revealed a strikingly conserved acidic residue (aspartate or glutamate) in the *α*E helix of chromalveolate CLKs which in other eukaryotic CLKs is generally a histidine or a glutamine. This difference is reflected in the anchoring of the *β*-hairpin insert to the C-lobe of the kinase domain (Figure 7). In PfLAMMER, the conserved acidic residue is D653; the equivalent residue in human Clk2 [PDB:3NR9] is Q266. In Clk2, the MAPK substrate-recognition groove is occupied by a hydrophobic V318; Q266 stabilizes the backbone of V318 in human Clk2 (Figure 7B). In contrast, the distinctive D653 in PfLAMMER participates in a network of hydrogen bonds involving an arginine in the *β*-hairpin insert; the V318 in Clk2 is replaced by T711, which itself forms hydrogen bonds with two other residues in the *α*E helix and at the base of the insert, rather than with D653 (Figure 7C). Together these changes appear to further stabilize the beta-hairpin insert in *P. falciparum *by forming additional interactions. The changes also make the pocket more hydrophilic relative to Clk2.

The *β*-hairpin insert is several residues longer in chromalveolate CLKs than in human Clk2. In the PfLAMMER structure [PDB:3LLT], the hairpin loop is also anchored to the kinase C-lobe by a hydrogen bond between a lysine (K843) in the C-lobe and the backbone of the hairpin loop — this lysine, and consequently the hydrogen bond, is not seen in human Clk2 (Figure 7A). However, it is also possible that the interaction occurs in the solved structure as a consequence of crystal packing, in which case there may be no functional significance *in vivo*.

These variations, along with the variations in the primary substrate-binding site, indicate that apicomplexan and other chromalveolate CLKs have diverged from their human counterparts and specifically recognize and phosphorylate selected protein substrates.

## Conclusions

We have used an approach based on evolutionary analysis to identify statistically distinct subfamilies of CDK and CDPK in the Apicomplexa and Chromalveolata, and explore the structural adaptations of CLK for substrate binding among chromalveolates. We discussed the functional implications of these distinguishing variations, confirmed and clarified previously published results regarding protein kinases in apicomplexan species, and proposed a set of new testable functional hypotheses, which we hope will focus future experimental efforts.

This methodology has provided a means for identifying clade-specific sequence and structural features which may be associated with functional specialization. We presented three well-supported lineage-specific groups of kinases that emerged from our analysis, supported by existing structural and functional data about related proteins, and inferred additional functional hypotheses and the mechanisms that might enable these functions. Two of these sub-groups are members of the CMGC kinase group, which is highly conserved across Eukaryota, allowing strong homologies to be drawn between extant species to reveal ancient divergences along evolutionary branches. The third family, CDPK, is largely specific to plastid-containing eukaryotes in the Chromalveolata and Viridiplantae (but also found in other protozoans), but is also relatively more highly duplicated in each genome; the additional gene copies enhanced the statistical support for a proposed subfamily. The public availability of whole-genome sequences from diverse apicomplexan species likewise enabled the detection of deeply conserved sequence patterns. The work of the Structural Genomics Consortium [87] has also been invaluable in providing structural evidence for this neglected branch of protozoa.

Not every eukaryotic protein kinase family in apicomplexans yielded a distinctive feature set, however. Many of the "Other" kinase families are difficult to classify precisely; some are lineage-specific, and some have a mix of sequence features shared by multiple kinase families — the PfPK7 family, in fact, presents both problems [18]. The previously identified apicomplexan-specific families, FIKK and ROPK, are not strong candidates for CHAIN analysis, either: Since all of the species containing these families belong to the same phylum, shared sequence features within a sub-clade are likely to be the result of recent common ancestry rather than functional constraints on their molecular evolution. Despite these limitations, the approach we have presented will be useful for further analysis of apicomplexans as additional whole-genome sequences and protein kinase structures become available.

In the search for potential therapeutic targets for parasitic diseases, identification of these features and the molecular mechanisms they represent could lead to potential candidates for selective targeting. The taxonomic distribution of these novel protein features also provides insight into the evolution of apicomplexans and chromalveolates, lending support to the current understanding of these species' history.

## Methods

### Genome data sources

The protein complements of 17 complete genomes, from 15 distinct apicomplexan species, were retrieved from EupathDB [88]. The genomes of three non-apicomplexan species were also obtained for comparison (Table 2).

**Table 2:** Genome sources

Genomes	Source
*Plasmodium berghei *ANKA, *P. chabaudi *AS, *P. falciparum *3D7, *P. knowlesi *H, *P. vivax *Salvador I, *P. yoelii *17XNL	PlasmoDB v.8.0 [6-10,96]
*Babesia bovis *T2Bo; *Theileria annulata *Ankara, *T. parva *Mugaga	PiroplasmaDB v.1.1 [11-13]
*Neospora caninum*; *Toxoplasma gondii *GT1, ME49, VEG; *Eimeria tenella *Houghton	ToxoDB v.7.0 [97]
*Cryptosporidium hominis*, *C. muris*, *C. parvum *Iowa II	CryptoDB v.4.5 [14-16]
*Perkinsus marinus *ATCC 50983	NCBI genome project 12737
*Thalassiosira pseudonana *CCMP1335	NCBI genome project 34119 [98,99]
*Saccharomyces cerevisiae*	Kinbase (http://kinase.com/kinbase/), Saccharomyces Genome Database (http://yeastgenome.org/)

To obtain a sequence set of all solved apicomplexan ePK structures, the August 2011 release of PDBAA, the protein sequence database derived from PDB, was downloaded from NCBI. Phylum labels were added to the sequence headers according to GI number using the NCBI taxonomy data set, and sequences from the phylum Apicomplexa were selected.

### Identification, classification and alignment of eukaryotic protein kinases (ePKs) in selected genomes

We constructed a curated set of ePK family profiles using previously annotated sequences from diverse model organisms. The classification scheme is based on the kinase groups and families described in previous kinomic analyses [27,28,89]. Additional profiles for the FIKK, ROPK and PfPK7 families were built from apicomplexan sequences with annotations supported by experimental studies in published literature [21,24].

We used the MAPGAPS program with the curated profile sets to identify, classify and align the protein kinases in the genomic sequences, as well as the apicomplexan ePK structures in PDB. MAPGAPS selects all sequences with a kinase domain containing key motifs, assigns each sequence with a significant hit to the best-matching family in the query profile, and accurately aligns each hit to the kinase consensus sequence, capturing conserved motifs [90]. Fragmentary sequences were then deleted.

Identification and classification of the ePKs in each genome revealed certain families present in multiple copies, providing enough data for further comparative analysis. The sequence counts in this scan generally agree with previously published kinome analyses, though because these and most previous annotations are produced by different computational methods there is occasional disagreement over the classification of more divergent sequences lacking clear orthologs in model organisms.

### Gene tree inference to find divergent apicomplexan ortholog groups

Within each assigned ePK family, we concatenated the three sequence sets (apicomplexan genomic; a profile of sequences from model organisms including human; apicomplexan PDB sequences) and realigned the kinase domains using MAPGAPS to prepare a sequence alignment for phylogenetic analysis. To infer a gene tree from each of these alignments, we used RAxML with the fast bootstrap and maximum likelihood tree estimation procedure [91], PROTGAMMAWAG model (WAG amino acid substitution model with the rate heterogeneity), and 500 bootstrap replicates. We then used a custom script based on Biopython [70] to collapse branches with less than 50% bootstrap support in the resulting gene trees.

A resolved clade in the gene tree containing sequences from a monophyletic group of species, in agreement with the established species tree, indicates that the genes are orthologous. We selected clades that contained sequences from several apicomplexan species, but did not include any metazoan sequences, and with particular interest in clades containing PDB structures, for further analysis.

### Patterns of functional divergence

We queried related families of diverse sequences with selected clusters using the CHAIN program [33]. For each apicomplexan-specific cluster, we used the sequences from each gene clade of interest (described above) as the query set, and the sequences of diverse eukaryotic species in the corresponding kinase family as the main set, constructed from all kinase family members found in NCBI-nr. Both the query and main sequence sets were aligned with MAPGAPS for comparison.

The Bayesian Pattern Partitioning Search (BPPS) procedure in CHAIN simultaneously identifies selective constraints imposed on the foreground sequences, and pulls any sequences from the background that share the identified patterns in the query into the foreground, precisely defining a statistically supported family or subfamily if one exists [33].

## Authors' contributions

NK designed and conceived the project. ET performed the bioinformatics analyses. ET, AM and NK examined sequences and structural features and wrote the manuscript. All authors read and approved the final manuscript.

## Supplementary Material

Additional file 1 **Kinome annotations**. Zip archive of hierarchical kinase classifications for each gene in the kinomes of each apicomplexan, plus *P. marinus *and *T. pseudonana*. Each file contains two tab-separated columns listing each gene's accession and kinase family assignment. Accessions are taken from the sources listed in Table 2. Click here for file

Additional file 2 **CMGC kinase family sizes**. Number of copies of each conserved CMGC kinase family in each of the surveyed genomes. Click here for file

Additional file 3 **CDK-SCTTLRE subfamily FASTA alignment**. Plain-text alignment of the kinase domains of the 14 sequences belonging to proposed CDK subfamily ("SCTTLRE"), in FASTA format. Click here for file

Additional file 4 **CDK-SCTTLRE subfamily CHAIN alignment versus the CDK family**. Colorized sequence alignment and partition generated by the CHAIN program, comparing the apicomplexan-specific subfamily of CDKs to a diverse set of eukaryotic CDKs. CHAIN compares a given "query" set (here, members of the putative subfamily) to a larger "main" set (here, a diverse set of eukaryotic CDKs) and divides the main set into 3 partitions based on contrasting levels of residue conservation: a "foreground" set of sequences with residue motifs matching the query, a "background" which does not conserve the distinguishing motifs of the foreground, and an "intermediate" which contains sequences that may partially match both the foreground and background sequence motifs. The alignment summary generated by CHAIN displays only the aligned sequences in the query, but highlights the alignment columns according to the conservation patterns defining each partition. The alignment appears as three blocks, labeled "Intermediate", "Background" and "Foreground", corresponding to those partitions. Above each block is a histogram indicating residue conservation patterns unique to that sequence set; dots above each column indicate which columns form the distinguishing pattern. Thus, tall red bars above columns in the "Foreground" block indicate residues that are strikingly conserved in the foreground, but not in the background. The rows below each "Background" and "Foreground" block indicate the conserved residue types and their conservation levels within those sequence sets, in units of 10%. Click here for file

Additional file 5 **CDK-SCTTLRE subfamily CHAIN alignment versus the CDC2 subfamily**. Colorized sequence alignment and partition generated by the CHAIN program, comparing the apicomplexan-specific subfamily of CDKs to eukaryotic CDC2 subfamily members. Click here for file

Additional file 6 **CDPK subfamily FASTA alignment**. Plain-text alignment of the 76 kinase domain sequences belonging to the proposed CDPK subfamily, in FASTA format. Click here for file

Additional file 7 **CDPK subfamily CHAIN alignment**. Colorized sequence alignment and partition generated by the CHAIN program, comparing the lineage-specific subfamily of CDPKs to a large set of chromalveolate CDPKs. Click here for file

Additional file 8 **CpCDPK2 and MAPK structure alignment for PyMOL**. PyMOL script to superimpose structures of CpCDPK2 and phosphorylated mammalian p38*α*, a MAP kinase. The structures are automatically downloaded from the wwPDB server and aligned within PyMOL. Constrastingly conserved CpCDPK2 residues identified by CHAIN, and the equivalents in p38*α*, are highlighted as sticks. The reader is encouraged to explore nearby side chains and other features using the built-in capabilities of PyMOL. Click here for file

Additional file 9 **Alignment of selected CDPK subfamily and MAPK sequences**. Annotated alignment of CDPK subfamily representatives CpCDPK2 and PfCDPK5 with human MAPK sequences p38, JNK1 and ERK1. GUIDANCE [100] was used to align the sequence segments, calculate reliability scores, and generate the initial version of the figure, to which we added further annotations. Click here for file

Additional file 10 **CLK family FASTA alignment**. Plain-text alignment of the kinase domains of 33 sequences belonging to a divergent clade of CLK, in FASTA format. Click here for file

Additional file 11 **CLK family CHAIN alignment**. Colorized sequence alignment and partition generated by the CHAIN program, comparing the apicomplexan-specific subfamily of CLKs to a diverse set of eukaryotic CLKs. Click here for file

Additional file 12 **CLK family structure alignment for PyMOL**. PyMOL script to superimpose structures of PfLAMMER and human Clk2 and Clk3. Constrastingly conserved PfLAMMER residues identified by CHAIN, and the equivalents in the human CLKs, are highlighted as sticks. Click here for file

## References

[B1] Roos DS Genetics. Themes and variations in apicomplexan parasite biology Science 2005 309 5731 72 3 10.1126/science.1115252 15994520

[B2] Renslo AR McKerrow JH Drug discovery and development for neglected parasitic diseases Nature chemical biology 2006 2 12 701 10 10.1038/nchembio837 17108988

[B3] Sibley LD Intracellular parasite invasion strategies Science 2004 304 5668 248 53 10.1126/science.1094717 15073368

[B4] Hammarton T The cell cycle of parasitic protozoa: potential for chemotherapeutic exploitation Progress In Cell Cycle Research 2003 5 91 101 14593704

[B5] Doerig C Abdi A Bland N Eschenlauer S Dorin-Semblat D Fennell C Halbert J Holland Z Nivez MP Semblat JP Sicard A Reininger L Malaria: targeting parasite and host cell kinomes Biochimica et biophysica acta 2010 1804 3 604 12 10.1016/j.bbapap.2009.10.009 19840874

[B6] Hall N Karras M Raine JD Carlton JM Kooij TWa Berriman M Florens L Janssen CS Pain A Christophides GK James K Rutherford K Harris B Harris D Churcher C Quail Ma Ormond D Doggett J Trueman HE Mendoza J Bidwell SL Rajandream MA Carucci DJ Yates JR Kafatos FC Janse CJ Barrell B Turner CMR Waters AP Sinden RE A comprehensive survey of the Plasmodium life cycle by genomic, transcriptomic, and proteomic analyses Science 2005 307 5706 82 6 10.1126/science.1103717 15637271

[B7] Gardner MJ Hall N Fung E White O Berriman M Hyman RW Carlton JM Pain A Nelson KE Bowman S Paulsen IT James K Eisen Ja Rutherford K Salzberg SL Craig A Kyes S Chan MS Nene V Shallom SJ Suh B Peterson J Angiuoli S Pertea M Allen J Selengut J Haft D Mather MW Vaidya AB Martin DMa Fairlamb AH Fraunholz MJ Roos DS Ralph Sa McFadden GI Cummings LM Subramanian GM Mungall C Venter JC Carucci DJ Hoffman SL Newbold C Davis RW Fraser CM Barrell B Genome sequence of the human malaria parasite Plasmodium falciparum Nature 2002 419 6906 498 511 10.1038/nature01097 PMC3836256 12368864

[B8] Pain a Böhme U Berry aE Mungall K Finn RD Jackson aP Mourier T Mistry J Pasini EM Aslett Ma Balasubrammaniam S Borgwardt K Brooks K Carret C Carver TJ Cherevach I Chillingworth T Clark TG Galinski MR Hall N Harper D Harris D Hauser H Ivens A Janssen CS Keane T Larke N Lapp S Marti M Moule S Meyer IM Ormond D Peters N Sanders M Sanders S Sargeant TJ Simmonds M Smith F Squares R Thurston S Tivey aR Walker D White B Zuiderwijk E Churcher C Quail Ma Cowman aF Turner CMR Rajandream Ma Kocken CHM Thomas aW Newbold CI Barrell BG Berriman M The genome of the simian and human malaria parasite Plasmodium knowlesi Nature 2008 455 7214 799 803 10.1038/nature07306 PMC2656934 18843368

[B9] Carlton JM Adams JH Silva JC Bidwell SL Lorenzi H Caler E Crabtree J Angiuoli SV Merino EF Amedeo P Cheng Q Coulson RMR Crabb BS Del Portillo Ha Essien K Feldblyum TV Fernandez-Becerra C Gilson PR Gueye AH Guo X Kang'a S Kooij TWa Korsinczky M Meyer EVS Nene V Paulsen I White O Ralph Sa Ren Q Sargeant TJ Salzberg SL Stoeckert CJ Sullivan Sa Yamamoto MM Hoffman SL Wortman JR Gardner MJ Galinski MR Barnwell JW Fraser-Liggett CM Comparative genomics of the neglected human malaria parasite Plasmodium vivax Nature 2008 455 7214 757 63 10.1038/nature07327 PMC2651158 18843361

[B10] Carlton JM Angiuoli SV Suh BB Kooij TW Pertea M Silva JC Ermolaeva MD Allen JE Selengut JD Koo HL Peterson JD Pop M Kosack DS Shumway MF Bidwell SL Shallom SJ van Aken SE Riedmuller SB Feldblyum TV Cho JK Quackenbush J Sedegah M Shoaibi A Cummings LM Florens L Yates JR Raine JD Sinden RE Harris Ma Cunningham Da Preiser PR Bergman LW Vaidya AB van Lin LH Janse CJ Waters AP Smith HO White OR Salzberg SL Venter JC Fraser CM Hoffman SL Gardner MJ Carucci DJ Genome sequence and comparative analysis of the model rodent malaria parasite Plasmodium yoelii yoelii Nature 2002 419 6906 512 9 10.1038/nature01099 12368865

[B11] Brayton Ka Lau AOT Herndon DR Hannick L Kappmeyer LS Berens SJ Bidwell SL Brown WC Crabtree J Fadrosh D Feldblum T Forberger HA Haas BJ Howell JM Khouri H Koo H Mann DJ Norimine J Paulsen IT Radune D Ren Q Smith RK Suarez CE White O Wortman JR Knowles DP McElwain TF Nene VM Genome sequence of Babesia bovis and comparative analysis of apicomplexan hemoprotozoa PLoS pathogens 2007 3 10 1401 13 10.1371/journal.ppat.0030148 PMC2034396 17953480

[B12] Pain A Renauld H Berriman M Murphy L Yeats Ca Weir W Kerhornou A Aslett M Bishop R Bouchier C Cochet M Coulson RMR Cronin A de Villiers EP Fraser A Fosker N Gardner M Goble A Griffths-Jones S Harris DE Katzer F Larke N Lord A Maser P McKellar S Mooney P Morton F Nene V O'Neil S Price C Quail Ma Rabbinowitsch E Rawlings ND Rutter S Saunders D Seeger K Shah T Squares R Squares S Tivey A Walker AR Woodward J Dobbelaere DaE Langsley G Rajandream MA McKeever D Shiels B Tait A Barrell B Hall N Genome of the host-cell transforming parasite Theileria annulata compared with T. parva Science 2005 309 5731 131 3 10.1126/science.1110418 15994557

[B13] Gardner MJ Bishop R Shah T de Villiers EP Carlton JM Hall N Ren Q Paulsen IT Pain A Berriman M Wilson RJM Sato S Ralph Sa Mann DJ Xiong Z Shallom SJ Weidman J Jiang L Lynn J Weaver B Shoaibi A Domingo AR Wasawo D Crabtree J Wortman JR Haas B Angiuoli SV Creasy TH Lu C Suh B Silva JC Utterback TR Feldblyum TV Pertea M Allen J Nierman WC Taracha ELN Salzberg SL White OR Fitzhugh Ha Morzaria S Venter JC Fraser CM Nene V Genome sequence of Theileria parva, a bovine pathogen that transforms lymphocytes Science 2005 309 5731 134 7 10.1126/science.1110439 15994558

[B14] Heiges M Wang H Robinson E Aurrecoechea C Gao X Kaluskar N Rhodes P Wang S He CZ Su Y Miller J Kraemer E Kissinger JC CryptoDB: a Cryptosporidium bioinformatics resource update Nucleic acids research 2006 34 Database D419 22 10.1093/nar/gkj078 PMC1347441 16381902

[B15] Xu P Widmer G Wang Y Ozaki LS Alves JM Serrano MG Puiu D Manque P Akiyoshi D Mackey AJ Pearson WR Dear PH Bankier AT Peterson DL Abrahamsen MS Kapur V Tzipori S Buck GA The genome of Cryptosporidium hominis Nature 2004 431 7012 1107 12 10.1038/nature02977 15510150

[B16] Abrahamsen MS Templeton TJ Enomoto S Abrahante JE Zhu G Lancto Ca Deng M Liu C Widmer G Tzipori S Buck Ga Xu P Bankier AT Dear PH Konfortov Ba Spriggs HF Iyer L Anantharaman V Aravind L Kapur V Complete genome sequence of the apicomplexan, Cryptosporidium parvum Science 2004 304 5669 441 5 10.1126/science.1094786 15044751

[B17] Bontell IL Hall N Ashelford KE Dubey JP Boyle JP Lindh J Smith JE Whole genome sequencing of a natural recombinant Toxoplasma gondii strain reveals chromosome sorting and local allelic variants Genome biology 2009 10 5 R53 10.1186/gb-2009-10-5-r53 PMC2718519 19457243

[B18] Dorin D Semblat JP Poullet P Alano P Goldring JPD Whittle C Patterson S Chakrabarti D Doerig C PfPK7, an atypical MEK-related protein kinase, reflects the absence of classical three-component MAPK pathways in the human malaria parasite Plasmodium falciparum Molecular microbiology 2005 55 184 96 10.1111/j.1365-2958.2004.04393.x 15612927

[B19] Lourido S Shuman J Zhang C Shokat KM Hui R Sibley LD Calcium-dependent protein kinase 1 is an essential regulator of exocytosis in Toxoplasma Nature 2010 465 7296 359 62 10.1038/nature09022 PMC2874977 20485436

[B20] Tewari R Straschil U Bateman A Böhme U Cherevach I Gong P Pain A Billker O The systematic functional analysis of Plasmodium protein kinases identifies essential regulators of mosquito transmission Cell host & microbe 2010 8 4 377 87 10.1016/j.chom.2010.09.006 PMC2977076 20951971

[B21] Ward P Equinet L Packer J Doerig C Protein kinases of the human malaria parasite Plasmodium falciparum: the kinome of a divergent eukaryote BMC Genomics 2004 5 79 10.1186/1471-2164-5-79 PMC526369 15479470

[B22] Sargeant TJ Marti M Caler E Carlton JM Simpson K Speed TP Cowman AF Lineage-specific expansion of proteins exported to erythrocytes in malaria parasites Genome biology 2006 7 2 R12 10.1186/gb-2006-7-2-r12 PMC1431722 16507167

[B23] Schneider AG Mercereau-Puijalon O A new Apicomplexa-specific protein kinase family: multiple members in Plasmodium falciparum, all with an export signature BMC Genomics 2005 6 30 10.1186/1471-2164-6-30 PMC1079819 15752424

[B24] Peixoto L Chen F Harb OS Davis PH Beiting DP Brownback CS Ouloguem D Roos DS Integrative genomic approaches highlight a family of parasite-specific kinases that regulate host responses Cell host & microbe 2010 8 2 208 18 10.1016/j.chom.2010.07.004 PMC2963626 20709297

[B25] Dorin-Semblat D Sicard A Doerig C Ranford-Cartwright L Doerig C Disruption of the PfPK7 gene impairs schizogony and sporogony in the human malaria parasite Plasmodium falciparum Eukaryotic cell 2008 7 2 279 85 10.1128/EC.00245-07 PMC2238148 18083830

[B26] Merckx A Echalier A Langford K Sicard A Langsley G Joore J Doerig C Noble M Endicott J Structures of P. falciparum protein kinase 7 identify an activation motif and leads for inhibitor design Structure 2008 16 2 228 38 10.1016/j.str.2007.11.014 18275814

[B27] Hanks SK Hunter T The eukaryotic protein kinase superfamily: kinase (catalytic) domain structure and classification FASEB 1995 9 576 596 7768349

[B28] Manning G Whyte DB Martinez R Hunter T Sudarsanam S The protein kinase complement of the human genome Science 2002 298 5600 1912 34 10.1126/science.1075762 12471243

[B29] Nagamune K Sibley LD Comparative genomic and phylogenetic analyses of calcium ATPases and calcium-regulated proteins in the apicomplexa Molecular biology and evolution 2006 23 8 1613 27 10.1093/molbev/msl026 16751258

[B30] Wernimont AK Artz JD Finerty P Lin YH Amani M Allali-Hassani A Senisterra G Vedadi M Tempel W Mackenzie F Chau I Lourido S Sibley LD Hui R Structures of apicomplexan calcium-dependent protein kinases reveal mechanism of activation by calcium Nature structural & molecular biology 2010 17 5 596 601 10.1038/nsmb.1795 PMC3675764 20436473

[B31] Billker O Lourido S Sibley L Calcium-dependent signaling and kinases in apicomplexan parasites Cell host & microbe 2009 5 6 612 622 10.1016/j.chom.2009.05.017 PMC2718762 19527888

[B32] Artz J Wernimont A Allali-Hassani A Zhao Y Amani M Lin YH Senisterra G Wasney G Fedorov O King O Roos A Lunin V Qiu W Finerty P Hutchinson A Chau I von Delft F MacKenzie F Lew J Kozieradzki I Vedadi M Schapira M Zhang C Shokat K Heightman T Hui R The Cryptosporidium parvum Kinome BMC Genomics 2011 12 478 10.1186/1471-2164-12-478 PMC3227725 21962082

[B33] Neuwald AF The CHAIN program: forging evolutionary links to underlying mechanisms Trends in biochemical sciences 2007 32 11 487 93 10.1016/j.tibs.2007.08.009 17962021

[B34] Berman HM Westbrook J Feng Z Gilliland G Bhat TN Weissig H Shindyalov IN Bourne PE The Protein Data Bank Nucleic acids research 2000 28 235 42 10.1093/nar/28.1.235 PMC102472 10592235

[B35] Keeling PJ Burger G Durnford DG Lang BF Lee RW Pearlman RE Roger AJ Gray MW The tree of eukaryotes Trends in ecology & evolution 2005 20 12 670 6 10.1016/j.tree.2005.09.005 16701456

[B36] Adl SM Simpson AGB Farmer MA Andersen RA Anderson OR Barta JR Bowser SS Brugerolle G Fensome RA Fredericq S James TY Karpov S Kugrens P Krug J Lane CE Lewis LA Lodge J Lynn DH Mann DG McCourt RM Mendoza L Moestrup O Mozley-Standridge SE Nerad TA Shearer CA Smirnov AV Spiegel FW Taylor MFJR The new higher level classification of eukaryotes with emphasis on the taxonomy of protists The Journal of eukaryotic microbiology 2005 52 5 399 451 10.1111/j.1550-7408.2005.00053.x 16248873

[B37] Joseph SJ Fernández-Robledo JA Gardner MJ El-Sayed NM Kuo CH Schott EJ Wang H Kissinger JC Vasta GR The Alveolate Perkinsus marinus: biological insights from EST gene discovery BMC Genomics 2010 11 228 10.1186/1471-2164-11-228 PMC2868825 20374649

[B38] Bemm F Schwarz R Förster F Schultz J A kinome of 2600 in the ciliate Paramecium tetraurelia FEBS letters 2009 583 22 3589 92 10.1016/j.febslet.2009.10.029 19840790

[B39] Miranda-Saavedra D Stark MJR Packer JC Vivares CP Doerig C Barton GJ The complement of protein kinases of the microsporidium Encephalitozoon cuniculi in relation to those of Saccharomyces cerevisiae and Schizosaccharomyces pombe BMC Genomics 2007 8 309 10.1186/1471-2164-8-309 PMC2078597 17784954

[B40] Lawrence JG Common themes in the genome strategies of pathogens Current opinion in genetics & development 2005 15 6 584 8 10.1016/j.gde.2005.09.007 16188434

[B41] Templeton TJ Iyer LM Anantharaman V Enomoto S Abrahante JE Subramanian GM Hoffman SL Abrahamsen MS Aravind L Comparative analysis of apicomplexa and genomic diversity in eukaryotes Genome research 2004 14 9 1686 95 10.1101/gr.2615304 PMC515313 15342554

[B42] Kuo CH Kissinger JC Consistent and contrasting properties of lineage-specific genes in the apicomplexan parasites Plasmodium and Theileria BMC Evolutionary Biology 2008 8 108 10.1186/1471-2148-8-108 PMC2330040 18405380

[B43] Martin DMa Miranda-Saavedra D Barton GJ Kinomer v. 1.0: a database of systematically classified eukaryotic protein kinases Nucleic acids research 2009 37 Database D244 50 10.1093/nar/gkn834 PMC2686601 18974176

[B44] Striepen B Jordan CN Reiff S van Dooren GG Building the perfect parasite: cell division in apicomplexa PLoS pathogens 2007 3 6 e78 10.1371/journal.ppat.0030078 PMC1904476 17604449

[B45] Colwill K Feng LL Yeakley JM Gish GD Cáceres JF Pawson T Fu XD SRPK1 and Clk/Sty protein kinases show distinct substrate specificities for serine/arginine-rich splicing factors The Journal of biological chemistry 1996 271 40 24569 75 10.1074/jbc.271.40.24569 8798720

[B46] Kojima T Zama T Wada K Onogi H Hagiwara M Cloning of human PRP4 reveals interaction with Clk1 The Journal of biological chemistry 2001 276 34 32247 56 10.1074/jbc.M103790200 11418604

[B47] Bullock AN Das S Debreczeni JE Rellos P Fedorov O Niesen FH Guo K Papagrigoriou E Amos AL Cho S Turk BE Ghosh G Knapp S Kinase domain insertions define distinct roles of CLK kinases in SR protein phosphorylation Structure (London, England: 1993) 2009 17 3 352 62 10.1016/j.str.2008.12.023 PMC2667211 19278650

[B48] Kannan N Neuwald AF Evolutionary constraints associated with functional specificity of the CMGC protein kinases MAPK, CDK, GSK, SRPK, DYRK, and CK2alpha Protein science: a publication of the Protein Society 2004 13 8 2059 77 10.1110/ps.04637904 PMC2279817 15273306

[B49] Dixit A Singh PK Sharma GP Malhotra P Sharma P PfSRPK1, a novel splicing-related kinase from Plasmodium falciparum The Journal of biological chemistry 2010 285 49 38315 23 10.1074/jbc.M110.119255 PMC2992265 20870716

[B50] Yun B Farkas R Lee K Rabinow L The Doa locus encodes a member of a new protein kinase family and is essential for eye and embryonic development in Drosophila melanogaster Genes & Development 1994 8 10 1160 1173 10.1101/gad.8.10.1160 7926721

[B51] Agarwal S Kern S Halbert J Przyborski JM Baumeister S Dandekar T Doerig C Pradel G Two nucleus-localized CDK-like kinases with crucial roles for malaria parasite erythrocytic replication are involved in phosphorylation of splicing factor Journal of cellular biochemistry 2011 112 5 1295 310 10.1002/jcb.23034 21312235

[B52] Fluhr R Reddy ASN, Golovkin M Regulation of Splicing by Protein Phosphorylation Nuclear pre-mRNA Processing in Plants, Volume 326 of Current Topics in Microbiology and Immunology 2008 Berlin, Heidelberg: Springer Berlin Heidelberg 119 138

[B53] Chen F Mackey AJ Stoeckert CJ Roos DS OrthoMCL-DB: querying a comprehensive multi-species collection of ortholog groups Nucleic acids research 2006 34 Database D363 8 10.1093/nar/gkj123 PMC1347485 16381887

[B54] Wernimont AK Amani M Qiu W Pizarro JC Artz JD Lin YH Lew J Hutchinson A Hui R Structures of parasitic CDPK domains point to a common mechanism of activation Proteins 2010 1 18 10.1002/prot.22919 21287613

[B55] Ojo KK Larson ET Keyloun KR Castaneda LJ Derocher AE Inampudi KK Kim JE Arakaki TL Murphy RC Zhang L Napuli AJ Maly DJ Verlinde CLMJ Buckner FS Parsons M Hol WGJ Merritt EA Van Voorhis WC Toxoplasma gondii calcium-dependent protein kinase 1 is a target for selective kinase inhibitors Nature structural & molecular biology 2010 17 5 602 7 10.1038/nsmb.1818 PMC2896873 20436472

[B56] Nishida E Gotoh Y The MAP kinase cascade is essential for diverse signal transduction pathways Trends in biochemical sciences 1993 18 4 128 31 10.1016/0968-0004(93)90019-j 8388132

[B57] Srinivasan N Krupa a A genomic perspective of protein kinases in Plasmodium falciparum Proteins 2005 58 180 9 10.1002/prot.20278 15515182

[B58] Eisen JA Coyne RS Wu M Wu D Thiagarajan M Wortman JR Badger JH Ren Q Amedeo P Jones KM Tallon LJ Delcher AL Salzberg SL Silva JC Haas BJ Majoros WH Farzad M Carlton JM Smith RK Garg J Pearlman RE Karrer KM Sun L Manning G Elde NC Turkewitz AP Asai DJ Wilkes DE Wang Y Cai H Collins K Stewart BA Lee SR Wilamowska K Weinberg Z Ruzzo WL Wloga D Gaertig J Frankel J Tsao CC Gorovsky MA Keeling PJ Waller RF Patron NJ Cherry JM Stover NA Krieger CJ del Toro C Ryder HF Williamson SC Barbeau RA Hamilton EP Orias E Macronuclear genome sequence of the ciliate Tetrahymena thermophila, a model eukaryote PLoS biology 2006 4 9 e286 10.1371/journal.pbio.0040286 PMC1557398 16933976

[B59] Doerig C Billker O Pratt D Endicott J Protein kinases as targets for antimalarial intervention: Kinomics, structure-based design, transmission-blockade, and targeting host cell enzymes Biochimica et biophysica acta 2005 1754 1-2 132 50 10.1016/j.bbapap.2005.08.027 16271522

[B60] Dorin D Le Roch K Sallicandro P Alano P Parzy D Poullet P Meijer L Doerig C Pfnek-1, a NIMA-related kinase from the human malaria parasite Plasmodium falciparum European Journal of Biochemistry 2001 268 9 2600 2608 10.1046/j.1432-1327.2001.02151.x 11322879

[B61] Nunes MC Goldring JPD Doerig C Scherf A A novel protein kinase family in Plasmodium falciparum is differentially transcribed and secreted to various cellular compartments of the host cell Molecular microbiology 2007 63 2 391 403 10.1111/j.1365-2958.2006.05521.x 17181785

[B62] Nunes MC Okada M Scheidig-Benatar C Cooke BM Scherf A Plasmodium falciparum FIKK Kinase Members Target Distinct Components of the Erythrocyte Membrane PloS one 2010 5 7 e11747 10.1371/journal.pone.0011747 PMC2909202 20668526

[B63] Shaw MK Cell invasion by Theileria sporozoites Trends in parasitology 2003 19 2 6 10.1016/s1471-4922(02)00015-6 12488213

[B64] Bradley PJ Ward C Cheng SJ Alexander DL Coller S Coombs GH Dunn JD Ferguson DJ Sanderson SJ Wastling JM Boothroyd JC Proteomic analysis of rhoptry organelles reveals many novel constituents for host-parasite interactions in Toxoplasma gondii The Journal of biological chemistry 2005 280 40 34245 58 10.1074/jbc.M504158200 16002398

[B65] Boothroyd JC Dubremetz JF Kiss and spit: the dual roles of Toxoplasma rhoptries Nature reviews Microbiology 2008 6 79 88 10.1038/nrmicro1800 18059289

[B66] El Hajj H Lebrun M Arold ST Vial H Labesse G Dubremetz JF ROP18 is a rhoptry kinase controlling the intracellular proliferation of Toxoplasma gondii PLoS pathogens 2007 3 2 e14 10.1371/journal.ppat.0030014 PMC1797617 17305424

[B67] Qiu W Wernimont AK Tang K Taylor S Lunin V Schapira M Fentress S Hui R Sibley LD Novel structural and regulatory features of rhoptry secretory kinases in Toxoplasma gondii The EMBO journal 2009 28 7 969 79 10.1038/emboj.2009.24 PMC2670854 19197235

[B68] Sibley LD Ajioka JW Population structure of Toxoplasma gondii: clonal expansion driven by infrequent recombination and selective sweeps Annual review of microbiology 2008 62 329 51 10.1146/annurev.micro.62.081307.162925 18544039

[B69] Delano W The PyMOL Molecular Graphics System, Version 1.4 2011

[B70] Cock PJA Antao T Chang JT Chapman BA Cox CJ Dalke A Friedberg I Hamelryck T Kauff F Wilczynski B de Hoon MJL Biopython: freely available Python tools for computational molecular biology and bioinformatics Bioinformatics (Oxford, England) 2009 25 11 1422 3 10.1093/bioinformatics/btp163 PMC2682512 19304878

[B71] Morgan DO Cyclin-dependent kinases: engines, clocks, and microprocessors Annual review of cell and developmental biology 1997 13 261 91 10.1146/annurev.cellbio.13.1.261 9442875

[B72] Brown NR Noble ME Endicott JA Johnson LN The structural basis for specificity of substrate and recruitment peptides for cyclin-dependent kinases Nature cell biology 1999 1 7 438 43 10.1038/15674 10559988

[B73] Doerig C Endicott J Chakrabarti D Cyclin-dependent kinase homologues of Plasmodium falciparum International journal for parasitology 2002 32 13 1575 85 10.1016/s0020-7519(02)00186-8 12435442

[B74] Doerig C Sherman IW Protein kinases regulating Plasmodium proliferation and development Molecular approaches to malaria 2005 Washington, D.C.: ASM Press 290 310

[B75] Harper JF Harmon A Plants, symbiosis and parasites: a calcium signalling connection Nature reviews Molecular cell biology 2005 6 7 555 66 10.1038/nrm1679 16072038

[B76] Dvorin JD Martyn DC Patel SD Grimley JS Collins CR Hopp CS Bright aT Westenberger S Winzeler E Blackman MJ Baker Da Wandless TJ Duraisingh MT A plant-like kinase in Plasmodium falciparum regulates parasite egress from erythrocytes Science 2010 328 5980 910 2 10.1126/science.1188191 PMC3109083 20466936

[B77] Ahn YM Clare M Ensinger CL Hood MM Lord JW Lu WP Miller DF Patt WC Smith BD Vogeti L Kaufman MD Petillo Pa Wise SC Abendroth J Chun L Clark R Feese M Kim H Stewart L Flynn DL Switch control pocket inhibitors of p38-MAP kinase. Durable type II inhibitors that do not require binding into the canonical ATP hinge region Bioorganic & medicinal chemistry letters 2010 20 19 5793 8 10.1016/j.bmcl.2010.07.134 20800479

[B78] Bellon S Fitzgibbon M Fox T Hsiao H The structure of phosphorylated P38 is monomeric and reveals a conserved activation-loop conformation Structure 1999 1057 1065 10.1016/s0969-2126(99)80173-7 10508788

[B79] Li JL Targett GA Baker DA Primary structure and sexual stage-specific expression of a LAMMER protein kinase of Plasmodium falciparum International Journal for Parasitology 2001 31 4 387 392 10.1016/s0020-7519(01)00126-6 11306117

[B80] Golovkin M Reddy aS An SC35-like protein and a novel serine/arginine-rich protein interact with Arabidopsis U1-70K protein The Journal of biological chemistry 1999 274 51 36428 38 10.1074/jbc.274.51.36428 10593939

[B81] Iriko H Jin L Kaneko O Takeo S Han ET Tachibana M Otsuki H Torii M Tsuboi T A small-scale systematic analysis of alternative splicing in Plasmodium falciparum Parasitology international 2009 58 2 196 9 10.1016/j.parint.2009.02.002 19268714

[B82] Velazquez-Dones A Hagopian JC Ma CT Zhong XY Zhou H Ghosh G Fu XD Adams Ja Mass spectrometric and kinetic analysis of ASF/SF2 phosphorylation by SRPK1 and Clk/Sty The Journal of biological chemistry 2005 280 50 41761 8 10.1074/jbc.M504156200 16223727

[B83] Ngo JCK Chakrabarti S Ding JH Velazquez-Dones A Nolen B Aubol BE Adams Ja Fu XD Ghosh G Interplay between SRPK and Clk/Sty kinases in phosphorylation of the splicing factor ASF/SF2 is regulated by a docking motif in ASF/SF2 Molecular cell 2005 20 77 89 10.1016/j.molcel.2005.08.025 16209947

[B84] Emsley P Cowtan K Coot: model-building tools for molecular graphics Acta crystallographica. Section D, Biological crystallography 2004 60 Pt 12 Pt 1 2126 32 10.1107/S0907444904019158 15572765

[B85] Rodgers JT Haas W Gygi SP Puigserver P Cdc2-like kinase 2 is an insulin-regulated suppressor of hepatic gluconeogenesis Cell metabolism 2010 11 23 34 10.1016/j.cmet.2009.11.006 PMC2807620 20074525

[B86] Nikolakaki E Du C Lai J Giannakouros T Cantley L Rabinow L Phosphorylation by LAMMER protein kinases: determination of a consensus site, identification of in vitro substrates, and implications for substrate preferences Biochemistry 2002 41 6 2055 66 10.1021/bi011521h 11827553

[B87] Gileadi O Knapp S Lee WH Marsden BD Müller S Niesen FH Kavanagh KL Ball LJ von Delft F Doyle Da Oppermann UCT Sundström M The scientific impact of the Structural Genomics Consortium: a protein family and ligand-centered approach to medically-relevant human proteins Journal of structural and functional genomics 2007 8 2-3 107 19 10.1007/s10969-007-9027-2 PMC2140095 17932789

[B88] Aurrecoechea C Brestelli J Brunk BP Fischer S Gajria B Gao X Gingle A Grant G Harb OS Heiges M Innamorato F Iodice J Kissinger JC Kraemer ET Li W Miller Ja Nayak V Pennington C Pinney DF Roos DS Ross C Srinivasamoorthy G Stoeckert CJ Thibodeau R Treatman C Wang H EuPathDB: a portal to eukaryotic pathogen databases Nucleic acids research 2010 38 Database D415 9 10.1093/nar/gkp941 PMC2808945 19914931

[B89] Kannan N Taylor SS Zhai Y Venter JC Manning G Structural and functional diversity of the microbial kinome PLoS biology 2007 5 3 e17 10.1371/journal.pbio.0050017 PMC1821047 17355172

[B90] Neuwald AF Rapid detection, classification and accurate alignment of up to a million or more related protein sequences Bioinformatics 2009 25 15 1869 1875 10.1093/bioinformatics/btp342 PMC2732367 19505947

[B91] Stamatakis A RAxML-VI-HPC: maximum likelihood-based phylogenetic analyses with thousands of taxa and mixed models Bioinformatics 2006 22 21 2688 90 10.1093/bioinformatics/btl446 16928733

[B92] Kuo CH Wares JP Kissinger JC The Apicomplexan whole-genome phylogeny: an analysis of incongruence among gene trees Molecular biology and evolution 2008 25 12 2689 98 10.1093/molbev/msn213 PMC2582981 18820254

[B93] Pick C Ebersberger I Spielmann T Bruchhaus I Burmester T Phylogenomic analyses of malaria parasites and evolution of their exported proteins BMC evolutionary biology 2011 11 167 10.1186/1471-2148-11-167 PMC3146879 21676252

[B94] Xiao L Sulaiman IM Ryan UM Zhou L Atwill ER Tischler ML Zhang X Fayer R Lal Aa Host adaptation and host-parasite co-evolution in Cryptosporidium: implications for taxonomy and public health International journal for parasitology 2002 32 14 1773 85 10.1016/s0020-7519(02)00197-2 12464424

[B95] Crooks GE Hon G Chandonia Jm Brenner SE WebLogo: a sequence logo generator Genome research 2004 14 6 1188 90 10.1101/gr.849004 PMC419797 15173120

[B96] Aurrecoechea C Brestelli J Brunk BP Dommer J Fischer S Gajria B Gao X Gingle A Grant G Harb OS Heiges M Innamorato F Iodice J Kissinger JC Kraemer E Li W Miller JA Nayak V Pennington C Pinney DF Roos DS Ross C Stoeckert CJ Treatman C Wang H PlasmoDB: a functional genomic database for malaria parasites Nucleic acids research 2009 37 Database D539 43 10.1093/nar/gkn814 PMC2686598 18957442

[B97] Gajria B Bahl A Brestelli J Dommer J Fischer S Gao X Heiges M Iodice J Kissinger JC Mackey AJ Pinney DF Roos DS Stoeckert CJ Wang H Brunk BP ToxoDB: an integrated Toxoplasma gondii database resource Nucleic acids research 2008 36 Database D553 6 10.1093/nar/gkm981 PMC2238934 18003657

[B98] Armbrust EV Berges Ja Bowler C Green BR Martinez D Putnam NH Zhou S Allen AE Apt KE Bechner M Brzezinski Ma Chaal BK Chiovitti A Davis AK Demarest MS Detter JC Glavina T Goodstein D Hadi MZ Hellsten U Hildebrand M Jenkins BD Jurka J Kapitonov VV Kröger N Lau WWY Lane TW Larimer FW Lippmeier JC Lucas S Medina M Montsant A Obornik M Parker MS Palenik B Pazour GJ Richardson PM Rynearson Ta Saito Ma Schwartz DC Thamatrakoln K Valentin K Vardi A Wilkerson FP Rokhsar DS The genome of the diatom Thalassiosira pseudonana: ecology, evolution, and metabolism Science 2004 306 5693 79 86 10.1126/science.1101156 15459382

[B99] Bowler C Allen AE Badger JH Grimwood J Jabbari K Kuo A Maheswari U Martens C Maumus F Otillar RP Rayko E Salamov A Vandepoele K Beszteri B Gruber A Heijde M Katinka M Mock T Valentin K Verret F Berges Ja Brownlee C Cadoret JP Chiovitti A Choi CJ Coesel S De Martino A Detter JC Durkin C Falciatore A Fournet J Haruta M Huysman MJJ Jenkins BD Jiroutova K Jorgensen RE Joubert Y Kaplan A Kröger N Kroth PG La Roche J Lindquist E Lommer M Martin-Jézéquel V Lopez PJ Lucas S Mangogna M McGinnis K Medlin LK Montsant A Oudot-Le Secq MP Napoli C Obornik M Parker MS Petit JL Porcel BM Poulsen N Robison M Rychlewski L Rynearson Ta Schmutz J Shapiro H Siaut M Stanley M Sussman MR Taylor AR Vardi A von Dassow P Vyverman W Willis A Wyrwicz LS Rokhsar DS Weissenbach J Armbrust EV Green BR Van de Peer Y Grigoriev IV The Phaeodactylum genome reveals the evolutionary history of diatom genomes Nature 2008 456 7219 239 44 10.1038/nature07410 18923393

[B100] Penn O Privman E Ashkenazy H Landan G Graur D Pupko T GUIDANCE: a web server for assessing alignment confidence scores Nucleic acids research 2010 38 Web Server W23 8 10.1093/nar/gkq443 PMC2896199 20497997

