# Supplementary material for: Structural and evolutionary divergence of eukaryotic protein kinases in Apicomplexa
Source: BMC Evol Biol. 2011 Nov 2;11:321. doi: 10.1186/1471-2148-11-321 (PMC3239843; doi:10.1186/1471-2148-11-321)
Supplement: Additional file 4 — CDK-SCTTLRE subfamily CHAIN alignment versus the CDK family. Colorized sequence alignment and partition generated by the CHAIN program, comparing the apicomplexan-specific subfamily of CDKs to a diverse set of eukaryotic CDKs. CHAIN compares a given "query" set (here, members of the putative subfamily) to a larger "main" set (here, a diverse set of eukaryotic CDKs) and divides the main set into 3 partitions based on contrasting levels of residue conservation: a "foreground" set of sequences with residue motifs matching the query, a "background" which does not conserve the distinguishing motifs of the foreground, and an "intermediate" which contains sequences that may partially match both the foreground and background sequence motifs. The alignment summary generated by CHAIN displays only the aligned sequences in the query, but highlights the alignment columns according to the conservation patterns defining each partition. The alignment appears as three blocks, labeled "Intermediate", "Background" and "Foreground", corresponding to those partitions. Above each block is a histogram indicating residue conservation patterns unique to that sequence set; dots above each column indicate which columns form the distinguishing pattern. Thus, tall red bars above columns in the "Foreground" block indicate residues that are strikingly conserved in the foreground, but not in the background. The rows below each "Background" and "Foreground" block indicate the conserved residue types and their conservation levels within those sequence sets, in units of 10%. [file 1471-2148-11-321-S4.PDF]

Apicomplexan CDK subfamily vs. eukaryotic CDK

```
Intermediate
                            ••
unknown               1 XXXVERLGGGTYGDVYKAV......................E..........................................YYY..................................... 23*
unknown              24 ----EKLGGGTYGDVYKGKvikynnnnnnndlyqntnylpyD..........................................YYT..................................... 64
unknown              24 ----EKLGGGTYGDVYKGKvikynnnnnndlyqntnylpy.D..........................................YYT..................................... 63
unknown              24 ----EKLGGGTYGDVYKGKvikysnsssndlyqntnylpy.D..........................................YYT..................................... 63
unknown              24 ----EKLGGGTYGDVYKGK......................V..........................................VKY..................................... 42
unknown              24 ----EKLGGGTYGDVYKGK......................V..........................................VKY..................................... 42
unknown              24 ----EKLGGGTYGDVYKGK......................V..........................................VKY..................................... 42
unknown             131 -------------------......................-..........................................---.....................................
unknown             131 -------------------......................-..........................................---.....................................
unknown             127 -------------------......................-..........................................---.....................................
unknown              34 ---VERLGGGTYGDVYKAV......................EeeeeeendrgsarardrpgistaeataeatngedasakegsekKTK..................................... 95
unknown              34 ---VERLGGGTYGDVYKAV......................EeeeeeendrgsarardrpgistaeataeatngedasakegsekKTK..................................... 95
unknown              34 ---VERLGGGTYGDVYKAV......................EeeeeeendrgsarardrpgistaeataeatngedasakegsekKTK..................................... 95
unknown              34 ---VERLGGGTYGDVYKAV......................E..........................................EEDeenqrgassslssasrdgasdggrgnveetpekegea 90
unknown              34 ---VERLGGGTYGDVYRAV......................E..........................................EPEdgvgg................................ 58
position                    .   10    .                         20
```

```
Background
                        •     •• •••• ••••                                                                     •
consensus             1 XXXVERLGGGTYGDVYKAV......................E..........................................YYY..................................... 23*
PFF0750w             24 ----EKLGGGTYGDVYKGKvikynnnnnnndlyqntnylpyD..........................................YYT..................................... 64
PKH_113350           24 ----EKLGGGTYGDVYKGKvikynnnnnndlyqntnylpy.D..........................................YYT..................................... 63
PVX_113910           24 ----EKLGGGTYGDVYKGKvikysnsssndlyqntnylpy.D..........................................YYT..................................... 63
PBANKA_123020        24 ----EKLGGGTYGDVYKGK......................V..........................................VKY..................................... 42
PY06538              24 ----EKLGGGTYGDVYKGK......................V..........................................VKY..................................... 42
PCHAS_123090         24 ----EKLGGGTYGDVYKGK......................V..........................................VKY..................................... 42
TA09960             131 -------------------......................-..........................................---.....................................
TP04_0791           131 -------------------......................-..........................................---.....................................
BBOV_III008880      127 -------------------......................-..........................................---.....................................
TGGT1_118720         34 ---VERLGGGTYGDVYKAV......................EeeeeeendrgsarardrpgistaeataeatngedasakegsekKTK..................................... 95
TGME49_029020        34 ---VERLGGGTYGDVYKAV......................EeeeeeendrgsarardrpgistaeataeatngedasakegsekKTK..................................... 95
TGVEG_026470         34 ---VERLGGGTYGDVYKAV......................EeeeeeendrgsarardrpgistaeataeatngedasakegsekKTK..................................... 95
NCLIV_030060         34 ---VERLGGGTYGDVYKAV......................E..........................................EEDeenqrgassslssasrdgasdggrgnveetpekegea 90
ETH_00023650         34 ---VERLGGGTYGDVYRAV......................E..........................................EPEdgvgg................................ 58
foreground (196):       YEKIEKVGEGTYGVVYKGK                      N                                          KET                                      15.8
                        FQILN L S SFAT FRAR                      D                                          RQN
                            G I   A       I                      H                                          TK
wt_res_freqs (61):      6221241769578296611                      1                                          315
                        21131 3 1 1111 1162                      4                                          111
                            1 4   1       1                      1                                          11
insertions                  1                                                                               127
deletions               7776655554444444444                      5                                          433                                      11.5
```

```
Foreground
                                •    •
consensus             1 XXXVERLGGGTYGDVYKAV......................E..........................................YYY..................................... 23*
PFF0750w             24 ----EKLGGGTYGDVYKGKvikynnnnnnndlyqntnylpyD..........................................YYT..................................... 64
PKH_113350           24 ----EKLGGGTYGDVYKGKvikynnnnnndlyqntnylpy.D..........................................YYT..................................... 63
PVX_113910           24 ----EKLGGGTYGDVYKGKvikysnsssndlyqntnylpy.D..........................................YYT..................................... 63
PBANKA_123020        24 ----EKLGGGTYGDVYKGK......................V..........................................VKY..................................... 42
PY06538              24 ----EKLGGGTYGDVYKGK......................V..........................................VKY..................................... 42
PCHAS_123090         24 ----EKLGGGTYGDVYKGK......................V..........................................VKY..................................... 42
TA09960             131 -------------------......................-..........................................---.....................................
TP04_0791           131 -------------------......................-..........................................---.....................................
BBOV_III008880      127 -------------------......................-..........................................---.....................................
TGGT1_118720         34 ---VERLGGGTYGDVYKAV......................EeeeeeendrgsarardrpgistaeataeatngedasakegsekKTK..................................... 95
TGME49_029020        34 ---VERLGGGTYGDVYKAV......................EeeeeeendrgsarardrpgistaeataeatngedasakegsekKTK..................................... 95
TGVEG_026470         34 ---VERLGGGTYGDVYKAV......................EeeeeeendrgsarardrpgistaeataeatngedasakegsekKTK..................................... 95
NCLIV_030060         34 ---VERLGGGTYGDVYKAV......................E..........................................EEDeenqrgassslssasrdgasdggrgnveetpekegea 90
ETH_00023650         34 ---VERLGGGTYGDVYRAV......................E..........................................EPEdgvgg................................ 58
foreground (15):           VEKLGGGTYGDVYRGK                      D                                          ETT                                      1.3
                             R           AV                      V                                          VPY
                                                                 E                                          YYK
wt_res_freqs (4):          3737777777777133                      1                                          111
                             3           33                      1                                          111
                                                                 3                                          111
insertions                                 2                     2                                            1
deletions               9996222222222222222                      2                                          222                                      2.3
position                    .   10    .                         20
```

Intermediate

```
unknown              24 .......GN...........................FY.......AIKYFKDDT...N...EEGISCTTVRELSCMKNC............H.HPNIVRMIDV..................... 66*
unknown              65 .......DE...........................FVffrknvyAIKFFRDDLrtiN...EEGISCTTLRELSCLKNIg...........R.HPNILRLIDV..................... 118
unknown              64 .......DE...........................FVffrkniyAIKFFRDDL...KtinEEGISCTTLRELSCLKNIg...........R.HPNILRLIDV..................... 117
unknown              64 .......DE...........................FVffrkniyAIKFFRDDL...KtinDEGISCTTLRELSCLKNIg...........R.HPNILRLIDV..................... 117
unknown              43 .......NNnnsdlyqntnylpydyytdefiffrknIY.......AIKFFKDDLrtiN...EEGISCTTLRELSCLKNIg...........R.HPNILRLIDVtidrqkgiseyinrqilqhyt 137
unknown              43 .......NNnnndlyqntnylpydyytddfiffrknIY.......AIKFFKDDLrtiN...EEGISCTTLRELSCLKNIg...........R.HPNILRLIDVtidrqkgiseyinrqilqhyt 137
unknown              43 .......NNnsdlyqntnylpydyytdefiffrkn.IY.......AIKFFKDDLrtiN...EEGISCTTLRELSCLKNIg...........R.HPNILRLIDV..................... 115
unknown             131 .......--...........................-Y.......AIKYFKDDSiqlL...DEGFTSGTIRELSIMKSV............SgHPNIVKLVDI..................... 174
unknown             131 .......--...........................-Y.......AIKYFKDDSiqlL...DEGFTSGTIRELSIMKSV............SgHPNIVKLVDI..................... 174
unknown             127 .......--...........................VY.......AVKYFKDDI...VhimEEGISPGTLRELSIMKVCrssgkynvqscnD.HPNIVKLVDV..................... 182
unknown              96 .......GN...........................FY.......AVKYYKDET...RtimEEGISCTTVRELSAVAGCg...........H.HPNVVRMESL..................... 142
unknown              96 .......GN...........................FY.......AVKYYKDET...RtimEEGISCTTVRELSAVAGCg...........H.HPNVVRMESL..................... 142
unknown              96 .......GN...........................FY.......AVKYYKDET...RtimEEGISCTTVRELSAVAGCg...........H.HPNVVRMESL..................... 142
unknown              91 kekqegkGN...........................LY.......AVKYYKDET...RtimEEGISCTTVRELSAVAGCg...........H.HPNVVRMESL..................... 144
unknown              59 .......GE...........................TF.......AVKYYVDETrtaT...EDGLGCTTIRELSTISSCg...........T.HPNLVRMRYL..................... 105
position                        .                                    30      .        40   . 50   .                    60   .
```

Background

```
consensus            24 .......GN...........................FY.......AIKYFKDDT...N...EEGISCTTVRELSCMKNC............H.HPNIVRMIDV..................... 66*
PFF0750w             65 .......DE...........................FVffrknvyAIKFFRDDLrtiN...EEGISCTTLRELSCLKNIg...........R.HPNILRLIDV..................... 118
PKH_113350           64 .......DE...........................FVffrkniyAIKFFRDDL...KtinEEGISCTTLRELSCLKNIg...........R.HPNILRLIDV..................... 117
PVX_113910           64 .......DE...........................FVffrkniyAIKFFRDDL...KtinDEGISCTTLRELSCLKNIg...........R.HPNILRLIDV..................... 117
PBANKA_123020        43 .......NNnnsdlyqntnylpydyytdefiffrknIY.......AIKFFKDDLrtiN...EEGISCTTLRELSCLKNIg...........R.HPNILRLIDVtidrqkgiseyinrqilqhyt 137
PY06538              43 .......NNnnndlyqntnylpydyytddfiffrknIY.......AIKFFKDDLrtiN...EEGISCTTLRELSCLKNIg...........R.HPNILRLIDVtidrqkgiseyinrqilqhyt 137
PCHAS_123090         43 .......NNnsdlyqntnylpydyytdefiffrkn.IY.......AIKFFKDDLrtiN...EEGISCTTLRELSCLKNIg...........R.HPNILRLIDV..................... 115
TA09960             131 .......--...........................-Y.......AIKYFKDDSiqlL...DEGFTSGTIRELSIMKSV............SgHPNIVKLVDI..................... 174
TP04_0791           131 .......--...........................-Y.......AIKYFKDDSiqlL...DEGFTSGTIRELSIMKSV............SgHPNIVKLVDI..................... 174
BBOV_III008880      127 .......--...........................VY.......AVKYFKDDI...VhimEEGISPGTLRELSIMKVCrssgkynvqscnD.HPNIVKLVDV..................... 182
TGGT1_118720         96 .......GN...........................FY.......AVKYYKDET...RtimEEGISCTTVRELSAVAGCg...........H.HPNVVRMESL..................... 142
TGME49_029020        96 .......GN...........................FY.......AVKYYKDET...RtimEEGISCTTVRELSAVAGCg...........H.HPNVVRMESL..................... 142
TGVEG_026470         96 .......GN...........................FY.......AVKYYKDET...RtimEEGISCTTVRELSAVAGCg...........H.HPNVVRMESL..................... 142
NCLIV_030060         91 kekqegkGN...........................LY.......AVKYYKDET...RtimEEGISCTTVRELSAVAGCg...........H.HPNVVRMESL..................... 144
ETH_00023650         59 .......GE...........................TF.......AVKYYVDETrtaT...EDGLGCTTIRELSTISSCg...........T.HPNLVRMRYL..................... 105
foreground (196):              GE                           VV       AIKKVKLDN   E   DDGVPSSAIREIAILKEL            K HPNVVKLKDV                      15.8
                               NQ                           L         L RIRMES       EE ISITSL  VSL RQ             R  E IIR REI
                               KK                           I            F   K       K  F   T    K  QK             N  A   S H
wt_res_freqs (61):             43                           17       829413321   4   129262253896138437            2 8482627144
                               11                           1         5 143121       35 211514  133 21             1  1 621 123
                               11                           2            1   1       1  2   1    2  11              2  1   1 1
insertions                     1                                         1 114   2   51               3            8 21   1   1
deletions                      22                           33       333213234   1   333224444222222222            2 2222222211                      11.5
```

Foreground

```
consensus            24 .......GN...........................FY.......AIKYFKDDT...N...EEGISCTTVRELSCMKNC............H.HPNIVRMIDV..................... 66*
PFF0750w             65 .......DE...........................FVffrknvyAIKFFRDDLrtiN...EEGISCTTLRELSCLKNIg...........R.HPNILRLIDV..................... 118
PKH_113350           64 .......DE...........................FVffrkniyAIKFFRDDL...KtinEEGISCTTLRELSCLKNIg...........R.HPNILRLIDV..................... 117
PVX_113910           64 .......DE...........................FVffrkniyAIKFFRDDL...KtinDEGISCTTLRELSCLKNIg...........R.HPNILRLIDV..................... 117
PBANKA_123020        43 .......NNnnsdlyqntnylpydyytdefiffrknIY.......AIKFFKDDLrtiN...EEGISCTTLRELSCLKNIg...........R.HPNILRLIDVtidrqkgiseyinrqilqhyt 137
PY06538              43 .......NNnnndlyqntnylpydyytddfiffrknIY.......AIKFFKDDLrtiN...EEGISCTTLRELSCLKNIg...........R.HPNILRLIDVtidrqkgiseyinrqilqhyt 137
PCHAS_123090         43 .......NNnsdlyqntnylpydyytdefiffrkn.IY.......AIKFFKDDLrtiN...EEGISCTTLRELSCLKNIg...........R.HPNILRLIDV..................... 115
TA09960             131 .......--...........................-Y.......AIKYFKDDSiqlL...DEGFTSGTIRELSIMKSV............SgHPNIVKLVDI..................... 174
TP04_0791           131 .......--...........................-Y.......AIKYFKDDSiqlL...DEGFTSGTIRELSIMKSV............SgHPNIVKLVDI..................... 174
BBOV_III008880      127 .......--...........................VY.......AVKYFKDDI...VhimEEGISPGTLRELSIMKVCrssgkynvqscnD.HPNIVKLVDV..................... 182
TGGT1_118720         96 .......GN...........................FY.......AVKYYKDET...RtimEEGISCTTVRELSAVAGCg...........H.HPNVVRMESL..................... 142
TGME49_029020        96 .......GN...........................FY.......AVKYYKDET...RtimEEGISCTTVRELSAVAGCg...........H.HPNVVRMESL..................... 142
TGVEG_026470         96 .......GN...........................FY.......AVKYYKDET...RtimEEGISCTTVRELSAVAGCg...........H.HPNVVRMESL..................... 142
NCLIV_030060         91 kekqegkGN...........................LY.......AVKYYKDET...RtimEEGISCTTVRELSAVAGCg...........H.HPNVVRMESL..................... 144
ETH_00023650         59 .......GE...........................TF.......AVKYYVDETrtaT...EDGLGCTTIRELSTISSCg...........T.HPNLVRMRYL..................... 105
foreground (15):               GN                           TY       AVKYFRDES   T   EEGFSCGTVRELSCVAGC            S HPNVVRLESV                      1.3
                               DE                           IV          FYV  L   L   DD LTS  L    IMSNI            H    LLKMIYL
                               N                            FF               T   R       G   I    TL SV            R    I   V I
wt_res_freqs (4):              34                           17       949661931   1   789177292999932224            1 9992676224
                               12                           11          331  3   1   21 111  4    22133            2    1323313
                               1                            31               3   2       1   2    13 21            3    6   2 1
insertions                      2                            2               4   4                    8            1          1
deletions                      22                           1                                                                                    2.3
position                        .                                    30      .        40   . 50   .                    60   .
```

```
Intermediate
unknown          67  ..F....................................V............................................QQL.....................................  71*
unknown         119  ..T....................................I............................................D--.....................................  121
unknown         118  ..T....................................I............................................D--.....................................  120
unknown         118  ..T....................................I............................................D--.....................................  120
unknown         138  snY....................................H............................................H--.....................................  142
unknown         138  snY....................................H............................................H--.....................................  142
unknown         116  ..T....................................I............................................D--.....................................  118
unknown         175  ..F....................................V............................................G--.....................................  177
unknown         175  ..F....................................V............................................G--.....................................  177
unknown         183  ..YvgphpgivdmlnqqlgdalkssgtevagrkdlqffpF............................................K--.....................................  221
unknown         143  ..F....................................VdplprlarainqqrltwarnqqsalssqqfshlqeqlqkeelkpQ--.....................................  189
unknown         143  ..F....................................VdplprlarainqqrltwarnqqsalssqqfshlqeqlqkeelkpQ--.....................................  189
unknown         143  ..F....................................VdplprlarainqqrltwarnqqsalssqqfshlqeqlqkeelkpQ--.....................................  189
unknown         145  ..F....................................V............................................DPLprlaravnqqrvawarsqqsslssqqyshlqnqiqke  186
unknown         106  ..F....................................V............................................DQLprlveninqqriqwarqqhaaadresqeqlrrdlqtq  147
position                                                                                                     70
```

```
Background
consensus        67  ..F....................................V............................................QQL.....................................  71*
PFF0750w        119  ..T....................................I............................................D--.....................................  121
PKH_113350      118  ..T....................................I............................................D--.....................................  120
PVX_113910      118  ..T....................................I............................................D--.....................................  120
PBANKA_123020   138  snY....................................H............................................H--.....................................  142
PY06538         138  snY....................................H............................................H--.....................................  142
PCHAS_123090    116  ..T....................................I............................................D--.....................................  118
TA09960         175  ..F....................................V............................................G--.....................................  177
TP04_0791       175  ..F....................................V............................................G--.....................................  177
BBOV_III008880  183  ..YvgphpgivdmlnqqlgdalkssgtevagrkdlqffpF............................................K--.....................................  221
TGGT1_118720    143  ..F....................................VdplprlarainqqrltwarnqqsalssqqfshlqeqlqkeelkpQ--.....................................  189
TGME49_029020   143  ..F....................................VdplprlarainqqrltwarnqqsalssqqfshlqeqlqkeelkpQ--.....................................  189
TGVEG_026470    143  ..F....................................VdplprlarainqqrltwarnqqsalssqqfshlqeqlqkeelkpQ--.....................................  189
NCLIV_030060    145  ..F....................................V............................................DPLprlaravnqqrvawarsqqsslssqqyshlqnqiqke  186
ETH_00023650    106  ..F....................................V............................................DQLprlveninqqriqwarqqhaaadresqeqlrrdlqtq  147
foreground (196):      V                                    H                                            S                                         15.8
                       I                                    L                                            T
                       F                                                                                 G
wt_res_freqs (61):     2                                    1                                            1
                       2                                    1                                            1
                       1                                                                                 1
insertions             5                                    4                                            11
deletions              1                                    4                                            955                                       11.5
```

```
Foreground
consensus        67  ..F....................................V............................................QQL.....................................  71*
PFF0750w        119  ..T....................................I............................................D--.....................................  121
PKH_113350      118  ..T....................................I............................................D--.....................................  120
PVX_113910      118  ..T....................................I............................................D--.....................................  120
PBANKA_123020   138  snY....................................H............................................H--.....................................  142
PY06538         138  snY....................................H............................................H--.....................................  142
PCHAS_123090    116  ..T....................................I............................................D--.....................................  118
TA09960         175  ..F....................................V............................................G--.....................................  177
TP04_0791       175  ..F....................................V............................................G--.....................................  177
BBOV_III008880  183  ..YvgphpgivdmlnqqlgdalkssgtevagrkdlqffpF............................................K--.....................................  221
TGGT1_118720    143  ..F....................................VdplprlarainqqrltwarnqqsalssqqfshlqeqlqkeelkpQ--.....................................  189
TGME49_029020   143  ..F....................................VdplprlarainqqrltwarnqqsalssqqfshlqeqlqkeelkpQ--.....................................  189
TGVEG_026470    143  ..F....................................VdplprlarainqqrltwarnqqsalssqqfshlqeqlqkeelkpQ--.....................................  189
NCLIV_030060    145  ..F....................................V............................................DPLprlaravnqqrvawarsqqsslssqqyshlqnqiqke  186
ETH_00023650    106  ..F....................................V............................................DQLprlveninqqriqwarqqhaaadresqeqlrrdlqtq  147
foreground (15):       T                                    H                                            GQL                                       1.3
                       Y                                    I                                            H
                                                                                                         Q
wt_res_freqs (4):      2                                    1                                            111
                       2                                    2                                            1
                                                                                                         1
insertions             6                                    2                                              1
deletions                                                                                                 88                                       2.3
position                                                                                                 70
```

```
Intermediate
unknown          72  .....QQK....................................IF..................................MA............Y...EYCPG......DL.....KKLIQ...  91*
unknown         122  .....RQK....................................LIseyinrqilqhytsnyhhhhhsklditplaadqkFI............FaayEYCDGg.....DL.....KKLIQ...  179
unknown         121  .....RQKciseyinrqilqhhasnfqhskkldmtplsadqkf.IF..................................AA............Y...EYCDAg.....DL.....KRLIQ...  176
unknown         121  .....RQKciseyinrqilqhhasnfqhsakldmtplaadqkf.IF..................................AA............Y...EYCDAg.....DL.....KRLIQ...  176
unknown         143  .....QVK....................................ID..................................MVplsldqkfifaaY...EYCDGg.....DL.....KKLIQktk  178
unknown         143  .....QVK....................................ID..................................MVplsldqkfifaaY...EYCDGg.....DL.....KKLIQ...  175
unknown         119  .....RQKgiseyinrqilqhytsnyhhqvkidmaplsldqkf.IF..................................AA............Y...EYCDGg.....DL.....KKLIQktk  177
unknown         178  .....EHEgitnklnnqiseliksdsrlspkefgfypfkntqifVL..................................GL............Y...EFCRGg.....DLgrgifRKFSK...  239
unknown         178  .....GDL....................................GR..................................GI............F...-----......--.....RKFTK...  190
unknown         222  .....KSQ....................................IF..................................MFal..........Y...EYCEGg.....DLgraiwRAYSK...  249
unknown         190  .....QNF....................................VF..................................AA............Y...EFCPGg.....DL.....KKLLA...  210
unknown         190  .....QNF....................................VF..................................AA............Y...EFCPGg.....DL.....KKLLA...  210
unknown         190  .....QNF....................................VF..................................AA............Y...EFCPGg.....DL.....KKLLA...  210
unknown         187  elkpnQNF....................................VF..................................AA............Y...EFCPGg.....DL.....KKLLA...  212
unknown         148  laqe.ELK....................................PT..................................QV............F...VFAAYelceggDL.....KKLLA...  177
position                                                     .                                                          80          .        90

Background
consensus        72  .....QQK....................................IF..................................MA............Y...EYCPG......DL.....KKLIQ...  91*
PFF0750w        122  .....RQK....................................LIseyinrqilqhytsnyhhhhhsklditplaadqkFI............FaayEYCDGg.....DL.....KKLIQ...  179
PKH_113350      121  .....RQKciseyinrqilqhhasnfqhskkldmtplsadqkf.IF..................................AA............Y...EYCDAg.....DL.....KRLIQ...  176
PVX_113910      121  .....RQKciseyinrqilqhhasnfqhsakldmtplaadqkf.IF..................................AA............Y...EYCDAg.....DL.....KRLIQ...  176
PBANKA_123020   143  .....QVK....................................ID..................................MVplsldqkfifaaY...EYCDGg.....DL.....KKLIQktk  178
PY06538         143  .....QVK....................................ID..................................MVplsldqkfifaaY...EYCDGg.....DL.....KKLIQ...  175
PCHAS_123090    119  .....RQKgiseyinrqilqhytsnyhhqvkidmaplsldqkf.IF..................................AA............Y...EYCDGg.....DL.....KKLIQktk  177
TA09960         178  .....EHEgitnklnnqiseliksdsrlspkefgfypfkntqifVL..................................GL............Y...EFCRGg.....DLgrgifRKFSK...  239
TP04_0791       178  .....GDL....................................GR..................................GI............F...-----......--.....RKFTK...  190
BBOV_III008880  222  .....KSQ....................................IF..................................MFal..........Y...EYCEGg.....DLgraiwRAYSK...  249
TGGT1_118720    190  .....QNF....................................VF..................................AA............Y...EFCPGg.....DL.....KKLLA...  210
TGME49_029020   190  .....QNF....................................VF..................................AA............Y...EFCPGg.....DL.....KKLLA...  210
TGVEG_026470    190  .....QNF....................................VF..................................AA............Y...EFCPGg.....DL.....KKLLA...  210
NCLIV_030060    187  elkpnQNF....................................VF..................................AA............Y...EFCPGg.....DL.....KKLLA...  212
ETH_00023650    148  laqe.ELK....................................PT..................................QV............F...VFAAYelceggDL.....KKLLA...  177
foreground (196):         DDS                                         VY                                  IV            F   EYMDH       DL       KKYID      15.8
                          EGK                                         LF                                  LI            M   DFVEY                REFLE
                          KS                                          IT                                  M                   L M                   QIMK
wt_res_freqs (61):        111                                         13                                  17            6   75232       89       21331
                          111                                         41                                  51            1   12131                11142
                          11                                          21                                  1                     1 1                 1111
insertions                222                                                                                                                         11
deletions                 211                                         11                                  12            1   11122       22       22222      11.5

Foreground
consensus        72  .....QQK....................................IF..................................MA............Y...EYCPG......DL.....KKLIQ...  91*
PFF0750w        122  .....RQK....................................LIseyinrqilqhytsnyhhhhhsklditplaadqkFI............FaayEYCDGg.....DL.....KKLIQ...  179
PKH_113350      121  .....RQKciseyinrqilqhhasnfqhskkldmtplsadqkf.IF..................................AA............Y...EYCDAg.....DL.....KRLIQ...  176
PVX_113910      121  .....RQKciseyinrqilqhhasnfqhsakldmtplaadqkf.IF..................................AA............Y...EYCDAg.....DL.....KRLIQ...  176
PBANKA_123020   143  .....QVK....................................ID..................................MVplsldqkfifaaY...EYCDGg.....DL.....KKLIQktk  178
PY06538         143  .....QVK....................................ID..................................MVplsldqkfifaaY...EYCDGg.....DL.....KKLIQ...  175
PCHAS_123090    119  .....RQKgiseyinrqilqhytsnyhhqvkidmaplsldqkf.IF..................................AA............Y...EYCDGg.....DL.....KKLIQktk  177
TA09960         178  .....EHEgitnklnnqiseliksdsrlspkefgfypfkntqifVL..................................GL............Y...EFCRGg.....DLgrgifRKFSK...  239
TP04_0791       178  .....GDL....................................GR..................................GI............F...-----......--.....RKFTK...  190
BBOV_III008880  222  .....KSQ....................................IF..................................MFal..........Y...EYCEGg.....DLgraiwRAYSK...  249
TGGT1_118720    190  .....QNF....................................VF..................................AA............Y...EFCPGg.....DL.....KKLLA...  210
TGME49_029020   190  .....QNF....................................VF..................................AA............Y...EFCPGg.....DL.....KKLLA...  210
TGVEG_026470    190  .....QNF....................................VF..................................AA............Y...EFCPGg.....DL.....KKLLA...  210
NCLIV_030060    187  elkpnQNF....................................VF..................................AA............Y...EFCPGg.....DL.....KKLLA...  212
ETH_00023650    148  laqe.ELK....................................PT..................................QV............F...VFAAYelceggDL.....KKLLA...  177
foreground (15):          ENK                                         VT                                  GA            Y    VYCAG      DL       KKLSA      1.3
                          RLF                                         PD                                  MI            F     FAPY               RRFLK
                          QV                                          I                                   QV                   DA                   IQ
wt_res_freqs (4):         224                                         31                                  14            7   14716       99       77713
                          212                                         11                                  21            2    4121                21132
                          31                                          4                                   12                    31                   33
insertions                  2                                          6                                   2            6      8          1            1
deletions                                                                                                                   66666       66                  2.3
position                                                     .                                                          80          .        90
```

```
Intermediate
unknown               92 .MHR.....A...................G..........LPLRHAKWLSFQLLNGLAYLHNNKMCHRDLKPENVML....NRTY....VLKIGDWGLCREFRYNDGDMTPTVCTIYYRPLEVL 172*
unknown              180 .KTKisddqA...................G..........LNLKEAKWLSFQLLNGLAYLHNNKMCHRDLKPENVMLqetsNHKY....LLKIGDLGLCREL-KNDGDMTPTVCTIYYRPLEVL 268
unknown              177 .KTKitddqA...................G..........LSLKEAKWLSFQLLNGLAYLHNNKMCHRDLKPENVMLqhtpNRKY....LLKIGDLGLCREL-KNDGDMTPTVCTIYYRPLEVL 265
unknown              177 .KTKitddqA...................G..........LSLKEAKWLSFQLLNGLAYLHNNKMCHRDLKPENVMLqqtaNRKF....LLKIGDLGLCREL-KNDGDMTPTVCTIYYRPLEVL 265
unknown              179 iSENq....A...................G..........LCLKEAKWLSFQLLNGLAYLHNNKMCHRDLKPENVMLqythNNKY....LLKIGDLGLCREL-KNDGDMTPTVCTIYYRPLEVL 264
unknown              176 .KTKinenqA...................G..........LCLKEAKWLSFQLLNGLAYLHNNKMCHRDLKPENVMLqythNNKY....LLKIGDLGLCREL-KNDGDMTPTVCTIYYRPLEVL 264
unknown              178 iSENq....A...................G..........LCLKEAKWLSFQLLNGLAYLHNNKMCHRDLKPENVMLqythNNKY....LLKIGDLGLCREL-KNDGDMTPTVCTIYYRPLEVL 263
unknown              240 .SSEg....-...................-..........FSLQEVKWLSFQLLNGLAYLHNLKIEHRDLKPNNIML....DREGpfp.VLKIGDWGLGREFRSLHGTITPTACTMFYRPIEVI 322
unknown              191 .SSEg....F...................S..........IPE--VKWLSFQLLNGLAYLHNLKIEHRDLKPNNIML....DRDGpfp.VLKIGDWGLGREFRSLHGTITPTACTMFYRPIEVI 273
unknown              250 .NQPg....Y...................S..........LSE--VKWYAFQLLNGLAYLHMSKIEHRDLKPNNIML....SDSErlt.VLKIGDWGMGREFRNLDGTVTPTACTLFYRPIEVI 332
unknown              211 .MHRq....Sagnqesgtgsppplsw...G..........LPLRHVKRLAFQLLNGLAFLHSENLCHRDLKPDNLML....TGTDpetaVLKIGDLGLCRELRYNVGDITPTVCTIYYRSLEVL 312
unknown              211 .MHRq....Sagnqesgtgsppplsw...G..........LPLRHVKRLAFQLLNGLAFLHSENLCHRDLKPDNLML....TGTDpetaVLKIGDLGLCRELRYNVGDITPTVCTIYYRSLEVL 312
unknown              211 .MHRq....Sagnqesgtgsppplsw...G..........LPLRHVKRLAFQLLNGLAFLHSENLCHRDLKPDNLML....TGTDpetaVLKIGDLGLCRELRYNVGDITPTVCTIYYRSLEVL 312
unknown              213 .LHRq....GaggnanqesgtgtppplswG..........LPLRHAKRLAFQLLNGLAFLHSENLCHRDLKPDNLML....TGTDpetaVLKIGDLGLCRELRYNVGDITPTVCTIYYRSLEVL 317
unknown              178 .KQRe....N...................KpsssvspewgLPLRHAKLLAFQLLNGLAFLHNEKLCHRDLKPDNLMLtse.NENC....VLKIGDLGLCRSFRANAGEITPTVCTIYYRPLEVL 272
position                          .                                100    .  110    .  120    .  130        .      140    .  150    .  160    .  170

Eukaryota
ETH_00023650         178 .KQRe....N...................KpsssvspewgLPLRHAKLLAFQLLNGLAFLHNEKLCHRDLKPDNLMLtse.NENC....VLKIGDLGLCRSFRANAGEITPTVCTIYYRPLEVL 272
background (196):         NHP     V                   P          ISPAQVKSYMYQILRGVAYCHSNWVIHRDIKPQNILV    NNNG    EVKIGDFGLARAYGVPVKPYSHEVVTLWYRAPEVL 15.8
                          SNG     K                   G          MPESEIRLFLF L EALNFL RHRIM   L  A L L    DKD     QL LA    S SFSI LRTLTNV I R   P  L
                          K                                      LDLNL   I K     IEHI RK  L      S   I    S K     VI         L  S I K  S           I
conservation:             112     1                   1          1221136313282828215392312199929739492    2117    12841999878222161115123758689949818
                          111     1                   1          11111311221 6 114123 11261   6  1 4 2    111     15 45    1 1422 1311611 1 1   4  5
                          1                                      31111   1 1     1111  11 5      2   4    2 1     11         1  1 1 1  1           2
insertions                114     2                   3               1                                   9 21    4     1    1 3 1511
deletions                 418     1                   8          4444544221112122222211111111111          1522    11  1  1111111445211211111111111111 11.5

Apicomplexa
consensus             92 .MHR.....A...................G..........LPLRHAKWLSFQLLNGLAYLHNNKMCHRDLKPENVML....NRTY....VLKIGDWGLCREFRYNDGDMTPTVCTIYYRPLEVL 172*
P. falciparum        180 .KTKisddqA...................G..........LNLKEAKWLSFQLLNGLAYLHNNKMCHRDLKPENVMLqetsNHKY....LLKIGDLGLCREL-KNDGDMTPTVCTIYYRPLEVL 268
P. knowlesi          177 .KTKitddqA...................G..........LSLKEAKWLSFQLLNGLAYLHNNKMCHRDLKPENVMLqhtpNRKY....LLKIGDLGLCREL-KNDGDMTPTVCTIYYRPLEVL 265
P. vivax             177 .KTKitddqA...................G..........LSLKEAKWLSFQLLNGLAYLHNNKMCHRDLKPENVMLqqtaNRKF....LLKIGDLGLCREL-KNDGDMTPTVCTIYYRPLEVL 265
P. berghei           179 iSENq....A...................G..........LCLKEAKWLSFQLLNGLAYLHNNKMCHRDLKPENVMLqythNNKY....LLKIGDLGLCREL-KNDGDMTPTVCTIYYRPLEVL 264
P. yoelii            176 .KTKinenqA...................G..........LCLKEAKWLSFQLLNGLAYLHNNKMCHRDLKPENVMLqythNNKY....LLKIGDLGLCREL-KNDGDMTPTVCTIYYRPLEVL 264
P. chabaudi          178 iSENq....A...................G..........LCLKEAKWLSFQLLNGLAYLHNNKMCHRDLKPENVMLqythNNKY....LLKIGDLGLCREL-KNDGDMTPTVCTIYYRPLEVL 263
T. annulata          240 .SSEg....-...................-..........FSLQEVKWLSFQLLNGLAYLHNLKIEHRDLKPNNIML....DREGpfp.VLKIGDWGLGREFRSLHGTITPTACTMFYRPIEVI 322
T. parva             191 .SSEg....F...................S..........IPE--VKWLSFQLLNGLAYLHNLKIEHRDLKPNNIML....DRDGpfp.VLKIGDWGLGREFRSLHGTITPTACTMFYRPIEVI 273
B. bovis             250 .NQPg....Y...................S..........LSE--VKWYAFQLLNGLAYLHMSKIEHRDLKPNNIML....SDSErlt.VLKIGDWGMGREFRNLDGTVTPTACTLFYRPIEVI 332
T. gondii GT1        211 .MHRq....Sagnqesgtgsppplsw...G..........LPLRHVKRLAFQLLNGLAFLHSENLCHRDLKPDNLML....TGTDpetaVLKIGDLGLCRELRYNVGDITPTVCTIYYRSLEVL 312
T. gondii ME49       211 .MHRq....Sagnqesgtgsppplsw...G..........LPLRHVKRLAFQLLNGLAFLHSENLCHRDLKPDNLML....TGTDpetaVLKIGDLGLCRELRYNVGDITPTVCTIYYRSLEVL 312
T. gondii VEG        211 .MHRq....Sagnqesgtgsppplsw...G..........LPLRHVKRLAFQLLNGLAFLHSENLCHRDLKPDNLML....TGTDpetaVLKIGDLGLCRELRYNVGDITPTVCTIYYRSLEVL 312
N. caninum           213 .LHRq....GaggnanqesgtgtppplswG..........LPLRHAKRLAFQLLNGLAFLHSENLCHRDLKPDNLML....TGTDpetaVLKIGDLGLCRELRYNVGDITPTVCTIYYRSLEVL 317
E. tenella           178 .KQRe....N...................KpsssvspewgLPLRHAKLLAFQLLNGLAFLHNEKLCHRDLKPDNLMLtse.NENC....VLKIGDLGLCRSFRANAGEITPTVCTIYYRPLEVL 272
foreground (15):          SSN     A                   G          LCLKEAKWLAFQLLNGLAFLHNNKICHRDLKPNNVML    TGTC    LLKIGDLGLCRELRANAGDITPTACTIYYRSLEVI 1.3
                          MHR     N                   K           PERHV L             SLNME      E L      DRKY          W  G SF YLV EM   V  MF   I  L
                          KQK     S                   S           S     R              E L       D I      NEND                        K H T
conservation:             211     3                   6          8183459684999999993996372799999929399    2221    39999979879866171965999299779927992
                          123     1                   1           41334 1             21232      3 3      1233          2  2 13 222 13   7  12   2  7
                          322     1                   1           3     2              3 3       3 2      4112                  3 1 2
insertions                  9     2                   6                                               4       4
deletions                         6                   6             11                                                         4 2.3
position                          .                                100    .  110    .  120    .  130        .      140    .  150    .  160    .  170
```

```
Intermediate             •
unknown              173 LG...................AA................H...................................................................................... 177*
unknown              269 L-...................--................-skfelsnkhhhkshatnhrrddndddededddddedededddddeeeedddvdenvevklhgrnktssnnknnnnknhnsninynnh 353
unknown              266 L-...................--................-skfemakgkkgkvkargkakapvnglstcanpphgnnsqinhphasaphsnapypsraistrgidstgkkrihaasagctidrg 350
unknown              266 L-...................--................-skfemakgkkgrakargrvkpppggsranhgganrgganhsgenrcganrgsanhssaglananranesaphssapdaqrapst 350
unknown              265 L-...................--................-skfdrsktrsgkngvnksgsgsgsakngsvkngsvkngsvkngsvkngsvkngsvkngsvkngsvkngsikngsikngsikngs 349
unknown              265 L-...................--................-skfdrskmrppkngggsgsgsgnvsgkggggsgsgnvsskggggsgkggsgsasrksgsgggsgsskgrsgsggasgtvsrksg 349
unknown              264 L-...................--................-skfdrskarasksgsiksadiktgsnkngsnksgsnksgatnkssanksdtsksrnsnrsgasksgtsqngtsnrsggsksgns 348
unknown              323 LG...................SIsiltsdtnnskstfshN...................................................................................... 343
unknown              274 LG...................SIsiltsdpnnlkttfshN...................................................................................... 294
unknown              333 LG...................PM................Nippadpeaqlgsrplhrhn................................................................... 356
unknown              313 LGriqpandrdrakyanenglAA................H...................................................................................... 336
unknown              313 LGriqpandrdrakyanenglAA................H...................................................................................... 336
unknown              313 LGriqpandrdrakyanenglAA................H...................................................................................... 336
unknown              318 LGriqpandrdrakyanenglAA................H...................................................................................... 341
unknown              273 LGrmkgdgeggseep......GA................H...................................................................................... 290
position                                      .
```

```
Background                •
consensus            173 LG...................AA................H...................................................................................... 177*
PFF0750w             269 L-...................--................-skfelsnkhhhkshatnhrrddndddededddddedededddddeeeedddvdenvevklhgrnktssnnknnnnknhnsninynnh 353
PKH_113350           266 L-...................--................-skfemakgkkgkvkargkakapvnglstcanpphgnnsqinhphasaphsnapypsraistrgidstgkkrihaasagctidrg 350
PVX_113910           266 L-...................--................-skfemakgkkgrakargrvkpppggsranhgganrgganhsgenrcganrgsanhssaglananranesaphssapdaqrapst 350
PBANKA_123020        265 L-...................--................-skfdrsktrsgkngvnksgsgsgsakngsvkngsvkngsvkngsvkngsvkngsvkngsvkngsvkngsikngsikngsikngs 349
PY06538              265 L-...................--................-skfdrskmrppkngggsgsgsgnvsgkggggsgsgnvsskggggsgkggsgsasrksgsgggsgsskgrsgsggasgtvsrksg 349
PCHAS_123090         264 L-...................--................-skfdrskarasksgsiksadiktgsnkngsnksgsnksgatnkssanksdtsksrnsnrsgasksgtsqngtsnrsggsksgns 348
TA09960              323 LG...................SIsiltsdtnnskstfshN...................................................................................... 343
TP04_0791            274 LG...................SIsiltsdpnnlkttfshN...................................................................................... 294
BBOV_III008880       333 LG...................PM................Nippadpeaqlgsrplhrhn................................................................... 356
TGGT1_118720         313 LGriqpandrdrakyanenglAA................H...................................................................................... 336
TGME49_029020        313 LGriqpandrdrakyanenglAA................H...................................................................................... 336
TGVEG_026470         313 LGriqpandrdrakyanenglAA................H...................................................................................... 336
NCLIV_030060         318 LGriqpandrdrakyanenglAA................H...................................................................................... 341
ETH_00023650         273 LGrmkgdgeggseep......GA................H...................................................................................... 290
foreground (196):        LG                   AK                H                                                                                       15.8
                         F                    SR                E
                                               T
wt_res_freqs (61):       67                   22                1
                         1                    22                1
                                               1
insertions
deletions                12                   15                1                                                                                       11.5
```

```
Foreground
consensus            173 LG...................AA................H...................................................................................... 177*
PFF0750w             269 L-...................--................-skfelsnkhhhkshatnhrrddndddededddddedededddddeeeedddvdenvevklhgrnktssnnknnnnknhnsninynnh 353
PKH_113350           266 L-...................--................-skfemakgkkgkvkargkakapvnglstcanpphgnnsqinhphasaphsnapypsraistrgidstgkkrihaasagctidrg 350
PVX_113910           266 L-...................--................-skfemakgkkgrakargrvkpppggsranhgganrgganhsgenrcganrgsanhssaglananranesaphssapdaqrapst 350
PBANKA_123020        265 L-...................--................-skfdrsktrsgkngvnksgsgsgsakngsvkngsvkngsvkngsvkngsvkngsvkngsvkngsvkngsikngsikngsikngs 349
PY06538              265 L-...................--................-skfdrskmrppkngggsgsgsgnvsgkggggsgsgnvsskggggsgkggsgsasrksgsgggsgsskgrsgsggasgtvsrksg 349
PCHAS_123090         264 L-...................--................-skfdrskarasksgsiksadiktgsnkngsnksgsnksgatnkssanksdtsksrnsnrsgasksgtsqngtsnrsggsksgns 348
TA09960              323 LG...................SIsiltsdtnnskstfshN...................................................................................... 343
TP04_0791            274 LG...................SIsiltsdpnnlkttfshN...................................................................................... 294
BBOV_III008880       333 LG...................PM................Nippadpeaqlgsrplhrhn................................................................... 356
TGGT1_118720         313 LGriqpandrdrakyanenglAA................H...................................................................................... 336
TGME49_029020        313 LGriqpandrdrakyanenglAA................H...................................................................................... 336
TGVEG_026470         313 LGriqpandrdrakyanenglAA................H...................................................................................... 336
NCLIV_030060         318 LGriqpandrdrakyanenglAA................H...................................................................................... 341
ETH_00023650         273 LGrmkgdgeggseep......GA................H...................................................................................... 290
foreground (15):         LG                   GA                N                                                                                       1.3
                                              SI                H
                                              A
wt_res_freqs (4):        96                   13                2
                                              11                3
                                              2
insertions                3                    1                4
deletions                 4                   44                4                                                                                       2.3
position                                      .
```

```
Intermediate
unknown        178 ............................................................................................................................
unknown        354 nwsrrknkmkkkkkkkkkkkeqdddyynkdfq............................................................................................ 385
unknown        351 raegnggnrsdqgngnsnnrkkknnkergkethgerteegekytqrkrerarmrgieeddyynnkdfq........................................................ 418
unknown        351 rgihsvrssgdgggsavraacttdggtahgsgangsgdgqgtpcnrnrkskqrevaagrgkatarekaatrenaatqenaatqnaatqnaatqnagtqenaaaqnaaaqerarlrgieeddyys 474
unknown        350 ikngsgrcsegrrsddaymgdeafekdyesfndrdfq....................................................................................... 386
unknown        350 sgkgrsggasgsgggggtvrrsdeaymgdemfeknyesfndrdfq............................................................................... 394
unknown        349 ksrtsksgtsqngtstksngsgrgserrssddaymgdepfekeyesfndrdfq....................................................................... 401
unknown        344 ............................................................................................................................
unknown        295 ............................................................................................................................
unknown        357 ............................................................................................................................
unknown        337 ............................................................................................................................
unknown        337 ............................................................................................................................
unknown        337 ............................................................................................................................
unknown        342 ............................................................................................................................
unknown        291 ............................................................................................................................
position

Background
consensus      178 ............................................................................................................................
PFF0750w       354 nwsrrknkmkkkkkkkkkkkeqdddyynkdfq............................................................................................ 385
PKH_113350     351 raegnggnrsdqgngnsnnrkkknnkergkethgerteegekytqrkrerarmrgieeddyynnkdfq........................................................ 418
PVX_113910     351 rgihsvrssgdgggsavraacttdggtahgsgangsgdgqgtpcnrnrkskqrevaagrgkatarekaatrenaatqenaatqnaatqnaatqnagtqenaaaqnaaaqerarlrgieeddyys 474
PBANKA_123020  350 ikngsgrcsegrrsddaymgdeafekdyesfndrdfq....................................................................................... 386
PY06538        350 sgkgrsggasgsgggggtvrrsdeaymgdemfeknyesfndrdfq............................................................................... 394
PCHAS_123090   349 ksrtsksgtsqngtstksngsgrgserrssddaymgdepfekeyesfndrdfq....................................................................... 401
TA09960        344 ............................................................................................................................
TP04_0791      295 ............................................................................................................................
BBOV_III008880 357 ............................................................................................................................
TGGT1_118720   337 ............................................................................................................................
TGME49_029020  337 ............................................................................................................................
TGVEG_026470   337 ............................................................................................................................
NCLIV_030060   342 ............................................................................................................................
ETH_00023650   291 ............................................................................................................................
insertions
deletions                                                                                                                                        11.5

Foreground
consensus      178 ............................................................................................................................
PFF0750w       354 nwsrrknkmkkkkkkkkkkkeqdddyynkdfq............................................................................................ 385
PKH_113350     351 raegnggnrsdqgngnsnnrkkknnkergkethgerteegekytqrkrerarmrgieeddyynnkdfq........................................................ 418
PVX_113910     351 rgihsvrssgdgggsavraacttdggtahgsgangsgdgqgtpcnrnrkskqrevaagrgkatarekaatrenaatqenaatqnaatqnaatqnagtqenaaaqnaaaqerarlrgieeddyys 474
PBANKA_123020  350 ikngsgrcsegrrsddaymgdeafekdyesfndrdfq....................................................................................... 386
PY06538        350 sgkgrsggasgsgggggtvrrsdeaymgdemfeknyesfndrdfq............................................................................... 394
PCHAS_123090   349 ksrtsksgtsqngtstksngsgrgserrssddaymgdepfekeyesfndrdfq....................................................................... 401
TA09960        344 ............................................................................................................................
TP04_0791      295 ............................................................................................................................
BBOV_III008880 357 ............................................................................................................................
TGGT1_118720   337 ............................................................................................................................
TGME49_029020  337 ............................................................................................................................
TGVEG_026470   337 ............................................................................................................................
NCLIV_030060   342 ............................................................................................................................
ETH_00023650   291 ............................................................................................................................
insertions
deletions                                                                                                                                        2.3
position
```

```
Intermediate
unknown              178 .....YGLNVDIWSAACIICEMIIGRPLFHGVQEFQVIIRIVNSLGTPTEDEWENCSRSQHY.......SHXDFSNH......KIQNRKDALQ.....KIDEDGIDLLVKMLKYNPHDRITAAD 278*
unknown              386 .....YGLNVDIWSAACIICELIIGRPLFKGVTEFDLIIRIVNSLGKPNKDELEFFSNSRFYpl.....KE-DFFNV......HIKNKKDALNvitngRIDELGIDLLVKMLKYNPNDRITAAD 492
unknown              419 .....YGLNVDIWSAACIICELIIGRPLFRGVTEFDLIIRIVNSLGKPNNDELEFFSDSRFYpl.....KE-DFFNV......NIKNKKDALNvitngRIDELGIDLLVKMLKYNPNDRITAAD 525
unknown              475 nkdfqYGLNVDIWSAACIICELIIGRPLFRGVTEFDLIIRIVNSLGKPNSDELEFFSDSRFYpl.....KE-DFFNV......NIKNRKDALNvitngRIDELGIDLLVKMLKYNPNDRITAAD 586
unknown              387 .....YGLNVDIWSAACIICELIIGKPLFRGVTEFDLIIRIVNSLGKPNNDELEYFSDSRFYpf.....KD-DFFNI......KIKNRKDALNvitngKIDELGIDLLVKMLKYNPNDRITAAD 493
unknown              395 .....YGLNVDIWSAACIICELIIGKPLFRGVTEFDLIIRIVNSLGKPNKNELEYFSDSRFYpf.....KD-DFFNI......KIKDRKDALNvitngKIDELGIDLLVKMLKYNPNDRITAAD 501
unknown              402 .....YGLNVDIWSAACIICELIIGKPLFRGVTEFDLIIRIVNSLGKPNNDELEYFSDSRFYpf.....KD-DFFNI......KIKNRKDALNvitngKIDELGIDLLVKMLKYNPNDRITAAD 508
unknown              344 .....YGINVDMWSAACIIAEMITGRPLFHGNSDFQVLSRIVTILGRPTEAEWKNCSLSEHY.......PF-NGSLYqfkissKKENLRVALRg....KIDDTGLDLLLSMLEYNPHKRISAQV 450
unknown              295 .....YGINVDMWSAACIIAEMITGRPLFHGNSDFQVLSRIVTILGRPTEAEWKNCSLSEHY.......PF-NGSLYqfkissKKENLRVALKg....RMDDTGLDLLLSMLEYNPHKRISAQV 401
unknown              357 .....YGINADIWSAACIIAELVTGFPLFHGNSDFQVLTRIVTTLGRPTEDEWRNCSRSEHY.......PF-NGSLYnvhvedKKERLRSVLRg....KMDDVGLDLLLSLLQYNPHKRLSALE 463
unknown              337 .....YGLGVDLWSAGCILAEMIRGTPLFKGTQEFEVLIRVTKVLGTPTEEEWTNCCRLQHYpfrnlseSH-FFSEHd.....KRQNLNAVLQg....KLDLDGLDLLARMLDYNPHRRITAAE 445
unknown              337 .....YGLGVDLWSAGCILAEMIRGTPLFKGTQEFEVLIRVTKVLGTPTEEEWTNCCRLQHYpfrnlseSH-FFSEHd.....KRQNLNAVLQg....KLDLDGLDLLARMLDYNPHRRITAAE 445
unknown              337 .....YGLGVDLWSAGCILAEMIRGTPLFKGTQEFEVLIRVTKVLGTPTEEEWTNCCRLQHYpfrnlseSH-FFSEHd.....KRQNLNAVLQg....KLDLDGLDLLARMLDYNPHRRITAAE 445
unknown              342 .....YGLGVDLWSAGCIIAEMIRGTPLFKGTQEFEVLIRVTKVLGTPTEEEWTNCCRLQHYpfrnlseSH-FFSEHd.....KRQNLNAVLQg....KLDLDGLDLLARMLDYNPHRRITAAE 450
unknown              291 .....YGLGADIWGAGCIIAEMFRGIPLFKGTQEFEVLIRVTKILGTPTEAEW---------.......--------......----------.....-------------------------- 338
position                  180  .  190  .  200  .  210  .  220  .  230          .  240          .  250          .  260  .  270  .

Background
consensus            178 .....YGLNVDIWSAACIICEMIIGRPLFHGVQEFQVIIRIVNSLGTPTEDEWENCSRSQHY.......SHXDFSNH......KIQNRKDALQ.....KIDEDGIDLLVKMLKYNPHDRITAAD 278*
PFF0750w             386 .....YGLNVDIWSAACIICELIIGRPLFKGVTEFDLIIRIVNSLGKPNKDELEFFSNSRFYpl.....KE-DFFNV......HIKNKKDALNvitngRIDELGIDLLVKMLKYNPNDRITAAD 492
PKH_113350           419 .....YGLNVDIWSAACIICELIIGRPLFRGVTEFDLIIRIVNSLGKPNNDELEFFSDSRFYpl.....KE-DFFNV......NIKNKKDALNvitngRIDELGIDLLVKMLKYNPNDRITAAD 525
PVX_113910           475 nkdfqYGLNVDIWSAACIICELIIGRPLFRGVTEFDLIIRIVNSLGKPNSDELEFFSDSRFYpl.....KE-DFFNV......NIKNRKDALNvitngRIDELGIDLLVKMLKYNPNDRITAAD 586
PBANKA_123020        387 .....YGLNVDIWSAACIICELIIGKPLFRGVTEFDLIIRIVNSLGKPNNDELEYFSDSRFYpf.....KD-DFFNI......KIKNRKDALNvitngKIDELGIDLLVKMLKYNPNDRITAAD 493
PY06538              395 .....YGLNVDIWSAACIICELIIGKPLFRGVTEFDLIIRIVNSLGKPNKNELEYFSDSRFYpf.....KD-DFFNI......KIKDRKDALNvitngKIDELGIDLLVKMLKYNPNDRITAAD 501
PCHAS_123090         402 .....YGLNVDIWSAACIICELIIGKPLFRGVTEFDLIIRIVNSLGKPNNDELEYFSDSRFYpf.....KD-DFFNI......KIKNRKDALNvitngKIDELGIDLLVKMLKYNPNDRITAAD 508
TA09960              344 .....YGINVDMWSAACIIAEMITGRPLFHGNSDFQVLSRIVTILGRPTEAEWKNCSLSEHY.......PF-NGSLYqfkissKKENLRVALRg....KIDDTGLDLLLSMLEYNPHKRISAQV 450
TP04_0791            295 .....YGINVDMWSAACIIAEMITGRPLFHGNSDFQVLSRIVTILGRPTEAEWKNCSLSEHY.......PF-NGSLYqfkissKKENLRVALKg....RMDDTGLDLLLSMLEYNPHKRISAQV 401
BBOV_III008880       357 .....YGINADIWSAACIIAELVTGFPLFHGNSDFQVLTRIVTTLGRPTEDEWRNCSRSEHY.......PF-NGSLYnvhvedKKERLRSVLRg....KMDDVGLDLLLSLLQYNPHKRLSALE 463
TGGT1_118720         337 .....YGLGVDLWSAGCILAEMIRGTPLFKGTQEFEVLIRVTKVLGTPTEEEWTNCCRLQHYpfrnlseSH-FFSEHd.....KRQNLNAVLQg....KLDLDGLDLLARMLDYNPHRRITAAE 445
TGME49_029020        337 .....YGLGVDLWSAGCILAEMIRGTPLFKGTQEFEVLIRVTKVLGTPTEEEWTNCCRLQHYpfrnlseSH-FFSEHd.....KRQNLNAVLQg....KLDLDGLDLLARMLDYNPHRRITAAE 445
TGVEG_026470         337 .....YGLGVDLWSAGCILAEMIRGTPLFKGTQEFEVLIRVTKVLGTPTEEEWTNCCRLQHYpfrnlseSH-FFSEHd.....KRQNLNAVLQg....KLDLDGLDLLARMLDYNPHRRITAAE 445
NCLIV_030060         342 .....YGLGVDLWSAGCIIAEMIRGTPLFKGTQEFEVLIRVTKVLGTPTEEEWTNCCRLQHYpfrnlseSH-FFSEHd.....KRQNLNAVLQg....KLDLDGLDLLARMLDYNPHRRITAAE 450
ETH_00023650         291 .....YGLGADIWGAGCIIAEMFRGIPLFKGTQEFEVLIRVTKILGTPTEAEW---------.......--------......----------.....-------------------------- 338
foreground (196):             YSTAVDVWAVGCVFAEMVTGKPIFPGNSDIDQLFKIFKVLGTPSEDSWPGVSKLPNY       NK SFPKW      PPNNLKEVFP     NIDDDGIDLINKMLQYNPSKRISAKD 15.8
                               TSSI M SI  IMG LLNRRALLQ D E E IDR  RLC S TDET  DLTS  DF       K  T  EF      K RD RDLVS     RLSEEALE LDRL TLD ER LT EE
                               GPG  L GA   L   ILKE     E Q    EL  EIF   NPKV   I N  G        S  K   Y        KP SKILK     K GP      S   V          Q
wt_res_freqs (61):            923449191499145941232717381424477238611586814117743226614       11 14521      2211611133     11211416811338132814963722
                               2113 3 61  711 421211611 2 5 1 111  221 1 3121  1111  11       1  1  11      1 11 21111     13211231 6114 124 11 14 12
                               221  1 11   1   1111     1 1    11  111   2111   1 1  1        1  1   1        11 11111     1 11      1   1          1
insertions                           1        121      1 5 5             1     1      1       1119  11       2221  132     249
deletions                     112111211122222222222232222223223322224444565545556655556       91511166      7767666667     78655555555555556666666666 11.5

Foreground
consensus            178 .....YGLNVDIWSAACIICEMIIGRPLFHGVQEFQVIIRIVNSLGTPTEDEWENCSRSQHY.......SHXDFSNH......KIQNRKDALQ.....KIDEDGIDLLVKMLKYNPHDRITAAD 278*
PFF0750w             386 .....YGLNVDIWSAACIICELIIGRPLFKGVTEFDLIIRIVNSLGKPNKDELEFFSNSRFYpl.....KE-DFFNV......HIKNKKDALNvitngRIDELGIDLLVKMLKYNPNDRITAAD 492
PKH_113350           419 .....YGLNVDIWSAACIICELIIGRPLFRGVTEFDLIIRIVNSLGKPNNDELEFFSDSRFYpl.....KE-DFFNV......NIKNKKDALNvitngRIDELGIDLLVKMLKYNPNDRITAAD 525
PVX_113910           475 nkdfqYGLNVDIWSAACIICELIIGRPLFRGVTEFDLIIRIVNSLGKPNSDELEFFSDSRFYpl.....KE-DFFNV......NIKNRKDALNvitngRIDELGIDLLVKMLKYNPNDRITAAD 586
PBANKA_123020        387 .....YGLNVDIWSAACIICELIIGKPLFRGVTEFDLIIRIVNSLGKPNNDELEYFSDSRFYpf.....KD-DFFNI......KIKNRKDALNvitngKIDELGIDLLVKMLKYNPNDRITAAD 493
PY06538              395 .....YGLNVDIWSAACIICELIIGKPLFRGVTEFDLIIRIVNSLGKPNKNELEYFSDSRFYpf.....KD-DFFNI......KIKDRKDALNvitngKIDELGIDLLVKMLKYNPNDRITAAD 501
PCHAS_123090         402 .....YGLNVDIWSAACIICELIIGKPLFRGVTEFDLIIRIVNSLGKPNNDELEYFSDSRFYpf.....KD-DFFNI......KIKNRKDALNvitngKIDELGIDLLVKMLKYNPNDRITAAD 508
TA09960              344 .....YGINVDMWSAACIIAEMITGRPLFHGNSDFQVLSRIVTILGRPTEAEWKNCSLSEHY.......PF-NGSLYqfkissKKENLRVALRg....KIDDTGLDLLLSMLEYNPHKRISAQV 450
TP04_0791            295 .....YGINVDMWSAACIIAEMITGRPLFHGNSDFQVLSRIVTILGRPTEAEWKNCSLSEHY.......PF-NGSLYqfkissKKENLRVALKg....RMDDTGLDLLLSMLEYNPHKRISAQV 401
BBOV_III008880       357 .....YGINADIWSAACIIAELVTGFPLFHGNSDFQVLTRIVTTLGRPTEDEWRNCSRSEHY.......PF-NGSLYnvhvedKKERLRSVLRg....KMDDVGLDLLLSLLQYNPHKRLSALE 463
TGGT1_118720         337 .....YGLGVDLWSAGCILAEMIRGTPLFKGTQEFEVLIRVTKVLGTPTEEEWTNCCRLQHYpfrnlseSH-FFSEHd.....KRQNLNAVLQg....KLDLDGLDLLARMLDYNPHRRITAAE 445
TGME49_029020        337 .....YGLGVDLWSAGCILAEMIRGTPLFKGTQEFEVLIRVTKVLGTPTEEEWTNCCRLQHYpfrnlseSH-FFSEHd.....KRQNLNAVLQg....KLDLDGLDLLARMLDYNPHRRITAAE 445
TGVEG_026470         337 .....YGLGVDLWSAGCILAEMIRGTPLFKGTQEFEVLIRVTKVLGTPTEEEWTNCCRLQHYpfrnlseSH-FFSEHd.....KRQNLNAVLQg....KLDLDGLDLLARMLDYNPHRRITAAE 445
NCLIV_030060         342 .....YGLGVDLWSAGCIIAEMIRGTPLFKGTQEFEVLIRVTKVLGTPTEEEWTNCCRLQHYpfrnlseSH-FFSEHd.....KRQNLNAVLQg....KLDLDGLDLLARMLDYNPHRRITAAE 450
ETH_00023650         291 .....YGLGADIWGAGCIIAEMFRGIPLFKGTQEFEVLIRVTKILGTPTEAEW---------.......--------......----------.....-------------------------- 338
foreground (15):              YGLNADLWGAACIICELITGTPLFKGTSDFDVIIRVVTSLGTPNNAEWTNCSDSEHY       SD NGSNH      KKENKNAALN     KIDDTGIDLLASMLDYNPHDRISAAD 1.3
                                IG  M   G  LA  FI I   H VQ  QLLS ITKI  R  KE LKFFCLLRF        PF F FLI      NIK LRVV R     RM LL     LR  K   NR    QV
                                                R R   R NT  E      NV  K   D  EY  R Q         KH D  EV       RQ RKD  Q      L ED     VK  E    K     E
wt_res_freqs (4):             997629291969983947292999493229363793623993931296255636258       21 22532      7227122583     64821838882278288853872863
                                23  1   3  16  13 1   2 33  2361 6332  2  12 311321233        22 2 321      133 5213 1     21 23     22  3   32    11
                                                3 3   3 23  3      32  3   4  31  3 2         32 3  21       22 233  2      2 32     33  1    2     3
insertions                                                                          6              4               8
deletions                                                                   666666666       66966666      6666666666     66666666666666666666666666 2.3
position                      180  .  190  .  200  .  210  .  220  .  230          .  240          .  250          .  260  .  270  .
```

Intermediate

unknown	279	ALSHQWF	285*
unknown	493	ALSHPWF	499
unknown	526	ALSHPWF	532
unknown	587	ALSHPWF	593
unknown	494	ALSHPWF	500
unknown	502	ALSHPWF	508
unknown	509	ALSHPWF	515
unknown	451	ALSHRWF	457
unknown	402	ALSHKWF	408
unknown	464	ALSHPWF	470
unknown	446	ALSHQWF	452
unknown	446	ALSHQWF	452
unknown	446	ALSHQWF	452
unknown	451	ALSHQWF	457
unknown	339	-------	
position		.	

Background

consensus	279	ALSHQWF	285*
PFF0750w	493	ALSHPWF	499
PKH_113350	526	ALSHPWF	532
PVX_113910	587	ALSHPWF	593
PBANKA_123020	494	ALSHPWF	500
PY06538	502	ALSHPWF	508
PCHAS_123090	509	ALSHPWF	515
TA09960	451	ALSHRWF	457
TP04_0791	402	ALSHKWF	408
BBOV_III008880	464	ALSHPWF	470
TGGT1_118720	446	ALSHQWF	452
TGME49_029020	446	ALSHQWF	452
TGVEG_026470	446	ALSHQWF	452
NCLIV_030060	451	ALSHQWF	457
ETH_00023650	339	-------	
foreground (196):		ALNHPWF / Q FL / E Y	15.8
wt_res_freqs (61):		7716527 / 1 11 / 1 4	
insertions			
deletions		6667881	11.5

Foreground

consensus	279	ALSHQWF	285*
PFF0750w	493	ALSHPWF	499
PKH_113350	526	ALSHPWF	532
PVX_113910	587	ALSHPWF	593
PBANKA_123020	494	ALSHPWF	500
PY06538	502	ALSHPWF	508
PCHAS_123090	509	ALSHPWF	515
TA09960	451	ALSHRWF	457
TP04_0791	402	ALSHKWF	408
BBOV_III008880	464	ALSHPWF	470
TGGT1_118720	446	ALSHQWF	452
TGME49_029020	446	ALSHQWF	452
TGVEG_026470	446	ALSHQWF	452
NCLIV_030060	451	ALSHQWF	457
ETH_00023650	339	-------	
foreground (15):		ALSHQWF / P	1.3
wt_res_freqs (4):		8888288 / 4	
insertions			
deletions		6666666	2.3
position		.	
