# Supplementary material for: Structural and evolutionary divergence of eukaryotic protein kinases in Apicomplexa
Source: BMC Evol Biol. 2011 Nov 2;11:321. doi: 10.1186/1471-2148-11-321 (PMC3239843; doi:10.1186/1471-2148-11-321)
Supplement: Additional file 5 — CDK-SCTTLRE subfamily CHAIN alignment versus the CDC2 subfamily. Colorized sequence alignment and partition generated by the CHAIN program, comparing the apicomplexan-specific subfamily of CDKs to eukaryotic CDC2 subfamily members. [file 1471-2148-11-321-S5.PDF]

Apicomplexan CDK subfamily vs. eukaryotic CDC2

```
Intermediate                  ●●
unknown                 1 XXXVERLGGGTYGDVYKAV......................E..........................................YYY.....................................  23*
unknown                24 ----EKLGGGTYGDVYKGKvikynnnnnnndlyqntnylpyD..........................................YYT.....................................  64
unknown                24 ----EKLGGGTYGDVYKGKvikynnnnnndlyqntnylpy.D..........................................YYT.....................................  63
unknown                24 ----EKLGGGTYGDVYKGKvikysnsssndlyqntnylpy.D..........................................YYT.....................................  63
unknown                24 ----EKLGGGTYGDVYKGK......................V..........................................VKY.....................................  42
unknown                24 ----EKLGGGTYGDVYKGK......................V..........................................VKY.....................................  42
unknown                24 ----EKLGGGTYGDVYKGK......................V..........................................VKY.....................................  42
unknown               131 -------------------......................-..........................................---.....................................
unknown               131 -------------------......................-..........................................---.....................................
unknown               127 -------------------......................-..........................................---.....................................
unknown                34 ---VERLGGGTYGDVYKAV......................EeeeeendrgsarardrpgistaeataeatngedasakegsekKTK.....................................  95
unknown                34 ---VERLGGGTYGDVYKAV......................EeeeeendrgsarardrpgistaeataeatngedasakegsekKTK.....................................  95
unknown                34 ---VERLGGGTYGDVYKAV......................EeeeeendrgsarardrpgistaeataeatngedasakegsekKTK.....................................  95
unknown                34 ---VERLGGGTYGDVYKAV......................E..........................................EEDeenqrgassslssasrdgasdggrgnveetpekegea  90
unknown                34 ---VERLGGGTYGDVYRAV......................E..........................................EPEdgvgg................................  58
position                      .   10    .                         20

Background                ●     ●● ●●●● ●●●●                      ●                                            ●
consensus               1 XXXVERLGGGTYGDVYKAV......................E..........................................YYY.....................................  23*
PFF0750w               24 ----EKLGGGTYGDVYKGKvikynnnnnnndlyqntnylpyD..........................................YYT.....................................  64
PKH_113350             24 ----EKLGGGTYGDVYKGKvikynnnnnndlyqntnylpy.D..........................................YYT.....................................  63
PVX_113910             24 ----EKLGGGTYGDVYKGKvikysnsssndlyqntnylpy.D..........................................YYT.....................................  63
PBANKA_123020          24 ----EKLGGGTYGDVYKGK......................V..........................................VKY.....................................  42
PY06538                24 ----EKLGGGTYGDVYKGK......................V..........................................VKY.....................................  42
PCHAS_123090           24 ----EKLGGGTYGDVYKGK......................V..........................................VKY.....................................  42
TA09960               131 -------------------......................-..........................................---.....................................
TP04_0791             131 -------------------......................-..........................................---.....................................
BBOV_III008880        127 -------------------......................-..........................................---.....................................
TGGT1_118720           34 ---VERLGGGTYGDVYKAV......................EeeeeendrgsarardrpgistaeataeatngedasakegsekKTK.....................................  95
TGME49_029020          34 ---VERLGGGTYGDVYKAV......................EeeeeendrgsarardrpgistaeataeatngedasakegsekKTK.....................................  95
TGVEG_026470           34 ---VERLGGGTYGDVYKAV......................EeeeeendrgsarardrpgistaeataeatngedasakegsekKTK.....................................  95
NCLIV_030060           34 ---VERLGGGTYGDVYKAV......................E..........................................EEDeenqrgassslssasrdgasdggrgnveetpekegea  90
ETH_00023650           34 ---VERLGGGTYGDVYRAV......................E..........................................EPEdgvgg................................  58
foreground (63):          YEKIEKVGEGTYGVVYKAQ                      N                                          KQT                                       6.0
                          FTLL  L S SF I FRGT                      D                                          TE
                           I    I   A  K   CR                                                                  R
wt_res_freqs (23):        6132341858579285551                      1                                          225
                          1111  3 1 11 1 1211                      4                                          11
                           1    4   1  1   11                                                                  1
insertions                                  1                                                                 1 3
deletions                 1111119997777766666                      9                                          766                                       9.1

Foreground                          ●    ●
consensus               1 XXXVERLGGGTYGDVYKAV......................E..........................................YYY.....................................  23*
PFF0750w               24 ----EKLGGGTYGDVYKGKvikynnnnnnndlyqntnylpyD..........................................YYT.....................................  64
PKH_113350             24 ----EKLGGGTYGDVYKGKvikynnnnnndlyqntnylpy.D..........................................YYT.....................................  63
PVX_113910             24 ----EKLGGGTYGDVYKGKvikysnsssndlyqntnylpy.D..........................................YYT.....................................  63
PBANKA_123020          24 ----EKLGGGTYGDVYKGK......................V..........................................VKY.....................................  42
PY06538                24 ----EKLGGGTYGDVYKGK......................V..........................................VKY.....................................  42
PCHAS_123090           24 ----EKLGGGTYGDVYKGK......................V..........................................VKY.....................................  42
TA09960               131 -------------------......................-..........................................---.....................................
TP04_0791             131 -------------------......................-..........................................---.....................................
BBOV_III008880        127 -------------------......................-..........................................---.....................................
TGGT1_118720           34 ---VERLGGGTYGDVYKAV......................EeeeeendrgsarardrpgistaeataeatngedasakegsekKTK.....................................  95
TGME49_029020          34 ---VERLGGGTYGDVYKAV......................EeeeeendrgsarardrpgistaeataeatngedasakegsekKTK.....................................  95
TGVEG_026470           34 ---VERLGGGTYGDVYKAV......................EeeeeendrgsarardrpgistaeataeatngedasakegsekKTK.....................................  95
NCLIV_030060           34 ---VERLGGGTYGDVYKAV......................E..........................................EEDeenqrgassslssasrdgasdggrgnveetpekegea  90
ETH_00023650           34 ---VERLGGGTYGDVYRAV......................E..........................................EPEdgvgg................................  58
foreground (15):             VEKLGGGTYGDVYKGK                      D                                          ETT                                       0.9
                               R          RAV                      V                                          VPY
                                                                   E                                          YYK
wt_res_freqs (3):            3737777777777633                      1                                          111
                               3          133                      1                                          111
                                                                   3                                          111
insertions                                  2                      2                                            1
deletions                 9996222222222222222                      2                                          222                                       2.3
position                      .   10    .                         20
```

```
Intermediate
unknown       24 .......GN...........................FY.......AIKYFKDDT...N...EEGISCTTVRELSCMKNC...........H.HPNIVRMIDV.....................  66*
unknown       65 .......DE...........................FVffrknvyAIKFFRDDLrtiN...EEGISCTTLRELSCLKNIg..........R.HPNILRLIDV.....................  118
unknown       64 .......DE...........................FVffrkniyAIKFFRDDL...KtinEEGISCTTLRELSCLKNIg..........R.HPNILRLIDV.....................  117
unknown       64 .......DE...........................FVffrkniyAIKFFRDDL...KtinDEGISCTTLRELSCLKNIg..........R.HPNILRLIDV.....................  117
unknown       43 .......NNnnsdlyqntnylpydyytdefiffrknIY.......AIKFFKDDLrtiN...EEGISCTTLRELSCLKNIg..........R.HPNILRLIDVtidrqkgiseyinrqilqhyt  137
unknown       43 .......NNnnndlyqntnylpydyytddfiffrknIY.......AIKFFKDDLrtiN...EEGISCTTLRELSCLKNIg..........R.HPNILRLIDVtidrqkgiseyinrqilqhyt  137
unknown       43 .......NNnsdlyqntnylpydyytdefiffrkn.IY.......AIKFFKDDLrtiN...EEGISCTTLRELSCLKNIg..........R.HPNILRLIDV.....................  115
unknown      131 .......--...........................-Y.......AIKYFKDDSiqlL...DEGFTSGTIRELSIMKSV...........SgHPNIVKLVDI.....................  174
unknown      131 .......--...........................-Y.......AIKYFKDDSiqlL...DEGFTSGTIRELSIMKSV...........SgHPNIVKLVDI.....................  174
unknown      127 .......--...........................VY.......AVKYFKDDI...VhimEEGISPGTLRELSIMKVCrssgkynvqscnD.HPNIVKLVDV.....................  182
unknown       96 .......GN...........................FY.......AVKYYKDET...RtimEEGISCTTVRELSAVAGCg..........H.HPNVVRMESL.....................  142
unknown       96 .......GN...........................FY.......AVKYYKDET...RtimEEGISCTTVRELSAVAGCg..........H.HPNVVRMESL.....................  142
unknown       96 .......GN...........................FY.......AVKYYKDET...RtimEEGISCTTVRELSAVAGCg..........H.HPNVVRMESL.....................  142
unknown       91 kekqegkGN...........................LY.......AVKYYKDET...RtimEEGISCTTVRELSAVAGCg..........H.HPNVVRMESL.....................  144
unknown       59 .......GE...........................TF.......AVKYYVDETrtaT...EDGLGCTTIRELSTISSCg..........T.HPNLVRMRYL.....................  105
position                .                                     30     .        40    .    50     .                  60     .

Background
consensus     24 .......GN...........................FY.......AIKYFKDDT...N...EEGISCTTVRELSCMKNC...........H.HPNIVRMIDV.....................  66*
PFF0750w      65 .......DE...........................FVffrknvyAIKFFRDDLrtiN...EEGISCTTLRELSCLKNIg..........R.HPNILRLIDV.....................  118
PKH_113350    64 .......DE...........................FVffrkniyAIKFFRDDL...KtinEEGISCTTLRELSCLKNIg..........R.HPNILRLIDV.....................  117
PVX_113910    64 .......DE...........................FVffrkniyAIKFFRDDL...KtinDEGISCTTLRELSCLKNIg..........R.HPNILRLIDV.....................  117
PBANKA_123020 43 .......NNnnsdlyqntnylpydyytdefiffrknIY.......AIKFFKDDLrtiN...EEGISCTTLRELSCLKNIg..........R.HPNILRLIDVtidrqkgiseyinrqilqhyt  137
PY06538       43 .......NNnnndlyqntnylpydyytddfiffrknIY.......AIKFFKDDLrtiN...EEGISCTTLRELSCLKNIg..........R.HPNILRLIDVtidrqkgiseyinrqilqhyt  137
PCHAS_123090  43 .......NNnsdlyqntnylpydyytdefiffrkn.IY.......AIKFFKDDLrtiN...EEGISCTTLRELSCLKNIg..........R.HPNILRLIDV.....................  115
TA09960      131 .......--...........................-Y.......AIKYFKDDSiqlL...DEGFTSGTIRELSIMKSV...........SgHPNIVKLVDI.....................  174
TP04_0791    131 .......--...........................-Y.......AIKYFKDDSiqlL...DEGFTSGTIRELSIMKSV...........SgHPNIVKLVDI.....................  174
BBOV_III008880 127 .......--.........................VY.......AVKYFKDDI...VhimEEGISPGTLRELSIMKVCrssgkynvqscnD.HPNIVKLVDV.....................  182
TGGT1_118720  96 .......GN...........................FY.......AVKYYKDET...RtimEEGISCTTVRELSAVAGCg..........H.HPNVVRMESL.....................  142
TGME49_029020 96 .......GN...........................FY.......AVKYYKDET...RtimEEGISCTTVRELSAVAGCg..........H.HPNVVRMESL.....................  142
TGVEG_026470  96 .......GN...........................FY.......AVKYYKDET...RtimEEGISCTTVRELSAVAGCg..........H.HPNVVRMESL.....................  142
NCLIV_030060  91 kekqegkGN...........................LY.......AVKYYKDET...RtimEEGISCTTVRELSAVAGCg..........H.HPNVVRMESL.....................  144
ETH_00023650  59 .......GE...........................TF.......AVKYYVDETrtaT...EDGLGCTTIRELSTISSCg..........T.HPNLVRMRYL.....................  105
foreground (63):        GE                          VV       AVKKVKLDQ   E   DDGVPASSIREIAVLKEL            N HPNVVKLLDV                       6.0
                        NQ                          L         M RIRFES   Q   EE ISSTTL   SL RKI            D   IIR KEI
                        KK                          I         L  M  N    D   N      A     I  S             K     S  S
wt_res_freqs (23):      32                          16       719313321   3   22836111389721843 7           2 8581637255
                        21                          1         1 142111   1   34 314424   42 211            1   721 112
                        11                          1         3  1  1    1   1      2     3  1             2    2  1
insertions                                                          17   3   61 1             4            4  1  1  4
deletions               66                          77       777634347   6   396333333333333333            3 1111111111                     9.1

Foreground
consensus     24 .......GN...........................FY.......AIKYFKDDT...N...EEGISCTTVRELSCMKNC...........H.HPNIVRMIDV.....................  66*
PFF0750w      65 .......DE...........................FVffrknvyAIKFFRDDLrtiN...EEGISCTTLRELSCLKNIg..........R.HPNILRLIDV.....................  118
PKH_113350    64 .......DE...........................FVffrkniyAIKFFRDDL...KtinEEGISCTTLRELSCLKNIg..........R.HPNILRLIDV.....................  117
PVX_113910    64 .......DE...........................FVffrkniyAIKFFRDDL...KtinDEGISCTTLRELSCLKNIg..........R.HPNILRLIDV.....................  117
PBANKA_123020 43 .......NNnnsdlyqntnylpydyytdefiffrknIY.......AIKFFKDDLrtiN...EEGISCTTLRELSCLKNIg..........R.HPNILRLIDVtidrqkgiseyinrqilqhyt  137
PY06538       43 .......NNnnndlyqntnylpydyytddfiffrknIY.......AIKFFKDDLrtiN...EEGISCTTLRELSCLKNIg..........R.HPNILRLIDVtidrqkgiseyinrqilqhyt  137
PCHAS_123090  43 .......NNnsdlyqntnylpydyytdefiffrkn.IY.......AIKFFKDDLrtiN...EEGISCTTLRELSCLKNIg..........R.HPNILRLIDV.....................  115
TA09960      131 .......--...........................-Y.......AIKYFKDDSiqlL...DEGFTSGTIRELSIMKSV...........SgHPNIVKLVDI.....................  174
TP04_0791    131 .......--...........................-Y.......AIKYFKDDSiqlL...DEGFTSGTIRELSIMKSV...........SgHPNIVKLVDI.....................  174
BBOV_III008880 127 .......--.........................VY.......AVKYFKDDI...VhimEEGISPGTLRELSIMKVCrssgkynvqscnD.HPNIVKLVDV.....................  182
TGGT1_118720  96 .......GN...........................FY.......AVKYYKDET...RtimEEGISCTTVRELSAVAGCg..........H.HPNVVRMESL.....................  142
TGME49_029020 96 .......GN...........................FY.......AVKYYKDET...RtimEEGISCTTVRELSAVAGCg..........H.HPNVVRMESL.....................  142
TGVEG_026470  96 .......GN...........................FY.......AVKYYKDET...RtimEEGISCTTVRELSAVAGCg..........H.HPNVVRMESL.....................  142
NCLIV_030060  91 kekqegkGN...........................LY.......AVKYYKDET...RtimEEGISCTTVRELSAVAGCg..........H.HPNVVRMESL.....................  144
ETH_00023650  59 .......GE...........................TF.......AVKYYVDETrtaT...EDGLGCTTIRELSTISSCg..........T.HPNLVRMRYL.....................  105
foreground (15):        GN                          TY       AVKYYRDDS   T   EEGFSCGTVRELSCVAGC            S HPNVVRLESV                       0.9
                        DE                          IV        I FFV EL   L   DD LTS  L    IMKNI            H   LLKMIYL
                        N                           FF              T    R        G   I    TLSSV            R     I   VDI
wt_res_freqs (3):       34                          17       949631961   1   789177292999932224            1 9992676224
                        12                          11        5 361 33   1   21 111  4    22633            2   1323313
                        1                           31              3    2        1  2    13121            3     6   261
insertions               2                           2              4    4                    8            1            1
deletions               22                          1                                                                                   2.3
position                .                                     30     .        40    .    50     .                  60     .
```

```
Intermediate
unknown       67 ..F....................................V............................................QQL.....................................  71*
unknown      119 ..T....................................I............................................D--.....................................  121
unknown      118 ..T....................................I............................................D--.....................................  120
unknown      118 ..T....................................I............................................D--.....................................  120
unknown      138 snY....................................H............................................H--.....................................  142
unknown      138 snY....................................H............................................H--.....................................  142
unknown      116 ..T....................................I............................................D--.....................................  118
unknown      175 ..F....................................V............................................G--.....................................  177
unknown      175 ..F....................................V............................................G--.....................................  177
unknown      183 ..YvgphpgivdmlnqqlgdalkssgtevagrkdlqffpF............................................K--.....................................  221
unknown      143 ..F....................................VdplprlarainqqrltwarnqqsalssqqfshlqeqlqkeelkpQ--.....................................  189
unknown      143 ..F....................................VdplprlarainqqrltwarnqqsalssqqfshlqeqlqkeelkpQ--.....................................  189
unknown      143 ..F....................................VdplprlarainqqrltwarnqqsalssqqfshlqeqlqkeelkpQ--.....................................  189
unknown      145 ..F....................................V............................................DPLprlaravnqqrvawarsqqsslssqqyshlqnqiqke  186
unknown      106 ..F....................................V............................................DQLprlveninqqriqwarqqhaaadresqeqlrrdlqtq  147
position                                                                                                 70

Background
consensus     67 ..F....................................V............................................QQL.....................................  71*
PFF0750w     119 ..T....................................I............................................D--.....................................  121
PKH_113350   118 ..T....................................I............................................D--.....................................  120
PVX_113910   118 ..T....................................I............................................D--.....................................  120
PBANKA_123020 138 snY...................................H............................................H--.....................................  142
PY06538      138 snY....................................H............................................H--.....................................  142
PCHAS_123090 116 ..T....................................I............................................D--.....................................  118
TA09960      175 ..F....................................V............................................G--.....................................  177
TP04_0791    175 ..F....................................V............................................G--.....................................  177
BBOV_III008880 183 ..YvgphpgivdmlnqqlgdalkssgtevagrkdlqffpF............................................K--.....................................  221
TGGT1_118720 143 ..F....................................VdplprlarainqqrltwarnqqsalssqqfshlqeqlqkeelkpQ--.....................................  189
TGME49_029020 143 ..F....................................VdplprlarainqqrltwarnqqsalssqqfshlqeqlqkeelkpQ--.....................................  189
TGVEG_026470 143 ..F....................................VdplprlarainqqrltwarnqqsalssqqfshlqeqlqkeelkpQ--.....................................  189
NCLIV_030060 145 ..F....................................V............................................DPLprlaravnqqrvawarsqqsslssqqyshlqnqiqke  186
ETH_00023650 106 ..F....................................V............................................DQLprlveninqqriqwarqqhaaadresqeqlrrdlqtq  147
foreground (63):   V                                    H                                            S                                         6.0
                   I                                    Y                                            T
                                                        F                                            Q
wt_res_freqs (23): 1                                    1                                            1
                   3                                    1                                            1
                                                        1                                            1
insertions                                              4
deletions          1                                    1                                            199                                       9.1

Foreground
consensus     67 ..F....................................V............................................QQL.....................................  71*
PFF0750w     119 ..T....................................I............................................D--.....................................  121
PKH_113350   118 ..T....................................I............................................D--.....................................  120
PVX_113910   118 ..T....................................I............................................D--.....................................  120
PBANKA_123020 138 snY...................................H............................................H--.....................................  142
PY06538      138 snY....................................H............................................H--.....................................  142
PCHAS_123090 116 ..T....................................I............................................D--.....................................  118
TA09960      175 ..F....................................V............................................G--.....................................  177
TP04_0791    175 ..F....................................V............................................G--.....................................  177
BBOV_III008880 183 ..YvgphpgivdmlnqqlgdalkssgtevagrkdlqffpF............................................K--.....................................  221
TGGT1_118720 143 ..F....................................VdplprlarainqqrltwarnqqsalssqqfshlqeqlqkeelkpQ--.....................................  189
TGME49_029020 143 ..F....................................VdplprlarainqqrltwarnqqsalssqqfshlqeqlqkeelkpQ--.....................................  189
TGVEG_026470 143 ..F....................................VdplprlarainqqrltwarnqqsalssqqfshlqeqlqkeelkpQ--.....................................  189
NCLIV_030060 145 ..F....................................V............................................DPLprlaravnqqrvawarsqqsslssqqyshlqnqiqke  186
ETH_00023650 106 ..F....................................V............................................DQLprlveninqqriqwarqqhaaadresqeqlrrdlqtq  147
foreground (15):   T                                    H                                            GQL                                       0.9
                   F                                    I                                            H
                   Y                                    V                                            Q
wt_res_freqs (3):  2                                    1                                            111
                   5                                    2                                            1
                   2                                    5                                            1
insertions         6                                    2                                              1
deletions                                                                                             88                                       2.3
position                                                                                             70
```

```
Intermediate
unknown            72 .....QQK....................................IF..................................MA............Y...EYCPG......DL.....KKLIQ... 91*
unknown           122 .....RQK....................................LIseyinrqilqhytsnyhhhhhsklditplaadqkFI............FaayEYCDGg.....DL.....KKLIQ... 179
unknown           121 .....RQKciseyinrqilqhhasnfqhskkldmtplsadqkf.IF..................................AA............Y...EYCDAg.....DL.....KRLIQ... 176
unknown           121 .....RQKciseyinrqilqhhasnfqhsakldmtplaadqkf.IF..................................AA............Y...EYCDAg.....DL.....KRLIQ... 176
unknown           143 .....QVK....................................ID..................................MVplsldqkfifaaY...EYCDGg.....DL.....KKLIQktk 178
unknown           143 .....QVK....................................ID..................................MVplsldqkfifaaY...EYCDGg.....DL.....KKLIQ... 175
unknown           119 .....RQKgiseyinrqilqhytsnyhhqvkidmaplsldqkf.IF..................................AA............Y...EYCDGg.....DL.....KKLIQktk 177
unknown           178 .....EHEgitnklnnqiseliksdsrlspkefgfypfkntqifVL..................................GL............Y...EFCRGg.....DLgrgifRKFSK... 239
unknown           178 .....GDL....................................GR..................................GI............F...-----......--.....RKFTK... 190
unknown           222 .....KSQ....................................IF..................................MFal..........Y...EYCEGg.....DLgraiwRAYSK... 249
unknown           190 .....QNF....................................VF..................................AA............Y...EFCPGg.....DL.....KKLLA... 210
unknown           190 .....QNF....................................VF..................................AA............Y...EFCPGg.....DL.....KKLLA... 210
unknown           190 .....QNF....................................VF..................................AA............Y...EFCPGg.....DL.....KKLLA... 210
unknown           187 elkpnQNF....................................VF..................................AA............Y...EFCPGg.....DL.....KKLLA... 212
unknown           148 laqe.ELK....................................PT..................................QV............F...VFAAYelceggDL.....KKLLA... 177
position                                                  .                                                    80          .        90

Background
consensus          72 .....QQK....................................IF..................................MA............Y...EYCPG......DL.....KKLIQ... 91*
PFF0750w          122 .....RQK....................................LIseyinrqilqhytsnyhhhhhsklditplaadqkFI............FaayEYCDGg.....DL.....KKLIQ... 179
PKH_113350        121 .....RQKciseyinrqilqhhasnfqhskkldmtplsadqkf.IF..................................AA............Y...EYCDAg.....DL.....KRLIQ... 176
PVX_113910        121 .....RQKciseyinrqilqhhasnfqhsakldmtplaadqkf.IF..................................AA............Y...EYCDAg.....DL.....KRLIQ... 176
PBANKA_123020     143 .....QVK....................................ID..................................MVplsldqkfifaaY...EYCDGg.....DL.....KKLIQktk 178
PY06538           143 .....QVK....................................ID..................................MVplsldqkfifaaY...EYCDGg.....DL.....KKLIQ... 175
PCHAS_123090      119 .....RQKgiseyinrqilqhytsnyhhqvkidmaplsldqkf.IF..................................AA............Y...EYCDGg.....DL.....KKLIQktk 177
TA09960           178 .....EHEgitnklnnqiseliksdsrlspkefgfypfkntqifVL..................................GL............Y...EFCRGg.....DLgrgifRKFSK... 239
TP04_0791         178 .....GDL....................................GR..................................GI............F...-----......--.....RKFTK... 190
BBOV_III008880    222 .....KSQ....................................IF..................................MFal..........Y...EYCEGg.....DLgraiwRAYSK... 249
TGGT1_118720      190 .....QNF....................................VF..................................AA............Y...EFCPGg.....DL.....KKLLA... 210
TGME49_029020     190 .....QNF....................................VF..................................AA............Y...EFCPGg.....DL.....KKLLA... 210
TGVEG_026470      190 .....QNF....................................VF..................................AA............Y...EFCPGg.....DL.....KKLLA... 210
NCLIV_030060      187 elkpnQNF....................................VF..................................AA............Y...EFCPGg.....DL.....KKLLA... 212
ETH_00023650      148 laqe.ELK....................................PT..................................QV............F...VFAAYelceggDL.....KKLLA... 177
foreground (63):           NNK                                    VH                                  IV            F   EYCDM      DL     KKYIK    6.0
                           DSR                                    LY                                  LI            L    FMEY      N      REFLD
                           EGQ                                    IT                                  M                   L                  ME
wt_res_freqs (23):         112                                    11                                  16            6   74232      79     33533
                           111                                    53                                  52            1    3111      1      21142
                           111                                    21                                  1                   2                  11
insertions                 363                                                                                              1                3
deletions                  311                                    11                                  11            1   11111      11     11111    9.1

Foreground
consensus          72 .....QQK....................................IF..................................MA............Y...EYCPG......DL.....KKLIQ... 91*
PFF0750w          122 .....RQK....................................LIseyinrqilqhytsnyhhhhhsklditplaadqkFI............FaayEYCDGg.....DL.....KKLIQ... 179
PKH_113350        121 .....RQKciseyinrqilqhhasnfqhskkldmtplsadqkf.IF..................................AA............Y...EYCDAg.....DL.....KRLIQ... 176
PVX_113910        121 .....RQKciseyinrqilqhhasnfqhsakldmtplaadqkf.IF..................................AA............Y...EYCDAg.....DL.....KRLIQ... 176
PBANKA_123020     143 .....QVK....................................ID..................................MVplsldqkfifaaY...EYCDGg.....DL.....KKLIQktk 178
PY06538           143 .....QVK....................................ID..................................MVplsldqkfifaaY...EYCDGg.....DL.....KKLIQ... 175
PCHAS_123090      119 .....RQKgiseyinrqilqhytsnyhhqvkidmaplsldqkf.IF..................................AA............Y...EYCDGg.....DL.....KKLIQktk 177
TA09960           178 .....EHEgitnklnnqiseliksdsrlspkefgfypfkntqifVL..................................GL............Y...EFCRGg.....DLgrgifRKFSK... 239
TP04_0791         178 .....GDL....................................GR..................................GI............F...-----......--.....RKFTK... 190
BBOV_III008880    222 .....KSQ....................................IF..................................MFal..........Y...EYCEGg.....DLgraiwRAYSK... 249
TGGT1_118720      190 .....QNF....................................VF..................................AA............Y...EFCPGg.....DL.....KKLLA... 210
TGME49_029020     190 .....QNF....................................VF..................................AA............Y...EFCPGg.....DL.....KKLLA... 210
TGVEG_026470      190 .....QNF....................................VF..................................AA............Y...EFCPGg.....DL.....KKLLA... 210
NCLIV_030060      187 elkpnQNF....................................VF..................................AA............Y...EFCPGg.....DL.....KKLLA... 212
ETH_00023650      148 laqe.ELK....................................PT..................................QV............F...VFAAYelceggDL.....KKLLA... 177
foreground (15):           ENK                                    VT                                  GA            Y   EYCAG      DL     RKLSA    0.9
                           RLF                                    PF                                  MI            F   VFAPY              RFLK
                           QV                                     ID                                  QV                   DA                IQ
wt_res_freqs (3):          224                                    31                                  14            7   74716      99     27713
                           212                                    15                                  21            2   14121              1132
                           31                                     31                                  12                   31                33
insertions                   2                                     6                                   2            6       8       1         1
deletions                                                                                                               66666      66              2.3
position                                                  .                                                    80          .        90
```

```
Intermediate
unknown             92 .MHR.....A...................G..........LPLRHAKWLSFQLLNGLAYLHNNKMCHRDLKPENVML....NRTY....VLKIGDWGLCREFRYNDGDMTPTVCTIYYRPLEVL  172*
unknown            180 .KTKisddqA...................G..........LNLKEAKWLSFQLLNGLAYLHNNKMCHRDLKPENVMLqetsNHKY....LLKIGDLGLCREL-KNDGDMTPTVCTIYYRPLEVL  268
unknown            177 .KTKitddqA...................G..........LSLKEAKWLSFQLLNGLAYLHNNKMCHRDLKPENVMLqhtpNRKY....LLKIGDLGLCREL-KNDGDMTPTVCTIYYRPLEVL  265
unknown            177 .KTKitddqA...................G..........LSLKEAKWLSFQLLNGLAYLHNNKMCHRDLKPENVMLqqtaNRKF....LLKIGDLGLCREL-KNDGDMTPTVCTIYYRPLEVL  265
unknown            179 iSENq....A...................G..........LCLKEAKWLSFQLLNGLAYLHNNKMCHRDLKPENVMLqythNNKY....LLKIGDLGLCREL-KNDGDMTPTVCTIYYRPLEVL  264
unknown            176 .KTKinenqA...................G..........LCLKEAKWLSFQLLNGLAYLHNNKMCHRDLKPENVMLqythNNKY....LLKIGDLGLCREL-KNDGDMTPTVCTIYYRPLEVL  264
unknown            178 iSENq....A...................G..........LCLKEAKWLSFQLLNGLAYLHNNKMCHRDLKPENVMLqythNNKY....LLKIGDLGLCREL-KNDGDMTPTVCTIYYRPLEVL  263
unknown            240 .SSEg....-...................-..........FSLQEVKWLSFQLLNGLAYLHNLKIEHRDLKPNNIML....DREGpfp.VLKIGDWGLGREFRSLHGTITPTACTMFYRPIEVI  322
unknown            191 .SSEg....F...................S..........IPE--VKWLSFQLLNGLAYLHNLKIEHRDLKPNNIML....DRDGpfp.VLKIGDWGLGREFRSLHGTITPTACTMFYRPIEVI  273
unknown            250 .NQPg....Y...................S..........LSE--VKWYAFQLLNGLAYLHMSKIEHRDLKPNNIML....SDSErlt.VLKIGDWGMGREFRNLDGTVTPTACTLFYRPIEVI  332
unknown            211 .MHRq....Sagnqesgtgsppplsw...G..........LPLRHVKRLAFQLLNGLAFLHSENLCHRDLKPDNLML....TGTDpetaVLKIGDLGLCRELRYNVGDITPTVCTIYYRSLEVL  312
unknown            211 .MHRq....Sagnqesgtgsppplsw...G..........LPLRHVKRLAFQLLNGLAFLHSENLCHRDLKPDNLML....TGTDpetaVLKIGDLGLCRELRYNVGDITPTVCTIYYRSLEVL  312
unknown            211 .MHRq....Sagnqesgtgsppplsw...G..........LPLRHVKRLAFQLLNGLAFLHSENLCHRDLKPDNLML....TGTDpetaVLKIGDLGLCRELRYNVGDITPTVCTIYYRSLEVL  312
unknown            213 .LHRq....GaggnanqesgtgtppplswG..........LPLRHAKRLAFQLLNGLAFLHSENLCHRDLKPDNLML....TGTDpetaVLKIGDLGLCRELRYNVGDITPTVCTIYYRSLEVL  317
unknown            178 .KQRe....N...................KpsssvspewgLPLRHAKLLAFQLLNGLAFLHNEKLCHRDLKPDNLMLtse.NENC....VLKIGDLGLCRSFRANAGEITPTVCTIYYRPLEVL  272
position                        .                               100   .   110   .   120   .   130        .        140   .   150   .   160   .   170

Background
consensus           92 .MHR.....A...................G..........LPLRHAKWLSFQLLNGLAYLHNNKMCHRDLKPENVML....NRTY....VLKIGDWGLCREFRYNDGDMTPTVCTIYYRPLEVL  172*
PFF0750w           180 .KTKisddqA...................G..........LNLKEAKWLSFQLLNGLAYLHNNKMCHRDLKPENVMLqetsNHKY....LLKIGDLGLCREL-KNDGDMTPTVCTIYYRPLEVL  268
PKH_113350         177 .KTKitddqA...................G..........LSLKEAKWLSFQLLNGLAYLHNNKMCHRDLKPENVMLqhtpNRKY....LLKIGDLGLCREL-KNDGDMTPTVCTIYYRPLEVL  265
PVX_113910         177 .KTKitddqA...................G..........LSLKEAKWLSFQLLNGLAYLHNNKMCHRDLKPENVMLqqtaNRKF....LLKIGDLGLCREL-KNDGDMTPTVCTIYYRPLEVL  265
PBANKA_123020      179 iSENq....A...................G..........LCLKEAKWLSFQLLNGLAYLHNNKMCHRDLKPENVMLqythNNKY....LLKIGDLGLCREL-KNDGDMTPTVCTIYYRPLEVL  264
PY06538            176 .KTKinenqA...................G..........LCLKEAKWLSFQLLNGLAYLHNNKMCHRDLKPENVMLqythNNKY....LLKIGDLGLCREL-KNDGDMTPTVCTIYYRPLEVL  264
PCHAS_123090       178 iSENq....A...................G..........LCLKEAKWLSFQLLNGLAYLHNNKMCHRDLKPENVMLqythNNKY....LLKIGDLGLCREL-KNDGDMTPTVCTIYYRPLEVL  263
TA09960            240 .SSEg....-...................-..........FSLQEVKWLSFQLLNGLAYLHNLKIEHRDLKPNNIML....DREGpfp.VLKIGDWGLGREFRSLHGTITPTACTMFYRPIEVI  322
TP04_0791          191 .SSEg....F...................S..........IPE--VKWLSFQLLNGLAYLHNLKIEHRDLKPNNIML....DRDGpfp.VLKIGDWGLGREFRSLHGTITPTACTMFYRPIEVI  273
BBOV_III008880     250 .NQPg....Y...................S..........LSE--VKWYAFQLLNGLAYLHMSKIEHRDLKPNNIML....SDSErlt.VLKIGDWGMGREFRNLDGTVTPTACTLFYRPIEVI  332
TGGT1_118720       211 .MHRq....Sagnqesgtgsppplsw...G..........LPLRHVKRLAFQLLNGLAFLHSENLCHRDLKPDNLML....TGTDpetaVLKIGDLGLCRELRYNVGDITPTVCTIYYRSLEVL  312
TGME49_029020      211 .MHRq....Sagnqesgtgsppplsw...G..........LPLRHVKRLAFQLLNGLAFLHSENLCHRDLKPDNLML....TGTDpetaVLKIGDLGLCRELRYNVGDITPTVCTIYYRSLEVL  312
TGVEG_026470       211 .MHRq....Sagnqesgtgsppplsw...G..........LPLRHVKRLAFQLLNGLAFLHSENLCHRDLKPDNLML....TGTDpetaVLKIGDLGLCRELRYNVGDITPTVCTIYYRSLEVL  312
NCLIV_030060       213 .LHRq....GaggnanqesgtgtppplswG..........LPLRHAKRLAFQLLNGLAFLHSENLCHRDLKPDNLML....TGTDpetaVLKIGDLGLCRELRYNVGDITPTVCTIYYRSLEVL  317
ETH_00023650       178 .KQRe....N...................KpsssvspewgLPLRHAKLLAFQLLNGLAFLHNEKLCHRDLKPDNLMLtse.NENC....VLKIGDLGLCRSFRANAGEITPTVCTIYYRPLEVL  272
foreground (63):        SNP     K                   G          MDPNQVKSYMYQIIEGVHYCHSHRVIHRDIKPQNILV    NKNG    VVKIGDFGLARAFSIPVRTYSHEVVTLWYRAPEVL  6.0
                        MH                                     LTEELIRKFIF LLQALAFL QRGIL   L  S L L    DDD     NL LA    S SVT  LKKLTP II     S  LI
                        K                                      IS S L EILK   R IS I RN  M          I    SSK      I  S        G  IPP  K        P  I
wt_res_freqs (23):      122     1                   1          1211126311384116315493141299929949692    1225    22851999868252471315126648779949917
                        11                                     31112311211 48133123 12173   6  1 3 2    312     14 44    2 111  111271 12     1  41
                        1                                      11 1 1 1221   1 21 1 12  2          4    211      1  1        2  111  1        4  4
insertions              337                         7          1  1                                1    1143    91         3    6     1 1
deletions               611     2                   1          7666764331111111111111111111                  1 4    11113        3111     1111111111111  9.1

Foreground
consensus           92 .MHR.....A...................G..........LPLRHAKWLSFQLLNGLAYLHNNKMCHRDLKPENVML....NRTY....VLKIGDWGLCREFRYNDGDMTPTVCTIYYRPLEVL  172*
PFF0750w           180 .KTKisddqA...................G..........LNLKEAKWLSFQLLNGLAYLHNNKMCHRDLKPENVMLqetsNHKY....LLKIGDLGLCREL-KNDGDMTPTVCTIYYRPLEVL  268
PKH_113350         177 .KTKitddqA...................G..........LSLKEAKWLSFQLLNGLAYLHNNKMCHRDLKPENVMLqhtpNRKY....LLKIGDLGLCREL-KNDGDMTPTVCTIYYRPLEVL  265
PVX_113910         177 .KTKitddqA...................G..........LSLKEAKWLSFQLLNGLAYLHNNKMCHRDLKPENVMLqqtaNRKF....LLKIGDLGLCREL-KNDGDMTPTVCTIYYRPLEVL  265
PBANKA_123020      179 iSENq....A...................G..........LCLKEAKWLSFQLLNGLAYLHNNKMCHRDLKPENVMLqythNNKY....LLKIGDLGLCREL-KNDGDMTPTVCTIYYRPLEVL  264
PY06538            176 .KTKinenqA...................G..........LCLKEAKWLSFQLLNGLAYLHNNKMCHRDLKPENVMLqythNNKY....LLKIGDLGLCREL-KNDGDMTPTVCTIYYRPLEVL  264
PCHAS_123090       178 iSENq....A...................G..........LCLKEAKWLSFQLLNGLAYLHNNKMCHRDLKPENVMLqythNNKY....LLKIGDLGLCREL-KNDGDMTPTVCTIYYRPLEVL  263
TA09960            240 .SSEg....-...................-..........FSLQEVKWLSFQLLNGLAYLHNLKIEHRDLKPNNIML....DREGpfp.VLKIGDWGLGREFRSLHGTITPTACTMFYRPIEVI  322
TP04_0791          191 .SSEg....F...................S..........IPE--VKWLSFQLLNGLAYLHNLKIEHRDLKPNNIML....DRDGpfp.VLKIGDWGLGREFRSLHGTITPTACTMFYRPIEVI  273
BBOV_III008880     250 .NQPg....Y...................S..........LSE--VKWYAFQLLNGLAYLHMSKIEHRDLKPNNIML....SDSErlt.VLKIGDWGMGREFRNLDGTVTPTACTLFYRPIEVI  332
TGGT1_118720       211 .MHRq....Sagnqesgtgsppplsw...G..........LPLRHVKRLAFQLLNGLAFLHSENLCHRDLKPDNLML....TGTDpetaVLKIGDLGLCRELRYNVGDITPTVCTIYYRSLEVL  312
TGME49_029020      211 .MHRq....Sagnqesgtgsppplsw...G..........LPLRHVKRLAFQLLNGLAFLHSENLCHRDLKPDNLML....TGTDpetaVLKIGDLGLCRELRYNVGDITPTVCTIYYRSLEVL  312
TGVEG_026470       211 .MHRq....Sagnqesgtgsppplsw...G..........LPLRHVKRLAFQLLNGLAFLHSENLCHRDLKPDNLML....TGTDpetaVLKIGDLGLCRELRYNVGDITPTVCTIYYRSLEVL  312
NCLIV_030060       213 .LHRq....GaggnanqesgtgtppplswG..........LPLRHAKRLAFQLLNGLAFLHSENLCHRDLKPDNLML....TGTDpetaVLKIGDLGLCRELRYNVGDITPTVCTIYYRSLEVL  317
ETH_00023650       178 .KQRe....N...................KpsssvspewgLPLRHAKLLAFQLLNGLAFLHNEKLCHRDLKPDNLMLtse.NENC....VLKIGDLGLCRSFRANAGEITPTVCTIYYRPLEVL  272
foreground (15):        SSN     A                   G          LCLKEAKRLAFQLLNGLAYLHNNKICHRDLKPNNVML    TGTC    LLKIGDLGLCREFRANAGTITPTACTIYYRSLEVI  0.9
                        MHR     N                   K           PERHV L S        F  SLNME      E L      DRKY          W  G SL YLV EM   V  MF   I  L
                        KQK     S                   S           S     W              E L       D I      NEND                  K H D
wt_res_freqs (3):       211     3                   6          8183459294999999996996372799999929399    2221    39999979979836171925999299779927992
                        123     1                   1           41334 1 5        3  21232      3 3      1233          2  2 16 222 13   7  12   2  7
                        322     1                   1           2     6              3 3       3 2      4112                  3 1 6
insertions                9     2                   6                                              4       4
deletions                       6                   6             11                                                         4                      2.3
position                        .                               100   .   110   .   120   .   130        .        140   .   150   .   160   .   170
```

```
Intermediate
unknown           173 LG...................AA................H......................................................................................  177*
unknown           269 L-...................--................-skfelsnkhhhkshatnhrrddndddededddddedededdddeeeedddvdenvevklhgrnktssnnknnnnknhnsninynnh  353
unknown           266 L-...................--................-skfemakgkkgkvkargkakapvnglstcanpphgnnsqinhphasaphsnapypsraistrgidstgkkrihaasagctidrg  350
unknown           266 L-...................--................-skfemakgkkgrakargrvkpppggsranhgganrgganhsgenrcganrgsanhssaglananranesaphssapdaqrapst  350
unknown           265 L-...................--................-skfdrsktrsgkngvnksgsgsgsakngsvkngsvkngsvkngsvkngsvkngsvkngsvkngsikngsikngsikngs  349
unknown           265 L-...................--................-skfdrskmrppkngggsgsgsgnvsgkggggsgsgnvsskggggsgkggsgsasrksgsgggsgsskgrsgsggasgtvsrksg  349
unknown           264 L-...................--................-skfdrskarasksgsiksadiktgsnkngsnksgsnksgatnkssanksdtsksrnsnrsgasksgtsqngtsnrsggsksgns  348
unknown           323 LG...................SIsiltsdtnnskstfshN......................................................................................  343
unknown           274 LG...................SIsiltsdpnnlkttfshN......................................................................................  294
unknown           333 LG...................PM................Nippadpeaqlgsrplhrhn...................................................................  356
unknown           313 LGriqpandrdrakyaneng lAA................H......................................................................................  336
unknown           313 LGriqpandrdrakyanenglAA................H......................................................................................  336
unknown           313 LGriqpandrdrakyanenglAA................H......................................................................................  336
unknown           318 LGriqpandrdrakyanenglAA................H......................................................................................  341
unknown           273 LGrmkgdgeggseep......GA................H......................................................................................  290
position                                   .

Background
consensus         173 LG...................AA................H......................................................................................  177*
PFF0750w          269 L-...................--................-skfelsnkhhhkshatnhrrddndddededddddedededdddeeeedddvdenvevklhgrnktssnnknnnnknhnsninynnh  353
PKH_113350        266 L-...................--................-skfemakgkkgkvkargkakapvnglstcanpphgnnsqinhphasaphsnapypsraistrgidstgkkrihaasagctidrg  350
PVX_113910        266 L-...................--................-skfemakgkkgrakargrvkpppggsranhgganrgganhsgenrcganrgsanhssaglananranesaphssapdaqrapst  350
PBANKA_123020     265 L-...................--................-skfdrsktrsgkngvnksgsgsgsakngsvkngsvkngsvkngsvkngsvkngsvkngsvkngsikngsikngsikngs  349
PY06538           265 L-...................--................-skfdrskmrppkngggsgsgsgnvsgkggggsgsgnvsskggggsgkggsgsasrksgsgggsgsskgrsgsggasgtvsrksg  349
PCHAS_123090      264 L-...................--................-skfdrskarasksgsiksadiktgsnkngsnksgsnksgatnkssanksdtsksrnsnrsgasksgtsqngtsnrsggsksgns  348
TA09960           323 LG...................SIsiltsdtnnskstfshN......................................................................................  343
TP04_0791         274 LG...................SIsiltsdpnnlkttfshN......................................................................................  294
BBOV_III008880    333 LG...................PM................Nippadpeaqlgsrplhrhn...................................................................  356
TGGT1_118720      313 LGriqpandrdrakyanenglAA................H......................................................................................  336
TGME49_029020     313 LGriqpandrdrakyanenglAA................H......................................................................................  336
TGVEG_026470      313 LGriqpandrdrakyanenglAA................H......................................................................................  336
NCLIV_030060      318 LGriqpandrdrakyanenglAA................H......................................................................................  341
ETH_00023650      273 LGrmkgdgeggseep......GA................H......................................................................................  290
foreground (63):      LG                   AT                K                                                                                         6.0
                      F                    SR
                                           CK
wt_res_freqs (23):    67                   11                1
                      1                    21
                                           11
insertions
deletions              13                  11                1                                                                                         9.1

Foreground
consensus         173 LG...................AA................H......................................................................................  177*
PFF0750w          269 L-...................--................-skfelsnkhhhkshatnhrrddndddededddddedededdddeeeedddvdenvevklhgrnktssnnknnnnknhnsninynnh  353
PKH_113350        266 L-...................--................-skfemakgkkgkvkargkakapvnglstcanpphgnnsqinhphasaphsnapypsraistrgidstgkkrihaasagctidrg  350
PVX_113910        266 L-...................--................-skfemakgkkgrakargrvkpppggsranhgganrgganhsgenrcganrgsanhssaglananranesaphssapdaqrapst  350
PBANKA_123020     265 L-...................--................-skfdrsktrsgkngvnksgsgsgsakngsvkngsvkngsvkngsvkngsvkngsvkngsvkngsikngsikngsikngs  349
PY06538           265 L-...................--................-skfdrskmrppkngggsgsgsgnvsgkggggsgsgnvsskggggsgkggsgsasrksgsgggsgsskgrsgsggasgtvsrksg  349
PCHAS_123090      264 L-...................--................-skfdrskarasksgsiksadiktgsnkngsnksgsnksgatnkssanksdtsksrnsnrsgasksgtsqngtsnrsggsksgns  348
TA09960           323 LG...................SIsiltsdtnnskstfshN......................................................................................  343
TP04_0791         274 LG...................SIsiltsdpnnlkttfshN......................................................................................  294
BBOV_III008880    333 LG...................PM................Nippadpeaqlgsrplhrhn...................................................................  356
TGGT1_118720      313 LGriqpandrdrakyanenglAA................H......................................................................................  336
TGME49_029020     313 LGriqpandrdrakyanenglAA................H......................................................................................  336
TGVEG_026470      313 LGriqpandrdrakyanenglAA................H......................................................................................  336
NCLIV_030060      318 LGriqpandrdrakyanenglAA................H......................................................................................  341
ETH_00023650      273 LGrmkgdgeggseep......GA................H......................................................................................  290
foreground (15):      LG                   GA                N                                                                                         0.9
                                           SI                H
                                           A
wt_res_freqs (3):     96                   13                2
                                           11                3
                                           2
insertions             3                    1                4
deletions              4                   44                4                                                                                         2.3
position                                   .
```

Intermediate

```
unknown        178 ............................................................................................................................
unknown        354 nwsrrknmkkkkkkkkkkkkeqdddyynkdfq............................................................................................ 385
unknown        351 raegnggnrsdqgngnsnnrkkknnkergkethgerteegekytqrkrerarmrgieeddyynnkdfq........................................................ 418
unknown        351 rgihsvrssgdgggsavraacttdggtahgsgangsgdgqgtpcnrnrkskqrevaagrgkatarekaatrenaatqenaatqnaatqnaatqnagtqenaaaqnaaaqerarlrgieeddyys 474
unknown        350 ikngsgrcsegrrsddaymgdeafekdyesfndrdfq....................................................................................... 386
unknown        350 sgkgrsggasgsgggggtvrrsdeaymgdemfeknyesfndrdfq............................................................................... 394
unknown        349 ksrtsksgtsqngtstksngsgrgserrssddaymgdepfekeyesfndrdfq....................................................................... 401
unknown        344 ............................................................................................................................
unknown        295 ............................................................................................................................
unknown        357 ............................................................................................................................
unknown        337 ............................................................................................................................
unknown        337 ............................................................................................................................
unknown        337 ............................................................................................................................
unknown        342 ............................................................................................................................
unknown        291 ............................................................................................................................
position
```

Background

```
consensus      178 ............................................................................................................................
PFF0750w       354 nwsrrknmkkkkkkkkkkkkeqdddyynkdfq............................................................................................ 385
PKH_113350     351 raegnggnrsdqgngnsnnrkkknnkergkethgerteegekytqrkrerarmrgieeddyynnkdfq........................................................ 418
PVX_113910     351 rgihsvrssgdgggsavraacttdggtahgsgangsgdgqgtpcnrnrkskqrevaagrgkatarekaatrenaatqenaatqnaatqnaatqnagtqenaaaqnaaaqerarlrgieeddyys 474
PBANKA_123020  350 ikngsgrcsegrrsddaymgdeafekdyesfndrdfq....................................................................................... 386
PY06538        350 sgkgrsggasgsgggggtvrrsdeaymgdemfeknyesfndrdfq............................................................................... 394
PCHAS_123090   349 ksrtsksgtsqngtstksngsgrgserrssddaymgdepfekeyesfndrdfq....................................................................... 401
TA09960        344 ............................................................................................................................
TP04_0791      295 ............................................................................................................................
BBOV_III008880 357 ............................................................................................................................
TGGT1_118720   337 ............................................................................................................................
TGME49_029020  337 ............................................................................................................................
TGVEG_026470   337 ............................................................................................................................
NCLIV_030060   342 ............................................................................................................................
ETH_00023650   291 ............................................................................................................................
insertions
deletions                                                                                                                                           9.1
```

Foreground

```
consensus      178 ............................................................................................................................
PFF0750w       354 nwsrrknmkkkkkkkkkkkkeqdddyynkdfq............................................................................................ 385
PKH_113350     351 raegnggnrsdqgngnsnnrkkknnkergkethgerteegekytqrkrerarmrgieeddyynnkdfq........................................................ 418
PVX_113910     351 rgihsvrssgdgggsavraacttdggtahgsgangsgdgqgtpcnrnrkskqrevaagrgkatarekaatrenaatqenaatqnaatqnaatqnagtqenaaaqnaaaqerarlrgieeddyys 474
PBANKA_123020  350 ikngsgrcsegrrsddaymgdeafekdyesfndrdfq....................................................................................... 386
PY06538        350 sgkgrsggasgsgggggtvrrsdeaymgdemfeknyesfndrdfq............................................................................... 394
PCHAS_123090   349 ksrtsksgtsqngtstksngsgrgserrssddaymgdepfekeyesfndrdfq....................................................................... 401
TA09960        344 ............................................................................................................................
TP04_0791      295 ............................................................................................................................
BBOV_III008880 357 ............................................................................................................................
TGGT1_118720   337 ............................................................................................................................
TGME49_029020  337 ............................................................................................................................
TGVEG_026470   337 ............................................................................................................................
NCLIV_030060   342 ............................................................................................................................
ETH_00023650   291 ............................................................................................................................
insertions
deletions                                                                                                                                           2.3
position
```

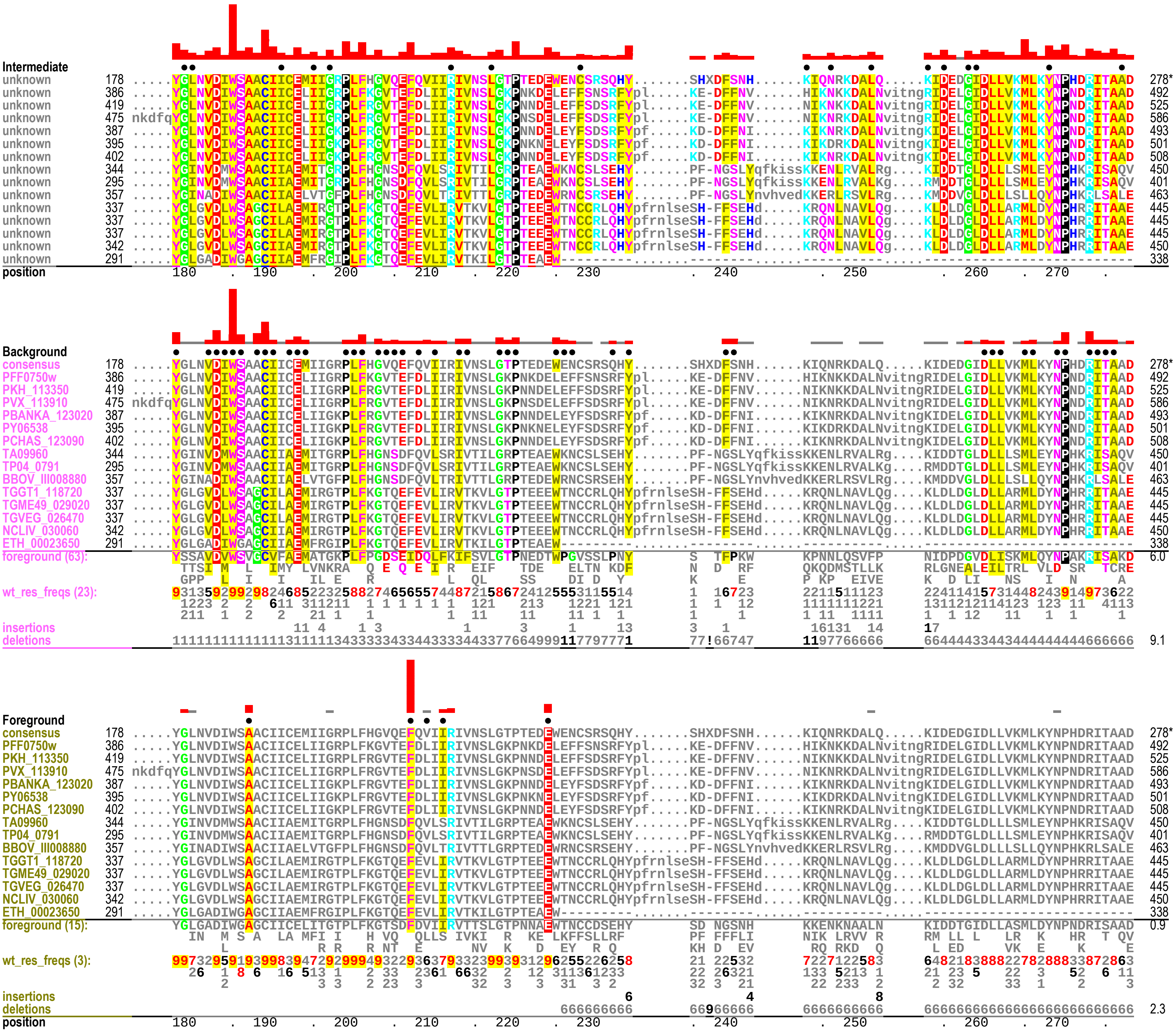
Intermediate
Background
consensus
PFF0750w
PKH_113350
PVX_113910
PBANKA_123020
PY06538
PCHAS_123090
TA09960
TP04_0791
BBOV_III008880
TGGT1_118720
TGME49_029020
TGVEG_026470
NCLIV_030060
ETH_00023650
foreground (63):
wt_res_freqs (23):
insertions
deletions
Foreground
foreground (15):
wt_res_freqs (3):
position

Intermediate

unknown	279	ALSHQWF	285*
unknown	493	ALSHPWF	499
unknown	526	ALSHPWF	532
unknown	587	ALSHPWF	593
unknown	494	ALSHPWF	500
unknown	502	ALSHPWF	508
unknown	509	ALSHPWF	515
unknown	451	ALSHRWF	457
unknown	402	ALSHKWF	408
unknown	464	ALSHPWF	470
unknown	446	ALSHQWF	452
unknown	446	ALSHQWF	452
unknown	446	ALSHQWF	452
unknown	451	ALSHQWF	457
unknown	339	-------	
position		.	

Background

		●● ●●●●	
consensus	279	ALSHQWF	285*
PFF0750w	493	ALSHPWF	499
PKH_113350	526	ALSHPWF	532
PVX_113910	587	ALSHPWF	593
PBANKA_123020	494	ALSHPWF	500
PY06538	502	ALSHPWF	508
PCHAS_123090	509	ALSHPWF	515
TA09960	451	ALSHRWF	457
TP04_0791	402	ALSHKWF	408
BBOV_III008880	464	ALSHPWF	470
TGGT1_118720	446	ALSHQWF	452
TGME49_029020	446	ALSHQWF	452
TGVEG_026470	446	ALSHQWF	452
NCLIV_030060	451	ALSHQWF	457
ETH_00023650	339	-------	
foreground (63):		ALNHPWF<br>L QD FL<br>K Y	6.0
wt_res_freqs (23):		7816616<br>1 11 21<br>1 4	
insertions			
deletions		6667111	9.1

Foreground

		●	
consensus	279	ALSHQWF	285*
PFF0750w	493	ALSHPWF	499
PKH_113350	526	ALSHPWF	532
PVX_113910	587	ALSHPWF	593
PBANKA_123020	494	ALSHPWF	500
PY06538	502	ALSHPWF	508
PCHAS_123090	509	ALSHPWF	515
TA09960	451	ALSHRWF	457
TP04_0791	402	ALSHKWF	408
BBOV_III008880	464	ALSHPWF	470
TGGT1_118720	446	ALSHQWF	452
TGME49_029020	446	ALSHQWF	452
TGVEG_026470	446	ALSHQWF	452
NCLIV_030060	451	ALSHQWF	457
ETH_00023650	339	-------	
foreground (15):		ALSHQWF<br>P	0.9
wt_res_freqs (3):		8888288<br>4	
insertions			
deletions		6666666	2.3
position		.	
