# Supplementary material for: Structural and evolutionary divergence of eukaryotic protein kinases in Apicomplexa
Source: BMC Evol Biol. 2011 Nov 2;11:321. doi: 10.1186/1471-2148-11-321 (PMC3239843; doi:10.1186/1471-2148-11-321)
Supplement: Additional file 9 — Alignment of selected CDPK subfamily and MAPK sequences. Annotated alignment of CDPK subfamily representatives CpCDPK2 and PfCDPK5 with human MAPK sequences p38, JNK1 and ERK1. GUIDANCE [100] was used to align the sequence segments, calculate reliability scores, and generate the initial version of the figure, to which we added further annotations. [file 1471-2148-11-321-S9.PDF]

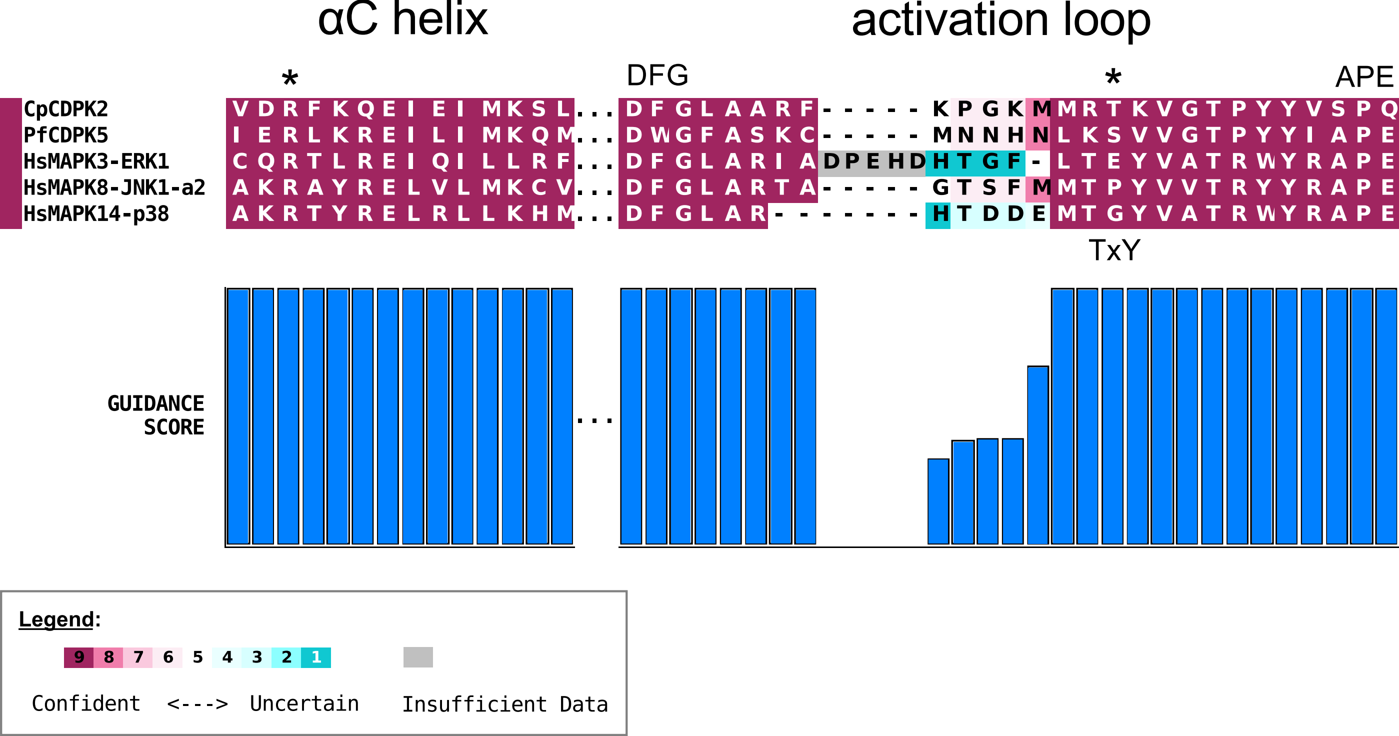

αC helix
activation loop
*
DFG
*
APE
CpCDPK2 V D R F K Q E I E I M K S L ... D F G L A A R F - - - - - - K P G K M M R T K V G T P Y Y V S P Q
PfCDPK5 I E R L K R E I L I M K Q M ... D W G F A S K C - - - - - - M N N H N L K S V V G T P Y Y I A P E
HsMAPK3-ERK1 C Q R T L R E I Q I L L R F ... D F G L A R I A D P E H D H T G F - L T E Y V A T R W Y R A P E
HsMAPK8-JNK1-a2 A K R A Y R E L V L M K C V ... D F G L A R T A - - - - - - G T S F M M T P Y V V T R Y Y R A P E
HsMAPK14-p38 A K R T Y R E L R L L K H M ... D F G L A R - - - - - - - - H T D D E M T G Y V A T R W Y R A P E
TxY
GUIDANCE SCORE
Legend:
9 8 7 6 5 4 3 2 1
Confident <---> Uncertain
Insufficient Data
