# Supplementary material for: Structural and evolutionary divergence of eukaryotic protein kinases in Apicomplexa
Source: BMC Evol Biol. 2011 Nov 2;11:321. doi: 10.1186/1471-2148-11-321 (PMC3239843; doi:10.1186/1471-2148-11-321)
Supplement: Additional file 11 — CLK family CHAIN alignment. Colorized sequence alignment and partition generated by the CHAIN program, comparing the apicomplexan-specific subfamily of CLKs to a diverse set of eukaryotic CLKs. [file 1471-2148-11-321-S11.PDF]

## Apicomplexan CLK vs. eukaryotic CLK

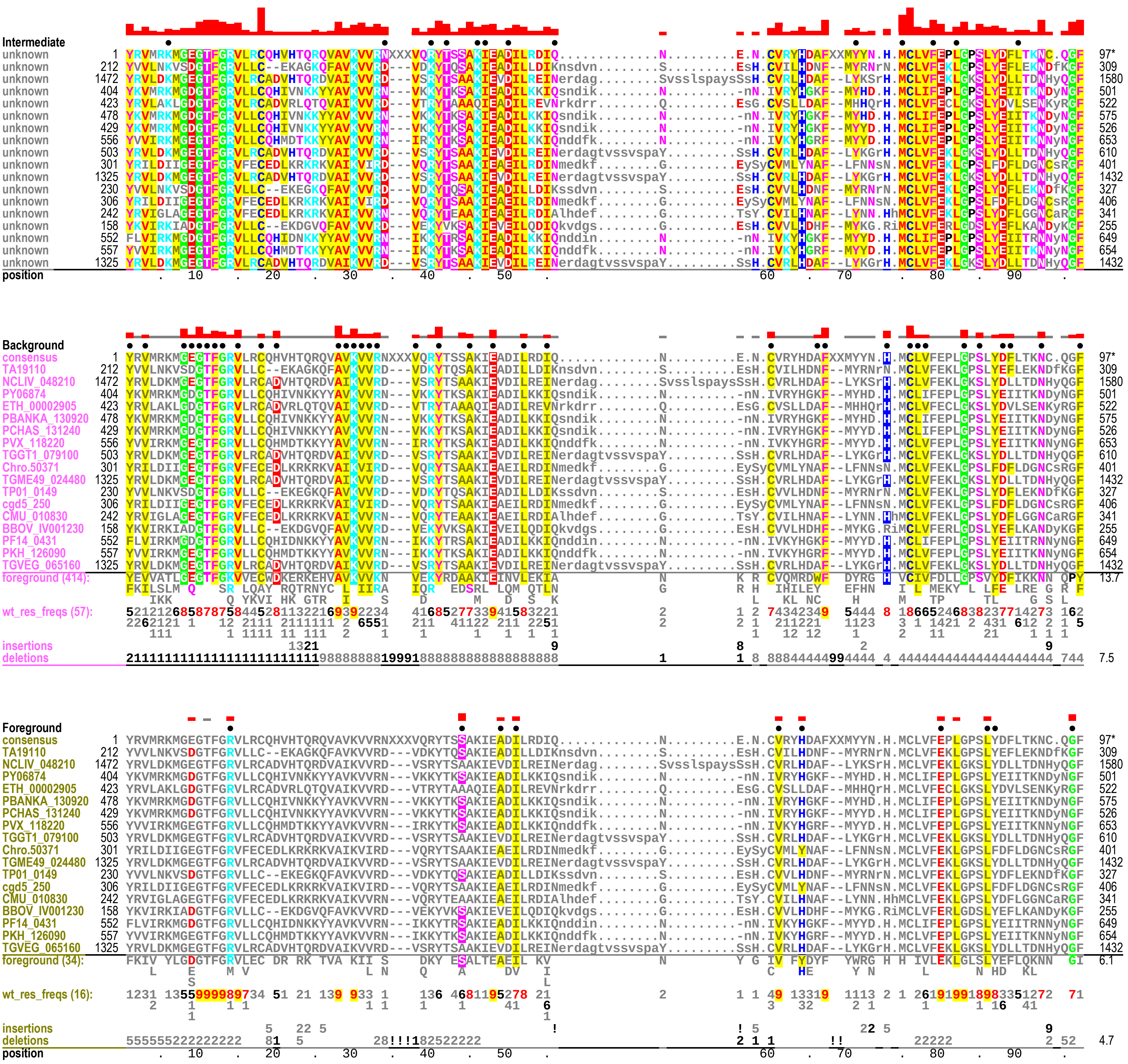

```
Intermediate
unknown              98 HLEDIQNYCIQCLWALSFLRKCKLTHTDLKPENILLD.........................ASTGIKIIDFGCATFEDXXDYXHGSIINTRQYRAPEVILNN..................LGW  178*
unknown             310 FISDIQNMAYQLLKGLSFLKKKRLVHTDIKPENILLVcgkddfievpfprshtgmmtkrp..AMSDIKLIDFGSAIYED--EY-HSSIINTRQYRAPEVILGNnlylsfmlneirvticvdIGW  428
unknown            1581 YLEDIRIVAKQCLIALAFLRVCRLTHTDLKPENILLLddvlipvpaprpsssssskgrylrpAKVGVKVIDFGSATFED--DY-HSSLINTRQYRAPEVILG-..................LGW  1682
unknown             502 HIEDIKLYCIEILKALHYLRKIKLTHTDLKPENILLDdphfekkivtvkrvtdgkkiqiyrsKSKGIKIIDFGCATFKS--DY-HGSIINTRQYRAPEVILN-..................LGW  603
unknown             523 YLEDIIQVAKQGLTALAFMRDCKLAHTDLKPENILLQgeemyetraprpseeddcstpflrpASMQVKIIDFGSATFEE--DY-HSSLINTRQYRAPEVILD-..................IGW  624
unknown             576 HIEDIKLYCIEILKALHYLRKLKLTHTDLKPENILLDdphfekkivtvkrvtdgkkiqiyrsKSKGIKIIDFGCATFKT--DY-HGSIINTRQYRAPEVILN-..................LGW  677
unknown             527 HIEDIKLYCIEILKALHYLRKIKLTHTDLKPENILLDdplfekkvvtvkrvtdgkriqiyrsKSKGIKIIDFGCATFKS--DY-HGSIINTRQYRAPEVILN-..................LGW  628
unknown             654 HLEDIRLYCIEMLKALSYLRKISLTHTDLKPENILLDdpyfektlvsvrratdgkrvqiyrtKSTGIKLIDFGCATFKD--GY-HGSIINTRQYRAPEVILN-..................LGW  755
unknown             611 YLEDIRTVAKQCLIALAFLRVCRLTHTDLKPENILLLddilipvsaprvtveqlvegsllrpAQVGVKIIDFGSATFED--DY-HSSLINTRQYRAPEVILG-..................LGW  712
unknown             402 FLADIQNISEQFLLALSFLRKMKLTHTDLKLENILFTdnnyiwvnaprhpgalirrp.....VRPEIRLIDFGAATYEH--DY-HGSVINTRQYRAPEVILD-..................LGW  498
unknown            1433 YLEDIRTVAKQCLIALAFLRVCRLTHTDLKPENILLLddilipvsaprpssssskgrylrp.AQVGVKIIDFGSATFED--DY-HSSLINTRQYRAPEVILG-..................LGW  1533
unknown             328 FISDIQNMAYQLLKGLSFLKKKRLVHTDIKPENILLVcgkddfievpfprshtgmttkrp..AMSDIKLIDFGSAIYED--EY-HSSIINTRQYRAPEVILD-..................IGW  427
unknown             407 FLADIQNISEQFLLALSFLRKMKLTHTDLKLENILFTdnnyiwvnaprhpgalirrp.....VRPEIRLIDFGAATYEH--DY-HGSVINTRQYRAPEVILD-..................LGW  503
unknown             342 LLADIQSIAEQMLWALAFLRKMKLTHTDLKLENILLVesgyiwvnaprhpgslirrp.....IRSEIRLIDFGSATYDD--DY-HGSIINTRQYRAPEVILD-..................IGW  438
unknown             256 FMSDIQRIAFQLLKGLSFLKQNKLIHTDLKPENVLLTcghddfievpfprsttgmltkrp..ATADIKIIDFGSTIYED--DY-HSSIINTRQYRSPEVILD-..................LGW  355
unknown             650 HIEDIKLYCIEILKALNYLRKMSLTHTDLKPENILLDdpyfekslitvrrvtdgkkiqiyrtKSTGIKLIDFGCATFKS--DY-HGSIINTRQYRAPEVILN-..................LGW  751
unknown             655 HLEDIKLYCIEMLKALNYLRKICLTHTDLKPENILLDdpyfektlvsvrratdgkrvdiyrtKSTGIKLIDFGCATFKD--GY-HGSIINTRQYRAPEVILN-..................LGW  756
unknown            1433 YLEDIRTVAKQCLIALAFLRVCRLTHTDLKPENILLLddilipvsaprpssssskgrylrp.AQVGVKIIDFGSATFED--DY-HSSLINTRQYRAPEVILG-..................LGW  1533
position                100    .  110    .  120    .  130                             .  140    .  150    .  160    .  170    .
```

```
Background
consensus            98 HLEDIQNYCIQCLWALSFLRKCKLTHTDLKPENILLD.........................ASTGIKIIDFGCATFEDXXDYXHGSIINTRQYRAPEVILNN..................LGW  178*
TA19110             310 FISDIQNMAYQLLKGLSFLKKKRLVHTDIKPENILLVcgkddfievpfprshtgmmtkrp..AMSDIKLIDFGSAIYED--EY-HSSIINTRQYRAPEVILGNnlylsfmlneirvticvdIGW  428
NCLIV_048210       1581 YLEDIRIVAKQCLIALAFLRVCRLTHTDLKPENILLLddvlipvpaprpsssssskgrylrpAKVGVKVIDFGSATFED--DY-HSSLINTRQYRAPEVILG-..................LGW  1682
PY06874             502 HIEDIKLYCIEILKALHYLRKIKLTHTDLKPENILLDdphfekkivtvkrvtdgkkiqiyrsKSKGIKIIDFGCATFKS--DY-HGSIINTRQYRAPEVILN-..................LGW  603
ETH_00002905        523 YLEDIIQVAKQGLTALAFMRDCKLAHTDLKPENILLQgeemyetraprpseeddcstpflrpASMQVKIIDFGSATFEE--DY-HSSLINTRQYRAPEVILD-..................IGW  624
PBANKA_130920       576 HIEDIKLYCIEILKALHYLRKLKLTHTDLKPENILLDdphfekkivtvkrvtdgkkiqiyrsKSKGIKIIDFGCATFKT--DY-HGSIINTRQYRAPEVILN-..................LGW  677
PCHAS_131240        527 HIEDIKLYCIEILKALHYLRKIKLTHTDLKPENILLDdplfekkvvtvkrvtdgkriqiyrsKSKGIKIIDFGCATFKS--DY-HGSIINTRQYRAPEVILN-..................LGW  628
PVX_118220          654 HLEDIRLYCIEMLKALSYLRKISLTHTDLKPENILLDdpyfektlvsvrratdgkrvqiyrtKSTGIKLIDFGCATFKD--GY-HGSIINTRQYRAPEVILN-..................LGW  755
TGGT1_079100        611 YLEDIRTVAKQCLIALAFLRVCRLTHTDLKPENILLLddilipvsaprvtveqlvegsllrpAQVGVKIIDFGSATFED--DY-HSSLINTRQYRAPEVILG-..................LGW  712
Chro.50371          402 FLADIQNISEQFLLALSFLRKMKLTHTDLKLENILFTdnnyiwvnaprhpgalirrp.....VRPEIRLIDFGAATYEH--DY-HGSVINTRQYRAPEVILD-..................LGW  498
TGME49_024480      1433 YLEDIRTVAKQCLIALAFLRVCRLTHTDLKPENILLLddilipvsaprpssssskgrylrp.AQVGVKIIDFGSATFED--DY-HSSLINTRQYRAPEVILG-..................LGW  1533
TP01_0149           328 FISDIQNMAYQLLKGLSFLKKKRLVHTDIKPENILLVcgkddfievpfprshtgmttkrp..AMSDIKLIDFGSAIYED--EY-HSSIINTRQYRAPEVILD-..................IGW  427
cgd5_250            407 FLADIQNISEQFLLALSFLRKMKLTHTDLKLENILFTdnnyiwvnaprhpgalirrp.....VRPEIRLIDFGAATYEH--DY-HGSVINTRQYRAPEVILD-..................LGW  503
CMU_010830          342 LLADIQSIAEQMLWALAFLRKMKLTHTDLKLENILLVesgyiwvnaprhpgslirrp.....IRSEIRLIDFGSATYDD--DY-HGSIINTRQYRAPEVILD-..................IGW  438
BBOV_IV001230       256 FMSDIQRIAFQLLKGLSFLKQNKLIHTDLKPENVLLTcghddfievpfprsttgmltkrp..ATADIKIIDFGSTIYED--DY-HSSIINTRQYRSPEVILD-..................LGW  355
PF14_0431           650 HIEDIKLYCIEILKALNYLRKMSLTHTDLKPENILLDdpyfekslitvrrvtdgkkiqiyrtKSTGIKLIDFGCATFKS--DY-HGSIINTRQYRAPEVILN-..................LGW  751
PKH_126090          655 HLEDIKLYCIEMLKALNYLRKICLTHTDLKPENILLDdpyfektlvsvrratdgkrvdiyrtKSTGIKLIDFGCATFKD--GY-HGSIINTRQYRAPEVILN-..................LGW  756
TGVEG_065160       1433 YLEDIRTVAKQCLIALAFLRVCRLTHTDLKPENILLLddilipvsaprpssssskgrylrp.AQVGVKIIDFGSATFED--DY-HSSLINTRQYRAPEVILG-..................LGW  1533
foreground (414):       PIDQVREIGYQLCEAVAYLHDLKIVHTDLKPENILFV                         KNSDVKVVDFGSATYDD  EH HSSVVSTRHYRAPEVVLG                   LGW  13.7
                        TLEHIQQMAK  LTSLRFM ENRLI          L                           DTEIRLI    T FEH  QY  TTLI      P  II E
                          SLL HFSR  FR  K     Q T                                       SPA VIC      L R  HD   YI
wt_res_freqs (57):      6222441312872236326944212999999998935                         42222341989875143  53 752277998997996295                   789
                        1213321251  3131171 23273          4                           1326446    1 612  12  1322      1  26 2
                          111 2212  11  1     1 3                                       211 111      1 1  11   14
insertions                                 4                9                                       1
deletions               4444444433344443332222222222222222223                         33322222222222222992292222222222222222229                  233  7.5
```

```
Foreground
consensus            98 HLEDIQNYCIQCLWALSFLRKCKLTHTDLKPENILLD.........................ASTGIKIIDFGCATFEDXXDYXHGSIINTRQYRAPEVILNN..................LGW  178*
TA19110             310 FISDIQNMAYQLLKGLSFLKKKRLVHTDIKPENILLVcgkddfievpfprshtgmmtkrp..AMSDIKLIDFGSAIYED--EY-HSSIINTRQYRAPEVILGNnlylsfmlneirvticvdIGW  428
NCLIV_048210       1581 YLEDIRIVAKQCLIALAFLRVCRLTHTDLKPENILLLddvlipvpaprpsssssskgrylrpAKVGVKVIDFGSATFED--DY-HSSLINTRQYRAPEVILG-..................LGW  1682
PY06874             502 HIEDIKLYCIEILKALHYLRKIKLTHTDLKPENILLDdphfekkivtvkrvtdgkkiqiyrsKSKGIKIIDFGCATFKS--DY-HGSIINTRQYRAPEVILN-..................LGW  603
ETH_00002905        523 YLEDIIQVAKQGLTALAFMRDCKLAHTDLKPENILLQgeemyetraprpseeddcstpflrpASMQVKIIDFGSATFEE--DY-HSSLINTRQYRAPEVILD-..................IGW  624
PBANKA_130920       576 HIEDIKLYCIEILKALHYLRKLKLTHTDLKPENILLDdphfekkivtvkrvtdgkkiqiyrsKSKGIKIIDFGCATFKT--DY-HGSIINTRQYRAPEVILN-..................LGW  677
PCHAS_131240        527 HIEDIKLYCIEILKALHYLRKIKLTHTDLKPENILLDdplfekkvvtvkrvtdgkriqiyrsKSKGIKIIDFGCATFKS--DY-HGSIINTRQYRAPEVILN-..................LGW  628
PVX_118220          654 HLEDIRLYCIEMLKALSYLRKISLTHTDLKPENILLDdpyfektlvsvrratdgkrvqiyrtKSTGIKLIDFGCATFKD--GY-HGSIINTRQYRAPEVILN-..................LGW  755
TGGT1_079100        611 YLEDIRTVAKQCLIALAFLRVCRLTHTDLKPENILLLddilipvsaprvtveqlvegsllrpAQVGVKIIDFGSATFED--DY-HSSLINTRQYRAPEVILG-..................LGW  712
Chro.50371          402 FLADIQNISEQFLLALSFLRKMKLTHTDLKLENILFTdnnyiwvnaprhpgalirrp.....VRPEIRLIDFGAATYEH--DY-HGSVINTRQYRAPEVILD-..................LGW  498
TGME49_024480      1433 YLEDIRTVAKQCLIALAFLRVCRLTHTDLKPENILLLddilipvsaprpssssskgrylrp.AQVGVKIIDFGSATFED--DY-HSSLINTRQYRAPEVILG-..................LGW  1533
TP01_0149           328 FISDIQNMAYQLLKGLSFLKKKRLVHTDIKPENILLVcgkddfievpfprshtgmttkrp..AMSDIKLIDFGSAIYED--EY-HSSIINTRQYRAPEVILD-..................IGW  427
cgd5_250            407 FLADIQNISEQFLLALSFLRKMKLTHTDLKLENILFTdnnyiwvnaprhpgalirrp.....VRPEIRLIDFGAATYEH--DY-HGSVINTRQYRAPEVILD-..................LGW  503
CMU_010830          342 LLADIQSIAEQMLWALAFLRKMKLTHTDLKLENILLVesgyiwvnaprhpgslirrp.....IRSEIRLIDFGSATYDD--DY-HGSIINTRQYRAPEVILD-..................IGW  438
BBOV_IV001230       256 FMSDIQRIAFQLLKGLSFLKQNKLIHTDLKPENVLLTcghddfievpfprsttgmltkrp..ATADIKIIDFGSTIYED--DY-HSSIINTRQYRSPEVILD-..................LGW  355
PF14_0431           650 HIEDIKLYCIEILKALNYLRKMSLTHTDLKPENILLDdpyfekslitvrrvtdgkkiqiyrtKSTGIKLIDFGCATFKS--DY-HGSIINTRQYRAPEVILN-..................LGW  751
PKH_126090          655 HLEDIKLYCIEMLKALNYLRKICLTHTDLKPENILLDdpyfektlvsvrratdgkrvdiyrtKSTGIKLIDFGCATFKD--GY-HGSIINTRQYRAPEVILN-..................LGW  756
TGVEG_065160       1433 YLEDIRTVAKQCLIALAFLRVCRLTHTDLKPENILLLddilipvsaprpssssskgrylrp.AQVGVKIIDFGSATFED--DY-HSSLINTRQYRAPEVILG-..................LGW  1533
foreground (34):        N SCVK FARQLIEAIAY HDNRLVHTDLKLENIV V                         K T IKVIDFGGA QAT  DH K TVINTRQYR PEV LGC                  LGW  6.1
                        P  E R      L SLEF  QL  I     P   L                             D   L  M S  FDH  E     IV                                E
                                    T  V                                                                         L
wt_res_freqs (16):      1 1131 13163113111 61116199999199981 1                         1 1 591996959 111  32 1 248999999 999 831                  249
                        3  1 3      7 3717  12  1     8   8                             1   3  1 1  531  6     41                                1
                                    1  1                                                                         1
insertions                                  5               !                                                                                    15
deletions                                                   5                         88822            !!  !                  9                  222  4.7
position                100    .  110    .  120    .  130                             .  140    .  150    .  160    .  170    .
```

**Intermediate**

```
unknown              179 DMSSDMWSFGCILMELYTGNLLFRTHEHMEHLAMMEKIIGPFPKNML.EA...A...........................S..GRKYYSPAS................................ 238*
unknown              429 SYSSDLWSLGCTLMELYTGHLLFRTHSHMEHLAMMEKTIGKFPQEVI.SS...A...........................KntQGKNYIN--............................... 488
unknown             1683 DMSSDVWSLGCILMELYTGNLLFRTHEHLEHLAMMERIIGPMPPNML.EA...Av..........................StdGRRYLSPSSlasnpsetlayppffeagedrdrrdkdrrde 1776
unknown              604 DVSSDMWSFGCILAELYTGNLLFKTHEHLEHLALMENIIEPIPKKMLyEA...T...........................KtnGYKYID---............................... 663
unknown              625 DMASDMWSLGCILMELYTGDVLFRTHEHLEHLAMMERIVEPFPKDML.MK...A...........................QkgAGKGYLATDs.............................. 687
unknown              678 DVSSDMWSFGCILAELYTGNLLFKTHEHLEHLALMENIIEPIPKKMLyEA...T...........................KtnGYKYID---............................... 737
unknown              629 DVSSDMWSFGCILAELYTGNLLFKTHEHLEHLALMESIIEPIPKKMLyEA...T...........................KtnGYKYID---............................... 688
unknown              756 DVSSDMWSFGCVLAEMYTGDLLFRTHEHLEHLALMEAIVQPIPKKMIcEA...V...........................RtnGAKYI----............................... 814
unknown              713 DMSSDVWSLGCILMELYTGNLLFRTHEHLEHLAMMEKIIGPMPANML.EA...Av..........................StdGRRYLSPASlgssggeeree.................... 786
unknown              499 DMSSDMWGFGCILMELYTGVLLFKTHEHMEHLAMVERIIEPFPDYML.KKaynS...........................SksGKKYV----............................... 559
unknown             1534 DMSSDVWSLGCILMELYTGNLLFRTHEHLEHLAMMEKIIGPMPANML.EA...Av..........................StdGRRYLSPASlgssggeeree.................... 1607
unknown              428 SYSSDLWSLGCTLMELYTGHLLFRTHSHMEHLAMMEKTLGKFPEEVI.NS...A...........................KntQGKNY----............................... 485
unknown              504 DMSSDMWGFGCILMELYTGVLLFKTHEHMEHLAMIEKIIEPFPDYML.KKaynS...........................SksGKKYV----............................... 564
unknown              439 DMSSDMWSFGCILMELYTGVLLFKTHEHLEHLAMMEEIIGPFPQYMF.HT...AymgtagkkyvvktscnqwkqskpssyrKgkRSDYF----............................... 523
unknown              356 SYASDMWSLGCILVELYTGDLLFGTHSHLEHLAMMERTVGPLPSSML.AE...Ar..........................NteGRDYL----............................... 414
unknown              752 DVSSDMWSFGCVLAELYTGSLLFRTHEHMEHLAMMESIIQPIPKNMLyEA...T...........................KtnGSKYV----............................... 810
unknown              757 DVSSDMWSFGCVLAEMYTGDLLFRTHEHLEHLALMEAIIQPIPKKMI.SD...Ai..........................RtnGAKYI----............................... 815
unknown             1534 DMSSDVWSLGCILMELYTGNLLFRTHEHLEHLAMMEKIIGPMPANML.EA...Av..........................StdGRRYLSPASlgssggeeree.................... 1607
position                       .  190    .  200    .  210    .  220    .                                   230    .
```

**Background**

```
consensus            179 DMSSDMWSFGCILMELYTGNLLFRTHEHMEHLAMMEKIIGPFPKNML.EA...A...........................S..GRKYYSPAS................................ 238*
TA19110              429 SYSSDLWSLGCTLMELYTGHLLFRTHSHMEHLAMMEKTIGKFPQEVI.SS...A...........................KntQGKNYIN--............................... 488
NCLIV_048210        1683 DMSSDVWSLGCILMELYTGNLLFRTHEHLEHLAMMERIIGPMPPNML.EA...Av..........................StdGRRYLSPSSlasnpsetlayppffeagedrdrrdkdrrde 1776
PY06874              604 DVSSDMWSFGCILAELYTGNLLFKTHEHLEHLALMENIIEPIPKKMLyEA...T...........................KtnGYKYID---............................... 663
ETH_00002905         625 DMASDMWSLGCILMELYTGDVLFRTHEHLEHLAMMERIVEPFPKDML.MK...A...........................QkgAGKGYLATDs.............................. 687
PBANKA_130920        678 DVSSDMWSFGCILAELYTGNLLFKTHEHLEHLALMENIIEPIPKKMLyEA...T...........................KtnGYKYID---............................... 737
PCHAS_131240         629 DVSSDMWSFGCILAELYTGNLLFKTHEHLEHLALMESIIEPIPKKMLyEA...T...........................KtnGYKYID---............................... 688
PVX_118220           756 DVSSDMWSFGCVLAEMYTGDLLFRTHEHLEHLALMEAIVQPIPKKMIcEA...V...........................RtnGAKYI----............................... 814
TGGT1_079100         713 DMSSDVWSLGCILMELYTGNLLFRTHEHLEHLAMMEKIIGPMPANML.EA...Av..........................StdGRRYLSPASlgssggeeree.................... 786
Chro.50371           499 DMSSDMWGFGCILMELYTGVLLFKTHEHMEHLAMVERIIEPFPDYML.KKaynS...........................SksGKKYV----............................... 559
TGME49_024480       1534 DMSSDVWSLGCILMELYTGNLLFRTHEHLEHLAMMEKIIGPMPANML.EA...Av..........................StdGRRYLSPASlgssggeeree.................... 1607
TP01_0149            428 SYSSDLWSLGCTLMELYTGHLLFRTHSHMEHLAMMEKTLGKFPEEVI.NS...A...........................KntQGKNY----............................... 485
cgd5_250             504 DMSSDMWGFGCILMELYTGVLLFKTHEHMEHLAMIEKIIEPFPDYML.KKaynS...........................SksGKKYV----............................... 564
CMU_010830           439 DMSSDMWSFGCILMELYTGVLLFKTHEHLEHLAMMEEIIGPFPQYMF.HT...AymgtagkkyvvktscnqwkqskpssyrKgkRSDYF----............................... 523
BBOV_IV001230        356 SYASDMWSLGCILVELYTGDLLFGTHSHLEHLAMMERTVGPLPSSML.AE...Ar..........................NteGRDYL----............................... 414
PF14_0431            752 DVSSDMWSFGCVLAELYTGSLLFRTHEHMEHLAMMESIIQPIPKNMLyEA...T...........................KtnGSKYV----............................... 810
PKH_126090           757 DVSSDMWSFGCVLAEMYTGDLLFRTHEHLEHLALMEAIIQPIPKKMI.SD...Ai..........................RtnGAKYI----............................... 815
TGVEG_065160        1534 DMSSDVWSLGCILMELYTGNLLFRTHEHLEHLAMMEKIIGPMPANML.EA...Av..........................StdGRRYLSPASlgssggeeree.................... 1607
foreground (414):        SYPCDVWSVGCIIVELYTGDTLYQTHDNLEHLAMMEKVLGPIPSHMV KK   A                           D  KQKYF                                    13.7
                          FAA M  I   LI YFL EA FD   EDK   HL QRI  KLDQRFI RR   S                              AER
                          QS  I       Y FC   F       SR            RF     L Q                                   SS
wt_res_freqs (57):       63679399299813954491381588564799789823695371251 23   1                           1  31667
                          111 2  5   71 111 22 81   211   11 153  1311213 22   1                              211
                          31  2       1 11   1       12            11     1 1                                   11
insertions                                                     41      1  7   4                           8    1
deletions                3333333333333333333444444444444444444444444444445 78   4                           5  118989999                          7.5
```

**Foreground**

```
consensus            179 DMSSDMWSFGCILMELYTGNLLFRTHEHMEHLAMMEKIIGPFPKNML.EA...A...........................S..GRKYYSPAS................................ 238*
TA19110              429 SYSSDLWSLGCTLMELYTGHLLFRTHSHMEHLAMMEKTIGKFPQEVI.SS...A...........................KntQGKNYIN--............................... 488
NCLIV_048210        1683 DMSSDVWSLGCILMELYTGNLLFRTHEHLEHLAMMERIIGPMPPNML.EA...Av..........................StdGRRYLSPSSlasnpsetlayppffeagedrdrrdkdrrde 1776
PY06874              604 DVSSDMWSFGCILAELYTGNLLFKTHEHLEHLALMENIIEPIPKKMLyEA...T...........................KtnGYKYID---............................... 663
ETH_00002905         625 DMASDMWSLGCILMELYTGDVLFRTHEHLEHLAMMERIVEPFPKDML.MK...A...........................QkgAGKGYLATDs.............................. 687
PBANKA_130920        678 DVSSDMWSFGCILAELYTGNLLFKTHEHLEHLALMENIIEPIPKKMLyEA...T...........................KtnGYKYID---............................... 737
PCHAS_131240         629 DVSSDMWSFGCILAELYTGNLLFKTHEHLEHLALMESIIEPIPKKMLyEA...T...........................KtnGYKYID---............................... 688
PVX_118220           756 DVSSDMWSFGCVLAEMYTGDLLFRTHEHLEHLALMEAIVQPIPKKMIcEA...V...........................RtnGAKYI----............................... 814
TGGT1_079100         713 DMSSDVWSLGCILMELYTGNLLFRTHEHLEHLAMMEKIIGPMPANML.EA...Av..........................StdGRRYLSPASlgssggeeree.................... 786
Chro.50371           499 DMSSDMWGFGCILMELYTGVLLFKTHEHMEHLAMVERIIEPFPDYML.KKaynS...........................SksGKKYV----............................... 559
TGME49_024480       1534 DMSSDVWSLGCILMELYTGNLLFRTHEHLEHLAMMEKIIGPMPANML.EA...Av..........................StdGRRYLSPASlgssggeeree.................... 1607
TP01_0149            428 SYSSDLWSLGCTLMELYTGHLLFRTHSHMEHLAMMEKTLGKFPEEVI.NS...A...........................KntQGKNY----............................... 485
cgd5_250             504 DMSSDMWGFGCILMELYTGVLLFKTHEHMEHLAMIEKIIEPFPDYML.KKaynS...........................SksGKKYV----............................... 564
CMU_010830           439 DMSSDMWSFGCILMELYTGVLLFKTHEHLEHLAMMEEIIGPFPQYMF.HT...AymgtagkkyvvktscnqwkqskpssyrKgkRSDYF----............................... 523
BBOV_IV001230        356 SYASDMWSLGCILVELYTGDLLFGTHSHLEHLAMMERTVGPLPSSML.AE...Ar..........................NteGRDYL----............................... 414
PF14_0431            752 DVSSDMWSFGCVLAELYTGSLLFRTHEHMEHLAMMESIIQPIPKNMLyEA...T...........................KtnGSKYV----............................... 810
PKH_126090           757 DVSSDMWSFGCVLAEMYTGDLLFRTHEHLEHLALMEAIIQPIPKKMI.SD...Ai..........................RtnGAKYI----............................... 815
TGVEG_065160        1534 DMSSDVWSLGCILMELYTGNLLFRTHEHLEHLAMMEKIIGPMPANML.EA...Av..........................StdGRRYLSPASlgssggeeree.................... 1607
foreground (34):         NTPSDVWSIGCILVE FSGDLLFQTGNN EHLAIMERVFG IPSS I KR                                  A KYFD                                   6.1
                         DY   I     L    YL  V I VHD  M   M Q  L  L QN                                        L R
                         S                                                                                   K
wt_res_freqs (16):       111992962696529 119177826115 79791481115 3811 1 12                                  1 3441
                         41   2     1    81  1 1 151  1   4 1  1  1 11                                         1 1
                         2                                                                                   1
insertions                                          11         1       2  5   2                           9  2      8
deletions                22222222222222222222222222222222222222222222225 22   2                           8  555587888                          4.7
position                       .  190    .  200    .  210    .  220    .                                   230    .
```

```
Intermediate
unknown           239 ....N..............................SI.RVK..VPLQNLV...XXHHRLFCDFVYYILQIDPQARPTPEDALKHPFF 285*
unknown           489 ....Rdeprldwpegsksks...............SVhRVEecKTIMELVkp.--EHRLFGEFIRYILNLDSNKRPTPEEAMQHEFF 553
unknown          1777 reqtTsrlrlhwpegasstn...............SEeRVRncVPLHALVlp.--HHRAFADFVSSLLQIDPQARPTPGDALLHPFF 1845
unknown           664 ....Kknlrlawpenassid...............SIkHVKksLPLYNIIk..---HDLFCDFLYNILRIDPTRRASPIDLLKHEF- 725
unknown           688 ....Rggvhlkwpegaaslg...............SVnRVKncVPLEDLVlpq---DRILAEFVRYLLQVDPRKRPEPKEALRHPIF 752
unknown           738 ....Kndlklawpenassid...............SIkHVKksLPLYKIIk..---HDLFCDFLYTILRIDPTLRASPVDLLKHEF- 799
unknown           689 ....Kndlrlawpenassid...............SIkHVKrsLPLYKII...--RHDLFCDFLYTILRIDPTRRASPIDLLKHEF- 750
unknown           815 ....Nrdglrlawpenassfe..............SIkYVKrcLPLHKLI...--RNDLFCDFLYSILQIDPALRPAPAELLKHRF- 877
unknown           787 ....Ssrlrlnwpegssstn...............SEeRVRncVPLQALVlp.--HHRIFSDFVASLLQIDPQARPTPGDALMHPFF 851
unknown           560 ....NrlkisnninhhqneeykyvlnwpegsssntSIkRVNdcRPLENLVhk.--KHIMLAKFIRYILRPDPNLRPTPEMALRHEFF 639
unknown          1608 ....Ssrlrlnwpegssstn...............SEeRVRncVPLQALVlp.--HHRIFSDFVASLLQIDPQARPTPGDALMHPFF 1672
unknown           486 ....Ispneprlnwpegaksks.............SVhRVEecKTIMDLVkp.--EHRLFGEFIRYILNLDSNKRPTPEEAMQHEFF 552
unknown           565 ....NrlkisnnnnhhpseeykyilnwpegsssntSIkRVNdcRPLENLVhk.--KHIMLAKFIRYILRPDPNLRPTPEMALRHEFF 644
unknown           524 ....Kqstiptnytescdyeyqlyweeshinps..SFaRVQrcQPIEDYIhp.--KHKVFASFVRYILNLDPKLRPTPEMALKHEFF 601
unknown           415 ....Hptkdslnwpegaqsss..............SEeRVSccRSVMEIIrp.--EHRPFAEFIKYILNPDPSQRPTPEDAMEHEFF 480
unknown           811 ....Nkdelklawpenassin..............SIkHVKkcLPLYKIIk..---HELFCDFLYSILQIDPTLRPSPAELLKHKF- 873
unknown           816 ....Nrdhlrlawpenassfe..............SIkYVKrcLPLYKLI...--RNDLFCDFLYSVLQIDPALRSSPAELLKHRF- 878
unknown          1608 ....Ssrlrlnwpegssstn...............SEeRVRncVPLQALVlp.--HHRIFSDFVASLLQIDPQARPTPGDALMHPFF 1672
position                                              240         .  250        .  260     .  270     .  280    .
```

```
Background
consensus         239 ....N..............................SI.RVK..VPLQNLV...XXHHRLFCDFVYYILQIDPQARPTPEDALKHPFF 285*
TA19110           489 ....Rdeprldwpegsksks...............SVhRVEecKTIMELVkp.--EHRLFGEFIRYILNLDSNKRPTPEEAMQHEFF 553
NCLIV_048210     1777 reqtTsrlrlhwpegasstn...............SEeRVRncVPLHALVlp.--HHRAFADFVSSLLQIDPQARPTPGDALLHPFF 1845
PY06874           664 ....Kknlrlawpenassid...............SIkHVKksLPLYNIIk..---HDLFCDFLYNILRIDPTRRASPIDLLKHEF- 725
ETH_00002905      688 ....Rggvhlkwpegaaslg...............SVnRVKncVPLEDLVlpq---DRILAEFVRYLLQVDPRKRPEPKEALRHPIF 752
PBANKA_130920     738 ....Kndlklawpenassid...............SIkHVKksLPLYKIIk..---HDLFCDFLYTILRIDPTLRASPVDLLKHEF- 799
PCHAS_131240      689 ....Kndlrlawpenassid...............SIkHVKrsLPLYKII...--RHDLFCDFLYTILRIDPTRRASPIDLLKHEF- 750
PVX_118220        815 ....Nrdglrlawpenassfe..............SIkYVKrcLPLHKLI...--RNDLFCDFLYSILQIDPALRPAPAELLKHRF- 877
TGGT1_079100      787 ....Ssrlrlnwpegssstn...............SEeRVRncVPLQALVlp.--HHRIFSDFVASLLQIDPQARPTPGDALMHPFF 851
Chro.50371        560 ....NrlkisnninhhqneeykyvlnwpegsssntSIkRVNdcRPLENLVhk.--KHIMLAKFIRYILRPDPNLRPTPEMALRHEFF 639
TGME49_024480    1608 ....Ssrlrlnwpegssstn...............SEeRVRncVPLQALVlp.--HHRIFSDFVASLLQIDPQARPTPGDALMHPFF 1672
TP01_0149         486 ....Ispneprlnwpegaksks.............SVhRVEecKTIMDLVkp.--EHRLFGEFIRYILNLDSNKRPTPEEAMQHEFF 552
cgd5_250          565 ....NrlkisnnnnhhpseeykyilnwpegsssntSIkRVNdcRPLENLVhk.--KHIMLAKFIRYILRPDPNLRPTPEMALRHEFF 644
CMU_010830        524 ....Kqstiptnytescdyeyqlyweeshinps..SFaRVQrcQPIEDYIhp.--KHKVFASFVRYILNLDPKLRPTPEMALKHEFF 601
BBOV_IV001230     415 ....Hptkdslnwpegaqsss..............SEeRVSccRSVMEIIrp.--EHRPFAEFIKYILNPDPSQRPTPEDAMEHEFF 480
PF14_0431         811 ....Nkdelklawpenassin..............SIkHVKkcLPLYKIIk..---HELFCDFLYSILQIDPTLRPSPAELLKHKF- 873
PKH_126090        816 ....Nrdhlrlawpenassfe..............SIkYVKrcLPLYKLI...--RNDLFCDFLYSVLQIDPALRSSPAELLKHRF- 878
TGVEG_065160     1608 ....Ssrlrlnwpegssstn...............SEeRVRncVPLQALVlp.--HHRIFSDFVASLLQIDPQARPTPGDALMHPFF 1672
foreground (414):         Y                              SV AVK  KPLKDYI   DEDHGELFDLVQKMLEYDPAKRITAKEALQHPWF 13.7
                          R                              YI Y R  RR REFM   SHEDRQFL  LRRLFVFE SE L LRQ  R   F
                          N                                 R E     QNVL     F  L I  IKG  R    Q     D  K   Y
wt_res_freqs (57):        1                              33 132  4472123   3113115368122473667239475257718617
                          2                              32 1 1  12 2212   11211131  42131111 11 2 211  1  5
                          1                                 1 1     1111     1  1 1  312  1    1     1  1  1
insertions                9                              8   19       48    21  1
deletions                 2                              88 549  8888888   1188888888888888888888888888888891 7.5
```

```
Foreground
consensus         239 ....N..............................SI.RVK..VPLQNLV...XXHHRLFCDFVYYILQIDPQARPTPEDALKHPFF 285*
TA19110           489 ....Rdeprldwpegsksks...............SVhRVEecKTIMELVkp.--EHRLFGEFIRYILNLDSNKRPTPEEAMQHEFF 553
NCLIV_048210     1777 reqtTsrlrlhwpegasstn...............SEeRVRncVPLHALVlp.--HHRAFADFVSSLLQIDPQARPTPGDALLHPFF 1845
PY06874           664 ....Kknlrlawpenassid...............SIkHVKksLPLYNIIk..---HDLFCDFLYNILRIDPTRRASPIDLLKHEF- 725
ETH_00002905      688 ....Rggvhlkwpegaaslg...............SVnRVKncVPLEDLVlpq---DRILAEFVRYLLQVDPRKRPEPKEALRHPIF 752
PBANKA_130920     738 ....Kndlklawpenassid...............SIkHVKksLPLYKIIk..---HDLFCDFLYTILRIDPTLRASPVDLLKHEF- 799
PCHAS_131240      689 ....Kndlrlawpenassid...............SIkHVKrsLPLYKII...--RHDLFCDFLYTILRIDPTRRASPIDLLKHEF- 750
PVX_118220        815 ....Nrdglrlawpenassfe..............SIkYVKrcLPLHKLI...--RNDLFCDFLYSILQIDPALRPAPAELLKHRF- 877
TGGT1_079100      787 ....Ssrlrlnwpegssstn...............SEeRVRncVPLQALVlp.--HHRIFSDFVASLLQIDPQARPTPGDALMHPFF 851
Chro.50371        560 ....NrlkisnninhhqneeykyvlnwpegsssntSIkRVNdcRPLENLVhk.--KHIMLAKFIRYILRPDPNLRPTPEMALRHEFF 639
TGME49_024480    1608 ....Ssrlrlnwpegssstn...............SEeRVRncVPLQALVlp.--HHRIFSDFVASLLQIDPQARPTPGDALMHPFF 1672
TP01_0149         486 ....Ispneprlnwpegaksks.............SVhRVEecKTIMDLVkp.--EHRLFGEFIRYILNLDSNKRPTPEEAMQHEFF 552
cgd5_250          565 ....NrlkisnnnnhhpseeykyilnwpegsssntSIkRVNdcRPLENLVhk.--KHIMLAKFIRYILRPDPNLRPTPEMALRHEFF 644
CMU_010830        524 ....Kqstiptnytescdyeyqlyweeshinps..SFaRVQrcQPIEDYIhp.--KHKVFASFVRYILNLDPKLRPTPEMALKHEFF 601
BBOV_IV001230     415 ....Hptkdslnwpegaqsss..............SEeRVSccRSVMEIIrp.--EHRPFAEFIKYILNPDPSQRPTPEDAMEHEFF 480
PF14_0431         811 ....Nkdelklawpenassin..............SIkHVKkcLPLYKIIk..---HELFCDFLYSILQIDPTLRPSPAELLKHKF- 873
PKH_126090        816 ....Nrdhlrlawpenassfe..............SIkYVKrcLPLYKLI...--RNDLFCDFLYSVLQIDPALRSSPAELLKHRF- 878
TGVEG_065160     1608 ....Ssrlrlnwpegssstn...............SEeRVRncVPLQALVlp.--HHRIFSDFVASLLQIDPQARPTPGDALMHPFF 1672
foreground (34):          D                              V Y      SLYKIV        DLIDLIQNLFELEH KRITAKEA DHPFF 6.1
                                                                  P   LI                ML IDP      R I
                                                                  Q   M
wt_res_freqs (16):        1                              1 1      161122        15166211211111 29342135 18197
                                                                  4   56                47 778      1 1
                                                                  1   1
insertions                9                              9    9        8
deletions                 5                              22 222  2222222   9938552222222222222222222222225553 4.7
position                                              240         .  250        .  260     .  270     .  280    .
```
